# Active Site Loop Engineering Abolishes Water Capture in Hydroxylating Sesquiterpene Synthases

**DOI:** 10.1021/acscatal.3c03920

**Published:** 2023-10-20

**Authors:** Prabhakar L. Srivastava, Sam T. Johns, Rebecca Walters, David J. Miller, Marc W. Van der Kamp, Rudolf K. Allemann

**Affiliations:** † School of Chemistry, Cardiff University, Main Building, Park Place, Cardiff, CF10 3AT, United Kingdom; ‡ School of Biochemistry, University of Bristol, University Walk, Bristol, BS8 1TD, United Kingdom

**Keywords:** terpene synthase, hydroxylation, enzyme engineering, α-bulnesene, molecular dynamics

## Abstract

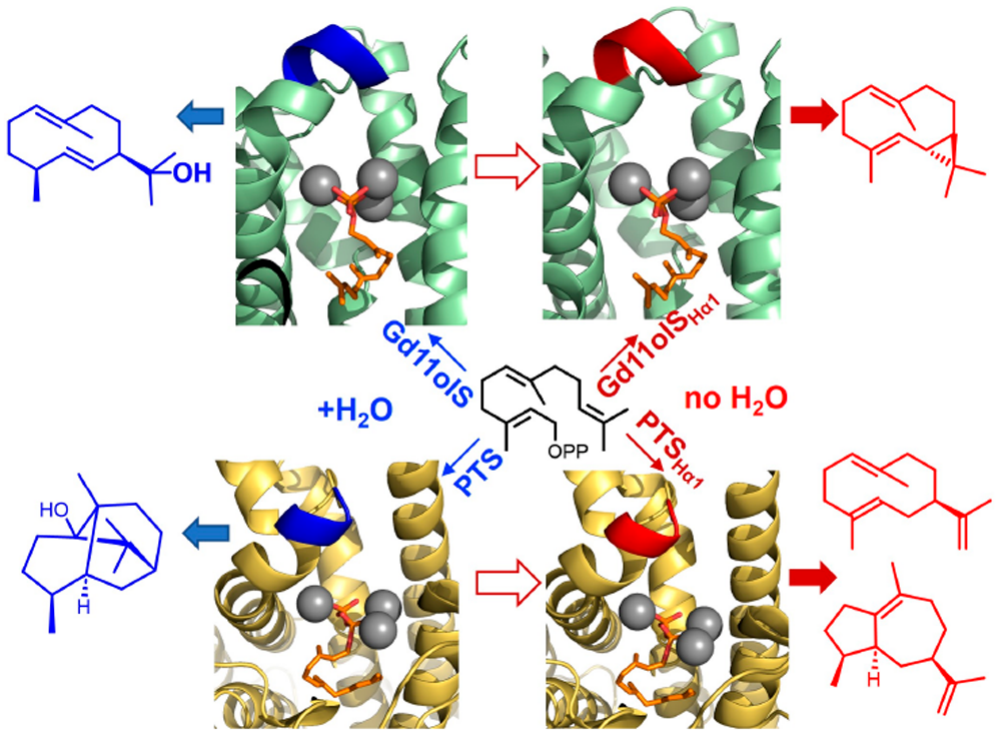

Terpene synthases (TS) catalyze complex reactions to produce a diverse array of terpene skeletons from linear isoprenyl diphosphates. Patchoulol synthase (PTS) from *Pogostemon cablin* converts farnesyl diphosphate into patchoulol. Using simulation-guided engineering, we obtained PTS variants that eliminate water capture. Further, we demonstrate that modifying the structurally conserved Hα-1 loop also reduces hydroxylation in PTS, as well as in germacradiene-11-ol synthase (Gd11olS), leading to cyclic neutral intermediates as products, including α-bulnesene (PTS) and isolepidozene (Gd11olS). Hα-1 loop modification could be a general strategy for engineering sesquiterpene synthases to produce complex cyclic hydrocarbons without the need for structure determination or modeling.

Terpenoids, the largest group of natural products with a broad spectrum of biological functions, are biosynthesized from a small number of acyclic prenyl diphosphates by terpene synthases (TSs).^1−4^ In the structurally conserved Class I TSs, the diphosphate (PPi) of the substrate is coordinated by three Mg^2+^ ions, with the hydrocarbon chain residing in a hydrophobic active site cavity.^1,5^ Abstraction of PPi leads to a carbocation that can react further to form a myriad of complex (poly)cyclic hydrocarbons, with the final cation quenched by deprotonation or water capture depending on the specific TS and active site cavity.^6−9^ Structural and mechanistic studies have revealed that, despite large differences in sequence, Class I (sesqui)TSs share a highly conserved fold and use similar chemical strategies to achieve the transformation of farnesyl diphosphate (FDP) into diverse C_15_ terpene products.^1,10−12^ While much progress has been made to decipher the biochemical details of (sesqui)TSs, altering the water capture behavior with targeted engineering remains challenging.^13^ Understanding carbocation management in TSs would enable targeted engineering to obtain specific (novel) terpene products.

Patchoulol synthase (PTS) is a Class I (sesqui)TS that produces patchoulol as the main product upon incubation with FDP.^14^ PTS is the key enzyme in patchouli oil biosynthesis,^14,15^ a widely appreciated natural fragrance commonly used in the cosmetics industry. The common non-hydroxylated PTS side-product α-bulnesene may have potential as an anti-platelet aggregation agent (antagonist of PAF, the platelet-activating factor).^16^ Here, we report a combined computational and experimental approach to gain insights into patchoulol biosynthesis. We further demonstrate a strategy to engineer hydroxylating (sesqui)TSs to form complex non-hydroxylated (sesqui)terpenes: Hα-1 loop modification. A four-residue replacement leads to the formation of primarily isolepidozene (**24**) in germacradiene-11-ol synthase (Gd11olS) and a mixture of α-bulnesene (**4**) and germacrene A (**3**) in PTS.

First, we characterized the product profile and kinetics of wild-type PTS (PTS_WT_) from *P. cablin* (details in Supporting Information). GCMS analysis indicated formation of patchoulol as major product (**2**, 60%), alongside several sesquiterpene hydrocarbons from FDP (Table S2), similar to the profile reported earlier.^14,17^ Kinetic constants for PTS (Table 1) were slightly different than the reported values (*K*_M_ 4.0 μM and *k*_cat_ 4.0 × 10^–4^ s^–1^).^14^ The majority of the products identified (98%) logically derive from the germacryl cation,^17^ while trace compounds (2%) likely arise from the humulyl cation (Scheme 1), suggesting multiple mechanisms operating concurrently.

**Scheme 1:**
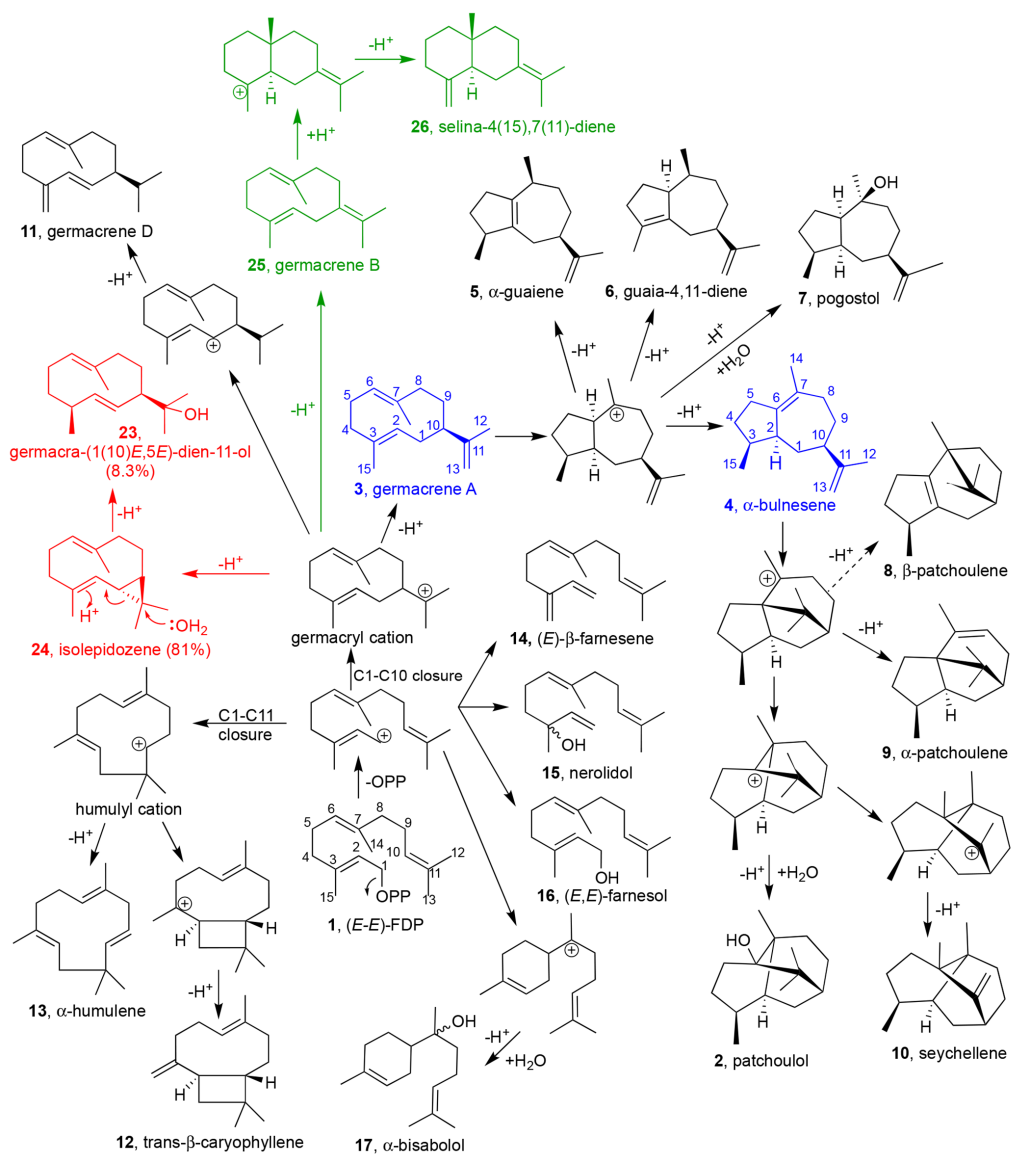
Proposed Pathways for Formation of Sesquiterpenes by PTS Variants Blue products are putative neutral on-path intermediates, red products are Gd11olS_Hα-1_ (with % formed), and green products are SdS_Hα-1_ variants.

**Table 1:** Kinetic Constants of PTS_WT_ and Its Variants

	*K*_M_ (μM)	*k*_cat_ (s^–1^) × 10^–3^	*k*_cat_/*K*_M_ (μM^–1^ s^–1^) × 10^–3^
PTS_WT_	0.68 ± 0.14	1.16 ± 0.01	1.71
Y525F	4.8 ± 1.26	1.0 ± 0.01	0.21
Y525A	5.27 ± 1.22	0.76 ± 0.07	0.14
Y531A		ND^a^	
Y531F	0.37 ± 0.09	0.57 ± 0.03	1.54
W276A		ND^a^	
C405A	0.67 ± 0.09	1.54 ± 0.04	2.29
PTS_Hα-1_	1.54 ± 0.16	0.85 ± 0.02	0.55
Gd11olS_WT_	0.45 ± 0.08	0.52 ± 0.02	1.16
Gd11olS_Hα-1_	6.02 ± 1.25	0.50 ± 0.05	0.08

a ND: Not determined due to low activity.

We created a homology model using 5-*epi*-aristolochene synthase as template (details in Supporting Information).^5^ We subsequently modeled in (*E*,*E*)-FDP in various possible orientations consistent with C1–C10 cyclization and performed multiple independent molecular dynamics (MD) simulations. By monitoring the C1–C10 distance and propensity to form an *R*-germacryl cation (Figure S2), consistent with patchoulol formation, we predicted the likely PTS:FDP complex (Figure 1A): W276 delineates the pocket at the face opposite to the diphosphate; Y525 and Y531 on the J-K loop (residues 525–537) point into the active site pocket and interact with FDP. Y525 likely coordinates the water molecule(s) involved in hydroxylation, whereas W276 and Y531 ensure a cyclization-competent farnesyl carbocation conformation.

**Figure 1:**
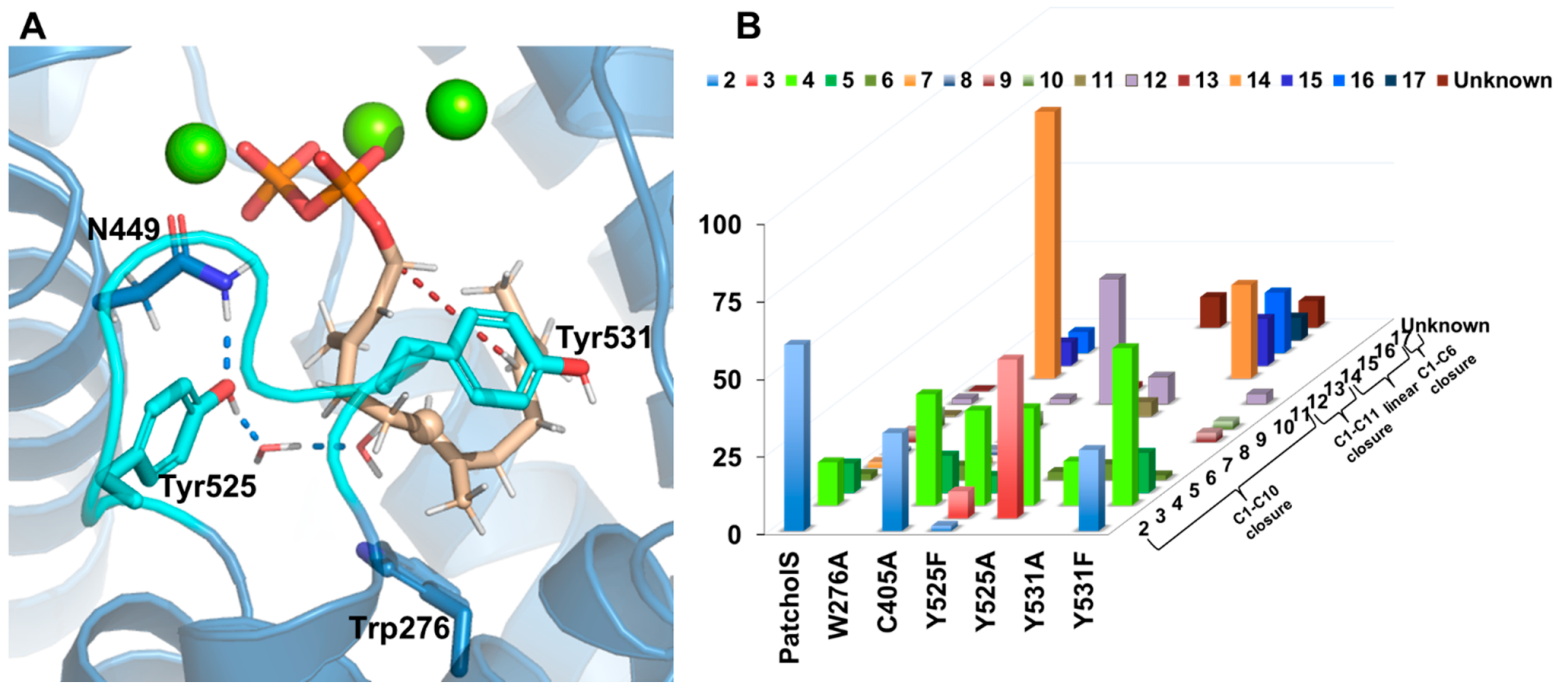
(A) Active site of the predicted PTS:(*E*,*E*)-FDP complex (Mg^2+^ ions colored green; FDP carbon atoms colored “sand” with C6 indicated with a small sphere; J-K loop carbon atoms colored cyan) and key surrounding residues. Selected hydrogen atoms are omitted for clarity. (B) Product profile of PTS_WT_ and variants. Products identified were based on GCMS fragmentation pattern and NIST library search. Blue shades indicate hydroxylated products. Patchoulol (**2**), germacrene A (**3**), α-bulnesene (**4**), α-guaiene (**5**), guai-4-11-diene **(6**), pogostol (**7**), β-patchoulene (**8**), α-patchoulene (**9**), seychellene (**10**), germacrene D (**11**), β-caryophyllene (**12**), α-humulene (**13**), β-farnesene (**14**), nerolidol (**15**), (*E*,*E*)-farnesol (**16**), α-bisabolol (**17**), and unknown sesquiterpenes (**18**–**22**). See also Table S2. TICs are in Figures S4–S20 with mass spectra for all of the compounds in Figures S24–S48.

As expected, functional characterization of variant W276A completely eliminated cyclization, forming 86% β-farnesene (**14**) alongside nerolidol (**15**, 7.6%) and farnesol (**16**, 6.8%). Similarly, variant Y531A produced at least ∼65% linear products (Figure 1B, Table S2). The catalytic activity for W276A and Y531A is severely compromised (Table 1), suggesting their critical role in “folding” the farnesyl chain and possibly aid carbocation stabilization.

MD simulations indicate that the hydroxyl of Y525 is located proximal to the C6 position in FDP and likely helps coordinate water molecule(s) involved in carbocation quenching to form patchoulol. Consistent with this, a Y525F mutation resulted in only trace amounts of **2** (<2%) as hydroxylated product, instead generating sesquiterpene hydrocarbons β-caryophyllene (**12**, 40%) and α-bulnesene (**4**, > 30%, Figure 1B). Formation of **12**, requiring C1–C11 cyclization, was confirmed by coinjection studies (Figure S15). The balance between C1–C10 and C1–C11 cyclization is clearly subtle, consistent with the distances measured in MD simulations of the predicted PTS:FDP complex (Figure S2D). The Y525A variant resulted in only non-hydroxylated products derived from C1–C10 cyclization along with ∼9% of **12**. Tailing in the chromatogram before a sharp peak at the retention time of **4** suggested the presence of germacrene A (**3**).^18^ Separation of these two products proved problematic on an achiral stationary phase hence GC analysis on a chiral stationary phase was employed to separate and quantify **3** and **4**, indicating that **3** is the major product (>51%), with ∼31% of **4 (**Figure S21, figures are less precise than other measurements due to this tailing). Similar GC analysis of the other variants also detected ∼9% **3** from Y525F (Figure S22). Y525A and Y525F have an ∼7-fold increase in *K*_M_ compared to PTS_WT_ with similar *k*_cat_ (Table 1). We hypothesize that this is related to less efficient formation of a productive PTS:FDP:(Mg^2+^)_3_ complex by abolishing the hydrogen bond between Y525 and N449, a key residue for Mg^2+^ coordination. This is consistent with PTS Y525W being inactive (Figure S9). Notably, it is possible that Y525 could reprotonate α-bulnesene (before activating the water for hydroxylation), similar to the suggested role of Y520 in Tobacco 5-*epi*-aristolochene synthase in reprotonation of germacrene A.^5^ Eliminating the Y531 hydroxy (Y531F) leads to a profile similar to that of PTS_WT_, with one significant difference: **4** is now the major product (51%), at the cost of **2** (Figure 1B, Figure S23).

Directed mutagenesis of PTS successfully produced variants that were incapable of hydroxylation. However, such specific targeted mutagenesis for structurally uncharacterized TSs is time-consuming and may not be transferable to other TSs. We previously were successful in significantly reducing hydroxylation by targeting the structurally conserved “kink” region of the G-helix in Gd11olS, with the G188A variant producing 88% of **24**.^19^ The equivalent mutation in PTS (C405A) indeed led to primarily non-hydroxylated products with 36% **4**, without impacting turnover (Table 1). However, formation of **2** (>31%) indicates that this strategy may not generally lead to significant reduction in hydroxylation. We therefore considered modifying the Hα-1 loop that undergoes a significant conformational change in the open-to-closed transition in Class I TSs and was suggested as a target to increase TS product diversity.^20^ In the closed form, this loop shields the reacting carbocation species from bulk water (avoiding premature quenching).^1^

We first attempted changing the Hα-1 loop of Gd11olS, based on the loop sequence in the well-characterized, non-hydroxylating selinadiene synthase (SdS).^21^ We replaced 8 or 4 residues in Gd11olS (^238^VEDEGELS^245^ or ^238^VEDE^241^) with the equivalent residues in SdS (^233^RRGSGYYL^240^ or ^233^RRGS^236^). The 8-residue replacement led to inactivity; however, 4-residue replacement variant Gd11olS_Hα-1_ resulted in significantly reduced hydroxylation (8.3% germacredien-11-ol, **23**). The major product was isolepidozene (**24**, 81%, Figure 2B), the final neutral intermediate in production of **23**,^19^ alongside smaller amounts of germacrene D (**11**, 8.4%) and **3** (2.4%). To understand this reduction in hydroxylation, we performed multiple independent MD simulations of Gd11olS_WT_ and Gd11olS_Hα-1_ in complex with **24** (details in the Supporting Information). In simulations of the Gd11olS_WT_:**24** complex, the closest water molecule to C11 is most commonly positioned within 4 Å (>40% of simulation time). Notably, this water molecule is coordinated by H320 such that it can assist water attack by (transiently) accepting a proton, confirming our previous suggestion (Figure 2C; Gd11olS H320F reduces hydroxylation by ∼50%, in favor of **24**).^19^ In equivalent simulations of Gd11olS_Hα-1_, the closest water molecule is typically much further away (only 8% of simulation time within 4 Å) and dominated by a position deeper in the pocket (Figure 2C). This difference is consistently observed in 5 independent simulations. The change in the Hα-1 loop does not cause a direct structural change in the active site template around H320, but it does lead to a significant reduction in water placed in line for hydroxylation at isolepidozene C11, explaining **24** as the major product. Hα-1 loop replacement thus limits the availability of water that can quench carbocations. When the Hα-1 loop from Gd11olS in SdS is used, production of the neutral intermediate germacrene B (**25**) is increased (66% for ^233^VEDE^236^, 98% for ^233^VEDEGELS^240^, at the cost of catalytic efficiency; Figures S19 and S20, Table S3), but no hydroxylation is introduced, presumably because a hydroxylating water is not available.

**Figure 2:**
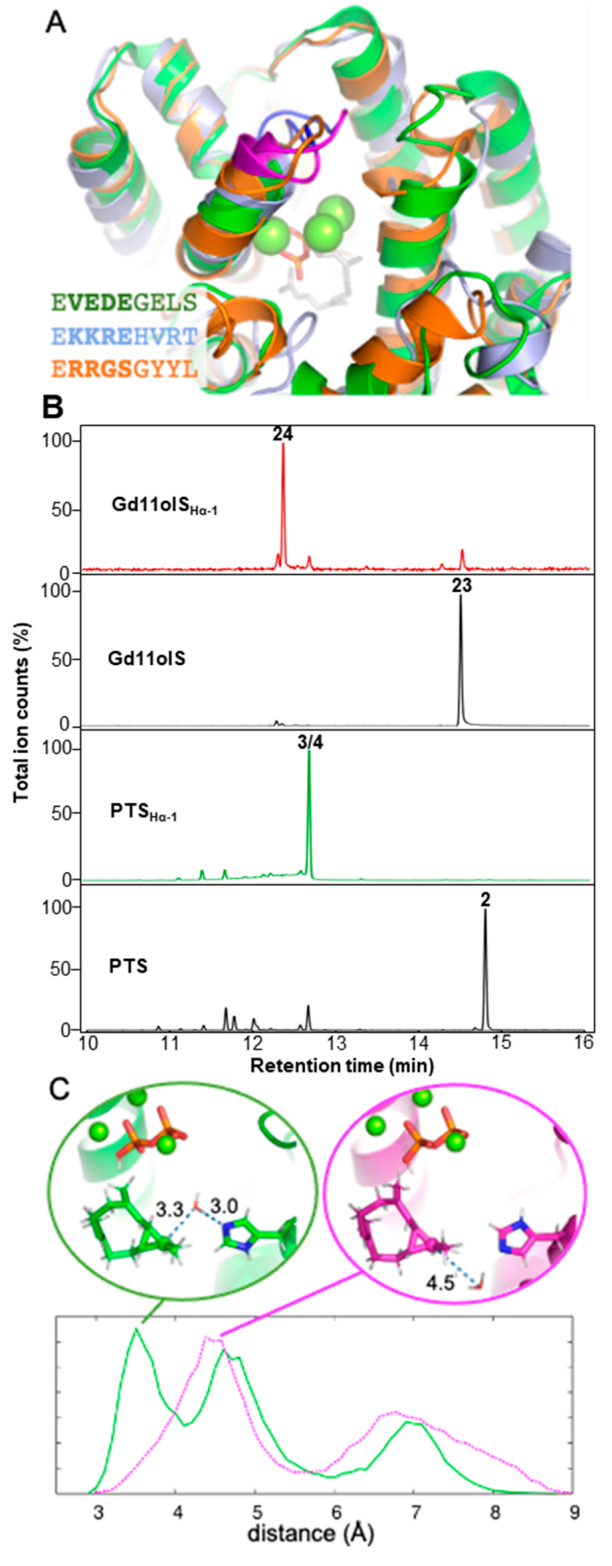
Hα-1 loop variants of Gd11olS and PTS. (A) Closed structures of Gd11olS (PDB 5DZ2, green), PTS (homology model, see the Supporting Information, light blue), and SdS (PDB 4OKZ, orange, with Mg^2+^ as green spheres and FDP with gray carbons), with Hα-1 loop sequences shown. For Gd11olS and PTS, the 4 residues of the Hα-1 loop targeted are colored magenta (and sequence in bold), with the following 4 residues (targeted in the 8-residue variant) blue. (B) TICs of products of Gd11olS and PTS WT and Hα-1 loop variants. (C) Histograms of the distance of the closest water to C11 in isolepidozene (that do not coordinate Mg^2+^), as observed in 5 independent 30 ns simulations of Gd11olS_WT_ and Gd11olS_Hα-1_.

Having established that the four-residue Hα-1 loop variant reduces hydroxylation in Gd11olS, we produced and characterized the equivalent PTS_Hα-1_ variant (^458^KKRE^461^ to RRGS). This resulted in exclusive sesquiterpene hydrocarbon formation, abolishing the formation of **2** (Figure 2B). The major products were **4** (46%) and **3** (40%), both neutral intermediates in the proposed mechanism (Scheme 1). To confidently identify the major products from PTS_Hα-1_, the enzymatic reaction was scaled up (details in the Supporting Information) and the presence of **3**(18,22) and **4**(23) in similar proportions was confirmed by NMR spectroscopy (Figures S58–S62). Several mechanistic proposals for the biosynthesis of patchoulol have been reported.^15,17,24^ A recent detailed labeling study suggested that cyclization of FDP to patchoulol goes through reprotonation of two neutral intermediates (**3** and **4**).^17^ α-Bulnesene (**4**) then undergoes further rearrangement and water capture to form **2**. The product profiles determined here (particularly PTS_WT_, PTS Y531F, and PTS_Hα-1_) support this as the most likely mechanism by indicating that subtle disruption of the active site primarily leads to the formation of these neutral intermediates.

Kinetic characterization of these variants showed increases in *K*_M_ compared to WT (2.5-fold for PTS_Hα-1_ and 15-fold for Gd11olS_Hα-1_), with similar *k*_cat_ values (Table 1). This likely reflects a somewhat hindered open-to-closed conformational change upon formation of the reactive enzyme–FDP complex. Overall, these results suggest that manipulating the Hα-1 loop can avoid water capture in hydroxylating (sesqui)TSs, with little change in initial cyclization propensity and kinetic efficiency.

In conclusion, we have demonstrated a combined computational and experimental approach to manipulate and understand water capture in (sesqui)TSs. Despite a lack of structural information and with limited mutagenic effort, we have obtained PTS Hα-1 and J-K loop variants that produce α-bulnesene, a complex sesquiterpene capable of interfering with platelet aggregation, as the major product. Further, we show that mutagenesis of four amino acids in the structurally conserved Hα-1 loop results in avoiding water capture in two hydroxylating TSs with significantly different loop sequences. Such loop modification may thus be a more general strategy to obtain TS biocatalysts for highly complex terpenes with possible biological applications.

## Data Availability

Starting structures, input files, and analysis data for simulations are available via 10.5281/zenodo.7824977. Raw data for GC-MS analysis, NMR spectroscopic data files and kinetic characterization are available at http://doi.org/10.17035/d.2023.0291278347.

## Supporting Information Available

Cloning, mutagenesis, expression and purification of protein variants, GC/GCMS chromatograms, kinetics and product analysis (including NMR), and details on structure modeling and MD simulation (PDF)

## References

[ref1] Christianson D. W. Structural and Chemical Biology of Terpenoid Cyclases. Chem. Rev. 2017, 117, 11570–11648. 10.1021/acs.chemrev.7b00287. 28841019 PMC5599884

[ref2] Dickschat J. S. Bacterial Diterpene Biosynthesis. Angew. Chemie - Int. Ed. 2019, 58, 15964–15976. 10.1002/anie.201905312. 31183935

[ref3] Miller D. J.; Allemann R. K. Sesquiterpene Synthases: Passive Catalysts or Active Players?. Nat. Prod. Rep. 2012, 29, 60–71. 10.1039/C1NP00060H. 22068697

[ref4] Tholl D. Terpene Synthases and the Regulation, Diversity and Biological Roles of Terpene Metabolism. Current Opinion in Plant Biology. 2006, 9, 297–304. 10.1016/j.pbi.2006.03.014. 16600670

[ref5] Starks C. M.; Back K.; Chappell J.; Noel J. P. Structural Basis for Cyclic Terpene Biosynthesis by Tobacco 5-*Epi*-Aristolochene Synthase. Science. 1997, 277, 1815–1820. 10.1126/science.277.5333.1815. 9295271

[ref6] Wang Y. H.; Xu H.; Zou J.; Chen X. B.; Zhuang Y. Q.; Liu W. L.; Celik E.; Chen G. D.; Hu D.; Gao H.; Wu R.; Sun P. H.; Dickschat J. S. Catalytic Role of Carbonyl Oxygens and Water in Selinadiene Synthase. Nat. Catal. 2022, 5, 128–135. 10.1038/s41929-022-00735-0.

[ref7] Hong Y. J.; Tantillo D. J. Branching out from the Bisabolyl Cation. Unifying Mechanistic Pathways to Barbatene, Bazzanene, Chamigrene, Chamipinene, Cumacrene, Cuprenene, Dunniene, Isobazzanene, Iso-γ-Bisabolene, Isochamigrene, Laurene, Microbiotene, Sesquithujene, Sesquisabinene, T.. J. Am. Chem. Soc. 2014, 136, 2450–2463. 10.1021/ja4106489. 24490652

[ref8] Salmon M.; Laurendon C.; Vardakou M.; Cheema J.; Defernez M.; Green S.; Faraldos J. A.; O’Maille P. E. Emergence of Terpene Cyclization in Artemisia Annua. Nat. Commun. 2015, 6, 6143 10.1038/ncomms7143. 25644758 PMC4327562

[ref9] Hare S. R.; Tantillo D. J. Dynamic Behavior of Rearranging Carbocations - Implications for Terpene Biosynthesis. Beilstein J. Org. Chem. 2016, 12, 377–390. 10.3762/bjoc.12.41. 27340434 PMC4902080

[ref10] Schriever K.; Saenz-Mendez P.; Rudraraju R. S.; Hendrikse N. M.; Hudson E. P.; Biundo A.; Schnell R.; Syrén P. O. Engineering of Ancestors as a Tool to Elucidate Structure, Mechanism, and Specificity of Extant Terpene Cyclase. J. Am. Chem. Soc. 2021, 143, 3794–3807. 10.1021/jacs.0c10214. 33496585 PMC8023661

[ref11] Greenhagen B. T.; Maille P. E. O.; Noel J. P.; Chappell J.; O’Maille P. E. Identifying and Manipulating Structural Determinates Linking Catalytic Specificities in Terpene Synthases. Proc. Natl. Acad. Sci. U. S. A. 2006, 103, 9826–9831. 10.1073/pnas.0601605103. 16785438 PMC1502538

[ref12] Köllner T. G.; Degenhardt J.; Gershenzon J. The Product Specificities of Maize Terpene Synthases Tps4 and Tps10 Are Determined Both by Active Site Amino Acids and Residues Adjacent to the Active Site. Plants 2020, 9, 552 10.3390/plants9050552. 32357450 PMC7284416

[ref13] Driller R.; Janke S.; Fuchs M.; Warner E.; Mhashal A. R.; Major D. T.; Christmann M.; Brück T.; Loll B. Towards a Comprehensive Understanding of the Structural Dynamics of a Bacterial Diterpene Synthase During Catalysis. Nat. Commun. 2018, 9, 3971 10.1038/s41467-018-06325-8. 30266969 PMC6162201

[ref14] Deguerry F.; Pastore L.; Wu S.; Clark A.; Chappell J.; Schalk M. The Diverse Sesquiterpene Profile of Patchouli, *Pogostemon cablin*, Is Correlated with a Limited Number of Sesquiterpene Synthases. Arch. Biochem. Biophys. 2006, 454, 123–136. 10.1016/j.abb.2006.08.006. 16970904

[ref15] Faraldos J. A.; Wu S.; Chappell J.; Coates R. M. Doubly Deuterium-Labeled Patchouli Alcohol from Cyclization of Singly Labeled [2–2H 1]Farnesyl Diphosphate Catalyzed by Recombinant Patchoulol Synthase. J. Am. Chem. Soc. 2010, 132, 2998–3008. 10.1021/ja909251r. 20148554

[ref16] Hsu H. C.; Yang W. C.; Tsai W. J.; Chen C. C.; Huang H. Y.; Tsai Y. C. α-Bulnesene, a Novel PAF Receptor Antagonist Isolated from Pogostemon Cablin. Biochem. Biophys. Res. Commun. 2006, 345, 1033–1038. 10.1016/j.bbrc.2006.05.006. 16712790

[ref17] Xu H.; Goldfuss B.; Schnakenburg G.; Dickschat J. S. The Enzyme Mechanism of Patchoulol Synthase. Beilstein J. Org. Chem. 2022, 18, 13–24. 10.3762/bjoc.18.2. 35047079 PMC8744462

[ref18] Rinkel J.; Dickschat J. S. Addressing the Chemistry of Germacrene A by Isotope Labeling Experiments. Org. Lett. 2019, 21, 2426–2429. 10.1021/acs.orglett.9b00725. 30859837

[ref19] Srivastava P. L.; Escorcia A. M.; Huynh F.; Miller D. J.; Allemann R. K.; Van Der Kamp M. W. Redesigning the Molecular Choreography to Prevent Hydroxylation in Germacradien-11-Ol Synthase Catalysis. ACS Catal. 2021, 11, 1033–1041. 10.1021/acscatal.0c04647. 33614194 PMC7886051

[ref20] López-Gallego F.; Wawrzyn G. T.; Schmidt-Dannert C. Selectivity of Fungal Sesquiterpene Synthases: Role of the Active Site’s H-1α Loop in Catalysis. Appl. Environ. Microbiol. 2010, 76, 7723–7733. 10.1128/AEM.01811-10. 20889795 PMC2988597

[ref21] Baer P.; Rabe P.; Fischer K.; Citron C. A.; Klapschinski T. A.; Groll M.; Dickschat J. S. Induced-Fit Mechanism in Class I Terpene Cyclases. Angew. Chemie Int. Ed. 2014, 53, 7652–7656. 10.1002/anie.201403648. 24890698

[ref22] Faraldos J. A.; Wu S.; Chappell J.; Coates R. M. Conformational Analysis of (+)-Germacrene A by Variable-Temperature NMR and NOE Spectroscopy. Tetrahedron 2007, 63, 7733–7742. 10.1016/j.tet.2007.04.037. 20617157 PMC2898143

[ref23] Rakotonirainy O.; Gaydou E. M.; Faure R.; Bombarda I. Sesquiterpenes from Patchouli (*Pogostemon cablin*) Essential Oil. Assignment of the Proton and Carbon-13 NMR Spectra. J. Essent. Oil Res. 1997, 9, 321–327. 10.1080/10412905.1997.10554251.

[ref24] Croteau R.; Munck S. L.; Akoh C. C.; Fisk H. J.; Satterwhite D. M. Biosynthesis of the Sesquiterpene Patchoulol from Farnesyl Pyrophosphate in Leaf Extracts of *Pogostemon cablin* (Patchouli): Mechanistic Considerations. Arch. Biochem. Biophys. 1987, 256, 56–68. 10.1016/0003-9861(87)90425-5. 3038029

