# Supplementary material for: Active Site Loop Engineering Abolishes Water Capture in Hydroxylating Sesquiterpene Synthases
Source: ACS Catal. 2023 Oct 20;13(21):14199–204. doi: 10.1021/acscatal.3c03920 (PMC10629212; doi:10.1021/acscatal.3c03920)
Supplement: Supplementary file 1 — cs3c03920_si_001.pdf [file cs3c03920_si_001.pdf]

Supporting Information

Active Site Loop Engineering Abolishes Water Capture in Hydroxylating Sesquiterpene Synthases

Prabhakar L. Srivastava,‡,[a] Sam T. Johns,‡,[b] Rebecca Walters,[b] David J. Miller,[a] Marc W. Van der Kamp*[b] and Rudolf K. Allemann*[a]

[a]School of Chemistry, Cardiff University, Main Building, Park Place, Cardiff CF10 3AT, United Kingdom
[b]School of Biochemistry, University of Bristol, University Walk, Bristol BS8 1TD, United Kingdom
‡ Both authors contributed equally

*Correspondences to: marc.vanderkamp@bristol.ac.uk, allemannrk@cardiff.ac.uk

Table of Contents

1.	General materials and methods	S2
2.	Construction of Patchoulol synthase (PTS), Gd11olS and SdS variants	S3
3.	Overproduction and purification of PTS_{WT}, mutants and Hα-1 loop variants	S5
4.	Enzymatic incubation and product analysis	S5
5.	Kinetic characterization of PTS_{WT}, mutants and Hα-1 loop variants	S6
6.	Preparative scale incubations and product characterization	S6
7.	Coinjection studies	S7
8.	Computational methods	S8
9.	Tables	S12
10.	Figures relating to computational simulations	S14
11.	Total ion chromatograms	S18
12.	GC analysis	S29
13.	GCMS Mass Spectra	S31
14.	Kinetic data	S44
15.	NMR spectra	S49
16.	References	S53

1. General materials and methods

The full-length gene sequence (552 aa, Q49SP3) for patchoulol synthase (PTS) from *Pogostemon cablin*[1] was codon optimised for *E. coli* expression and synthesized in a pET28a vector frame between EcoR1 and HindIII restriction site from Genscript with an N-terminal His_6 tag.

The N-terminal Geosmin/Germacrandien-11-ol synthase (Gd11olS, 1-366 aa of whole protein, Q9X839) from *Streptomyces coelicolor*[2,3] was codon optimised for *E. coli* expression and synthesized in a pET28a vector frame between EcoR1 and HindIII restriction site from Genscript with an N-terminal His_6 tag.

The full-length gene sequence (365 aa) for selina-4(15),7(11)-diene synthase (SdS, 4OKM) from *Streptomyces pristinaespiralis*[4] was codon optimised for *E. coli* expression and synthesized in a pET28a vector frame between EcoR1 and HindIII restriction site from Genscript with an N-terminal His_6 tag.

An unstained protein size marker (14.4-116.0) kDa was used to identify proteins by 12 % SDS-PAGE. PrimeSTAR® master mix was purchased from TaKaRa. Primers for mutagenesis were purchased from Sigma Aldrich (UK). All mutated constructs were confirmed by DNA sequence analysis from Eurofins. [1-^{3}H]-FDP (20 Ci/mmol) was purchased from American Radiolabelled chemicals, Inc. Commercial [1-^{3}H]-FDP was diluted by adding unlabelled (*E*,*E*)-FDP to give a final specific activity of 24000 dpm/µM. ^{1}H and ^{13}C NMR spectra were measured on a Bruker Ultrashield 500 NMR spectrometer and are reported as chemical shifts in parts per million downfield from tetramethylsilane (^{1}H and ^{13}C).

(*E*,*E*)-FDP was synthesized at Cardiff following the procedure as described previously.[5]

Gas chromatography coupled with mass spectrometry (GC-MS) was performed on a Perkin Elmer Clarus 680 GC fitted with a Perkin Elmer Elite-1 column 100 % dimethyl polysiloxane (30 m x 0.25 mm internal diameter) and a Perkin Elmer Clarus SQ 8 C mass spectrometer. Method 1: The elution program used an injection port temperature of 100°C; split ratio 19:1; initial temperature 80°C hold 2 min, ramp of 8°C/min to 280°C and hold for 3 min with a flow rate of 1 mL He/min. GC analysis on a chiral stationary phase to separate α-bulnesene and germacrene A was performed on Agilent GC using cyclodextrin column (30 m x 0.25 mm internal diameter) on following parameter: Method 2: injection port 100°C; split ratio 5:1; initial pressure 1 kPa; initial temperature 80°C, hold 2 min, ramp of 8°C/min to 220°C (3 min hold), flow

1 mL H/min. All the GCMS injections were performed on the Perkin Elmer Clarus 680 GC using GC method 1 unless specified.

2. Construction of Patchoulol synthase (PTS), Gd11olS and SdS variants

Mutation primers were designed for the selected amino acids present in the active site pocket (Table S1). For PCR amplification, the following mixture was used: 12.5 µL PrimeSTAR® master mix, 0.5 µL forward/ reverse primer (10 µM stock), 50 ng template DNA (plasmid), 1 µL DMSO and water to a final volume of 25 µL. After the PCR, 1 µL of DpnI (NEB) was added to each reaction and the mixture was incubated at 37°C for 2 h to digest the template DNA. 5 µL of Dpn1-treated samples were transformed into XL1 blue chemical competent cells, plated on LB kanamycin agar plates and incubated overnight at 37°C. Single colonies were inoculated in LB medium containing kanamycin (50 µg/mL), incubated overnight at 37°C at 200 rpm and used for plasmid isolation using a plasmid miniprep kit (Qiagen). The mutant sequences were verified via sequencing using the forward (T7 promoter) or the reverse (T7 terminator) primers.

For the preparation of germacradien-11-ol synthase Hα-1 variants (Gd11olS$_{Hα-1}$), to replace either 8 or 4 residues in Gd11olS (^{238}VEDEGELS^{245} or ^{238}VEDE^{241}) with the equivalent residues from selinadiene synthase (SdS, ^{233}RRGSGYYL^{240} or ^{233}RRGS^{236}), initially PCR amplification of Gd11olS was performed using Gd11olS$_{Hα-1}$ variant$_{238-245}$ forward and reverse primers containing overhang region to create the variant containing 8 amino acid (^{233}RRGSGYYL^{240}) replacement. The PCR amplicon was purified from agarose gel using gel extraction method and mixed with SdS$_{Hα-1}$ forward and reverse primers containing the overlapping sequence from Gd11olS and ligated using NEBuilder® Hifi DNA assembly master mix as per manufacturer's instruction (NEB). Ligation mixture was transformed in XL-1 blue chemical competent cells, plated on LB kanamycin agar plates and incubated overnight at 37 °C. Plasmid isolation was performed from positive clones and sequenced with T7 terminator primer to verify the presence of mutant sequence. To create Gd11olS$_{Hα-1}$ (^{238}VEDE^{241} replaced by corresponding sequence of ^{233}RRGS^{236} from SdS), PCR amplification was performed using Gd11olS$_{Hα-1}$ variant$_{238-241}$ forward and reverse primers and Gd11olS$_{Hα-1}$ variant (containing 8 amino acid) as template. Amplified PCR product

was purified from the agarose gel and quantified using Nanodrop (ThermoFisher). Purified PCR amplicon was ligated using KLD Enzyme Mix from NEB as per manufacturer's instruction. Ligated mixture was transformed in XL-1 blue cells and positive clones were used to isolate plasmids. Plasmids were sequenced to confirm the presence of mutation. For preparation of $PTS_{H\alpha\text{-}1}$ variants, PCR amplification was performed using $PTS_{H\alpha\text{-}1}$ variant forward and reverse primers to generate PCR amplicons containing overhang regions to replace ^{458}KKRE^{461} with corresponding sequence from selinadiene synthase (SdS) ^{233}RRGS^{236}. PCR product was purified from agarose gel using gel extraction procedure, quantified by Nanodrop and ligated using KLD Enzyme Mix (NEB) as per manufacturer instruction. Ligated mixtures were transformed in XL-1 blue chemical competent cells, plated on LB kanamycin agar plates and incubated at 37°C for overnight. Positive clones were used for plasmid isolation and sequenced using T7 terminator primer to verify the presence of mutant sequence.

For the creation of $SdS_{H\alpha\text{-}1}$ variant, to replace either 8 or 4 residues in SdS, ^{233}RRGSGYYL^{240} or ^{233}RRGS^{236} by equivalent redisues in Gd11olS ^{238}VEDEGELS^{245} or ^{238}VEDE^{241}, initial PCR amplification was performed using overhang $SdS_{H\alpha\text{-}1}$ variant$_{233\text{-}236}$_forward and reverse primers (Table S1) to generate 4-residue variant (SdS, ^{233}RRGS^{236} replaced by Gd11olS ^{238}VEDE^{241}). The PCR amplicon was purified from agarose gel using gel extraction method and self-ligated using NEBuilder® Hifi DNA assembly master mix as per manufacturer's instruction (NEB). Ligation mixture was transformed in XL-1 blue chemical competent cells, plated on LB kanamycin agar plates and incubated overnight at 37°C. Plasmid isolation was performed from positive clones and sequenced with T7 terminator primer to verify the presence of mutant sequence. After confirming the sequence, this variant was used as template to generate the $SdS_{H\alpha\text{-}1}$ variant with 8-residue replacement (SdS, ^{233}RRGSGYYL^{240} replaced by equivalent residue in Gd11olS ^{238}VEDEGELS^{245}), PCR amplification was performed using $SdS_{H\alpha\text{-}1}$ variant$_{238\text{-}240}$ forward and reverse primer. The PCR amplicon was purified from agarose gel using gel extraction method and self-ligated using NEBuilder® Hifi DNA assembly master mix as per manufacturer's instruction (NEB). Ligation mixture was transformed in XL-1 blue chemical competent cells, plated on LB

kanamycin agar plates and incubated overnight at 37°C. Plasmid isolation was performed from positive clones and sequenced with T7 terminator primer to verify the presence of mutant sequence.

3. Overproduction and purification of PTS_{WT}, mutants and Hα-1 loop variants

pET28a harbouring patchoulol synthase wild-type (PTS_{WT}) and mutants, germacradien-11-ol synthase wild-type ($Gd11olS_{WT}$), $Gd11olS_{H\alpha\text{-}1}$ loop variants and Selina-diene synthase (SdS) and $SdS_{H\alpha\text{-}1}$ loop variants were introduced in BL21(DE3) chemically competent cells for protein expression and positive clones were selected on LA + kanamycin plates. A single colony was used to inoculate 20 mL of LB media containing 50 µg/mL of kanamycin and grown overnight at 37ºC and 200 rpm. The resulting culture was transferred to 500 mL of terrific broth (TB) media containing 50 µg/mL of kanamycin and grown at 37ºC and 200 rpm until the OD at 600 nm reached 0.8 - 1.0. At this stage, cultures were induced with a final concentration of 0.5 mM isopropyl β-D-1-thiogalactopyranoside (IPTG) and incubated at 16°C and 200 rpm for 6hr. After induction, cells were harvested by centrifugation at 5000*g* for 20 min and pellets were stored in 20°C until further use. For the purification of protein, pellets were resuspended in 20 mL lysis buffer /g of cell mass (50 mM Tris, pH 8.0, 500 mM NaCl, 10 mM $MgCl_2$, 10 % glycerol, 1 mg/mL lysozyme, and 1 mM PMSF). The cells were lysed by sonication (amplitude 40 %, pulse on 5 s and pulse off 10 s for 5 min) and the resulting supernatant was centrifuged at 18000*g* for 45 min to remove the cell debris. The supernatant was filtered through 0.2 µm syringe filters and applied to a Ni-NTA affinity drip column (QIAGEN, 5 mL). Proteins were eluted with 2 column volumes of 40 - 250 mM imidazole gradient in lysis buffer. Fractions containing proteins with purity >95 % as judged by SDS-PAGE were combined and dialyzed against 20 mM Tris, pH 8.0, 100 mM NaCl, glycerol 10 %. The Bradford protein assay[6] was used to measure the concentration of total protein and commercial bovine serum albumin as the calibration standard.

4. Enzymatic incubations and product analysis

In order to functionally characterize the purified proteins (PTS_{WT}, PTS mutants, $PTS_{H\alpha\text{-}1}$ loop variant, $Gd11olS_{WT}$, $Gd11olS_{H\alpha\text{-}1}$ loop variant and SdS_{WT} and $SdS_{H\alpha\text{-}1}$ loop variant), enzymatic assays were setup using 10 µM of purified protein in 50 mM Tris buffer, pH 8.0, containing 5 mM $MgCl_2$, 5.0 mM 2-

mercaptoethanol and 100 μM FDP in a 500 μL scale overlaid with 1 mL of n-pentane, incubated for overnight at room temperature with gentle agitation. After the incubation, assay mixtures were vortexed, and the n-pentane layer was transferred to fresh vial. The pentane extracts were analyzed by GC-MS using the method described above. The products formed were verified by coinjection with authentic standard samples and NIST Library Mass Spectra matches.

5. Kinetic characterization of PTS_{WT}, mutants and Hα-1 loop variants

Steady-state kinetics assays were carried out using [1-^{3}H]-(*E*,*E*)-FDP (240000 dpm $nmol^{-1}$) in a similar way as previously described.[7] Briefly, reactions (final volume of 250 μL) were initiated by addition of enzyme (100 nM final concentration) to assay buffer solutions containing [1-^{3}H]-(*E*,*E*)-FDP (0.02-15 μM) at 0°C overlaid with 1 mL of hexane. The resulting enzymatic preparations were incubated at 30°C for 10 minutes. After reaction, samples were transferred on ice and immediately quenched by addition of EDTA (50 μL, 0.5 M) and vortexing for 30 s. The organic layer was then passed through a small silica column (~500 mg) into 15 mL EcoScintTM fluid (National Diagnostics), the aqueous portion was further extracted two times with 1 mL portions of 11:1 hexane/diethyl ether by vortexing for 10 s and the organic extracts were passed through the same silica column into the 15 mL EcoScintTM fluid. At the end, the silica column was washed with 1 mL of 11:1 hexane/diethyl ether. The combined organic extracts in EcoScintTM fluid was analysed on a scintillation counter (Packard 2500 TR^{TM}) in ^{3}H mode for 4 min per sample. The kinetic constants (K_M and k_{cat}) were calculated by fitting the data to the Michaelis-Menten equation, using Systat Sigmaplot.

6. Preparative scale incubations and product characterization

For the characterization of major metabolites from $PTS_{H\alpha\text{-}1}$ loop variant, preparative scale incubations were carried out using 10 μM of purified protein with 0.35 mM FDP in Tris-HCl buffer, pH 8.0 containing, 5 mM $MgCl_2$ and 5 mM 2-mercaptoethanol in a total reaction volume of 200 mL. This solution was overlaid with 200 mL of pentane and incubated at room temperature for 24 h with gentle stirring. Reactions were performed in duplicates. After incubation, both the reaction mixtures were pooled, extracted twice with 200mL n-pentane each time, and the pentane solution was analysed by GC-

MS. The resulting solution was filtered, and solvent removed carefully under reduced pressure (500 mbar, 30°C water bath) to give 14.5 mg of colourless oil as judged by taking weight using analytical balance. For NMR spectroscopic analysis the sample was dissolved in $CDCl_3$ and analysed by ^{1}H, ^{13}C and DEPT NMR spectroscopy. The structures of the metabolites: germacrene A (**3**), α-bulnesene (**4**) were confirmed by comparing with the reported NMR spectra.[8–10]

7. Coinjection study

Formation of β-caryophyllene (**11**) by PTS Y525F was confirmed by coinjection of Y525F assay mixture with authentic sample generated from mutant (G402C) of germacrene A synthase from *Solidago canadensis* which has been shown to produce a small quantity of β-caryophyllene (**11**).[11] The coinjection chromatograms are shown below (**Figure S15**). We also carried out coinjection of $PTS_{H\alpha\text{-}1}$ variant with *Streptomyces coelicolor* germacradien-11-ol synthase (Gd11olS) mutant W312F (shown to make germacrene A as major product),[12] for the characterization of germacrene A (**3**) coeluting with α-bulnesene in GCMS (**Figure S16**).

8. Computational Methods

Homology modelling PTS

In the absence of an experimental structure for patchoulol synthase (PTS), we proceeded to build a homology model that should represent the active closed conformation. Two templates were initially considered: δ-cadinene synthase in complex with 2-fluoro-FDP (45 % sequence identity to PTS, PDB: 3G4F) and 5-*epi* aristolochene synthase in complex with FDP (40 % sequence identity, PDB: 5IK0).[13] The former is, however, not representative of an active closed Class I terpene synthase conformation: the farnesyl chain protrudes out of the cavity and the capping loops are disordered/undefined. We thus proceeded to build a model with 5IK0 as the template, employing MODELLER v. 9.20.[14] FDP and the three Mg^{2+} ions were included in the model building as 'generic' residues (guess_atom_types used for approximate parameters). 5 models were generated using MODELLER's automodel function, with thorough VTFM (autosched.slow) and MD (refine.very_slow) optimization. For completeness, the alignment file used (obtained using Clustal Omega, leaving out the first 13 residues that are not observed in the crystal structure) and the MODELLER input script and are included below. One model had both the highest MODELLER DOPE and (standard) molpdf scores. Visual inspection verified that this model provided an active site cavity with the expected Mg^{2+}/diphosphate coordination, and active site residues had similar side-chain rotamers as in the template (**Figure S1A**).

Alignment file "ali"

```
>P1;5ik0
structureX:5ik0:13:A:+540:A:::-1.00:-1.00
IVRPVADFSPSLWGDQFLSFSIKNQVAEKYAKEIEALKEQTRNMLLAT
GMKLADTLNLIDTIERLGISYHFEKEIDDILDQIYNQ----NSNCNDLCTSALQFRLLRQ
HGFNISPEIFSKFQDENGKFKESLASDVLGLLNLYEASHVRTHADDILEDALAFSTIHLE
SAAPHLKSPLREQVTHALEQ-CLHKGVPRVETRFFISSIYDKEQSKNNVLLRFAKLDFNL
LQMLHKQELAQVSRWWKDLDFVTTLPYARDRVVECYFWALGVYFEPQYSQARVMLVKTIS
MISIVDDTFDAYGTVKELEAYTDAIQRWDINEIDRLPDYMKISYKAILDLYKDYEKELSS
AGRSHIVCHAIERMKEVVRNYNVESTWFIEGYTPPVSEYLSNALATTTYYYLATTSYLGM
KS--ATEQDFEWLSKNPKILEASVIICRVIDDTATYEVEKSRGQIATGIECCMRDYGIST
KEAMAKFQNMAETAWKDINEGLLRPTPVSTEFLTPILNLARIVEVTYIHNLDGYTHPEKV
LKPHIINLLVDSIKI....*

>P1;patch_synth
sequence:::::::::
ASRPLANFHPCVWGDKFIVYNPQSCQAGER-EEAEELKVELKRELKEA
```

SDNYMRQLKMVDAIQRLGIDYLFVEDVDEALKNLFEMFDAFCKNNHDMHATALSFRLLRQ
HGYRVSCEVFEKFKDGKDGFKVPNEDGAVAVLEFFEATHLRVHGEDVLDNAFDFTRNYLE
SVYATLNDPTAKQVHNALNEFSFRRGLPRVEARKYIS-IYEQYASHHKGLLKLAKLDFNL
VQALHRRELSEDSRWWKTLQVPTKLSFVRDRLVESYFWASGSYFEPNYSVARMILAKGLA
VLSLMDDVYDAYGTFEELQMFTDAIERWDASCLDKLPDYMKIVYKALLDVFEEVDEELIK
LGAPYRAYYGKEAMKYAARAYMEEAQWREQKHKPTTKEYMKLATKTCGYITLIILSCLGV
EEGIVTKEAFDWVFSRPPFIEATLIIARLVNDITGHEFEKKREHVRTAVECYMEEHKVGK
QEVVSEFYNQMESAWKDINEGFLRPVEFPIPLLYLILNSVRTLEVIYKE-GDSYTHVGPA
MQNIIKQLYLHPVPY....*

MODELLER input script

```
from modeller import *
from modeller.automodel import *    # Load the automodel class
from modeller.scripts import complete_pdb, cispeptide

env = environ()

# directories for input atom files – here 5ik0.pdb
env.io.atom_files_directory = ['.', '../']
env.io.hetatm = True

class MyModel(automodel):
        def special_patches(self, aln):
                self.guess_atom_types()

a = automodel(env, alnfile = 'ali',
            knowns = ('5ik0'), sequence = 'patch_synth', assess_methods=assess.DOPE)

a.very_fast()
a.starting_model= 1
a.ending_model  = 5
a.final_malign3d = True

# Very thorough VTFM optimization:
a.library_schedule = autosched.slow
a.max_var_iterations = 300

# Thorough MD optimization:
a.md_level = refine.very_slow

a.make()
```

MD simulations of PTS:FDP complexes

Starting from the PTS homology model and the Mg^{2+} and diphosphate ions transferred from the template (PDB 5IK0), the farnesyl chain was built in manually (using PyMOL), such that no significant clashes occurred with the residues protruding into the active site cavity and the farnesyl chain was in a cyclisation competent conformation (dC1-C10 < 5 Å). This resulted in three conformations with the farnesyl attached

to the PA phosphate oxygen (to which the farnesyl is bound in 5IK0) and two conformations attached to the alternative PB phosphate oxygen (**Figure S1B,C**). For each of these five, the final part of the farnesyl chain was then rotated such that diphosphate-farnesyl ionization and C1-C10 cyclisation would lead to either an R- or S-germacryl cation. The resulting 10 starting structures were prepared for simulation identically. Protonation states predicted with PropKa3.1[15,16] indicated that all residues were in their standard states, with Glu/Asp negatively charged, Lys/Arg positively charged, and His neutral. The reduce utility (from AmberTools18) was then used to flip His/Asn/Gln residues to optimize the hydrogen bonding network, and to determine the His tautomers (His 105, 108, 158, 162, 194, 225, 243, 390, 474, 533 and 548 protonated on NE2, His 21, 120, 224, 454, 462 protonated on ND1). Initial water solvation was performed through 3D-RISM and Placevent. For 3D-RISM calculations (performed with AmberTools18), the Kovalenko-Hirata (KH) closure method[17,18] was used, with all other settings kept as default. Placevent[19] was then used to solvate the solute with water molecules up to 5 Å away from any protein atom. The systems were then fully solvated using CHARMM-GUI v3.5 (http://www.charmm-gui.org) to have at least 10 Å between any protein and Placevent placed atoms and the edge of the rectangular simulation box, and neutralized by adding 13 Na^+ ions. The protein was treated using the CHARMM36 force-field,[20] with water as the CHARMM-adapted TIP3P model and standard CHARMM ion parameters.[21] FDP were taken from Van der Kamp et al.

Subsequently, the Amber18 program was used to perform an MD protocol identical to that described previously,[12] performing 4 independent simulations for each of the 10 FDP starting conformations. In short, the preparation consisted of minimisation of hydrogens, then all solvent molecules and then all atoms with positional restraints of 5 kcal·mol^{-1}·Å^{-2} applied to the Cα atoms and the Mg^{2+}B ion. These positional restraints were maintained during heating to 300 K (in 60 ps, NVT ensemble) and equilibration of pressure (300 K and 1 bar, NPT ensemble, 100 ps). Positional restraints were then released gradually in further 50 ps NPT ensemble simulations. Finally, 30 ns MD production simulations were performed. Throughout all simulations, distance restraints were applied to maintain coordination between diphosphate oxygens and the Mg^{2+}A,C ions (one-sided harmonic restraint of 50

kcal·mol^{-1}·Å^{-2} for distances >2.2 Å). Four independent simulations (from different initial velocities) were run for each of the 10 farnesyl starting positions. For two selected conformations from these initial simulations, the minimization and simulation protocols were repeated (see below). Throughout, periodic boundary conditions and a time step of 2 fs were used, with a direct space cut-off of 8 Å for nonbonded interactions with PME for long-range electrostatics. All bonds involving hydrogen atoms were constrained by SHAKE.

Modelling and MD simulation of Gd11olS complexes with isolepidozene

For the simulation of Gd11olS$_{WT}$ with isolepidozene, protein coordinates and protonation states were taken from our previous work.[12] For the Gd11olS$_{H\alpha-1}$ variant, new coordinates for the full 8-residue stretch, with the first 4 residues replaced by those in SdS (resulting in ^{238}RRGSGELSN^{246}), were obtained using MODELLER v 9.20.[14] 200 different loop models were generated. Top models according to both DOPE and molpdf score were investigated in detail, and a model with top DOPE score (recommended score for loop modelling) was selected for simulation (Figure S3A). Simulations with FDP as performed previously for Gd11olS$_{WT}$ and several point mutants was performed,[12] to ensure that the Gd11olS$_{H\alpha-1}$ variant likely prefers the same FDP conformation as Gd11olS$_{WT}$ (based on predominance of pre-R poses). Subsequently, isolepidozene (M06-2X/TZVP optimized structure with the PubChem [https://pubchem.ncbi.nlm.nih.gov/] database structure as starting point) was modelled in the active site of Gd11olS$_{WT}$ and Gd11olS$_{H\alpha-1}$ based on this FDP conformation (**Figure S3**). Simulations were then set up using the CHARMM-GUI as described above (CHARMM36 protein force field, with parameters for PPi from the nucleic acid library), with diphosphate singly protonated on the oxygen in position for having accepted the proton, and using CGenFF[22] for isolepidozene. MD simulation was then performed as before for PTS (see above). The distance was measured between C11 and the oxygen of the closest water molecule, once all waters coordinating to Mg^{2+} ions are excluded (based on water-O to Mg^{2+} distance <=2.5 Å).

9. Tables

Table S1: Primer Sequence for mutation

**Primer name**	**Primer sequence (5'-3')**
W276A Forward	CTATTTT**GCG**GCGAGCGGTAGCTACTTCGAACCG
W276A Reverse	GCTCGC**CGC**AAAATAGCTCTCAACCAGACGGTCACGC
C405A Forward	CAAAACC**GCC**GGTTATATCACCCTGATCATTCTGAGC
C405A Reverse	ATAACC**GGC**GGTTTTGGTCGCCAGCTTCATGTATTC
Y525F Forward	GTGATC**TTC**AAGGAAGGTGATAGCTATACCCACGTGG
Y525F Reverse	TTCCTT**GAA**GATCACTTCCAGGGTACGCACGCTG
Y525A Forward	GTGATC**GCC**AAGGAAGGTGATAGCTATACCCACGTGG
Y525A Reverse	TTCCTT**GGC**GATCACTTCCAGGGTACGCACGCTG
Y531A Forward	GATAGC**GCT**ACCCACGTGGGCCCGGCGATGCAGAAC
Y531A Reverse	GTGGGT**AGC**GCTATCACCTTCCTTGTAGATCACTTCC
Y531F Forward	GATAGC**TTT**ACCCACGTGGGCCCGGCGATGCAGAAC
Y531F Reverse	GTGGGT**AAA**GCTATCACCTTCCTTGTAGATCACTTCC
Y525W Forward	GTGATC**TGG**AAGGAAGGTGATAGCTATACCCACGTGG
Y525W Reverse	TTCCTT**CCA**GATCACTTCCAGGGTACGCACGCTG
PTS$_{H\alpha\text{-}1}$ variant_Forward:	**GGTAGC**CACGTGCGTACCGCGGTTGAATGC
PTS$_{H\alpha\text{-}1}$ variant_Reverse:	**ACGACG**TTCGAACTCGTGGCCGGTAATATCG
Gd11olS$_{H\alpha\text{-}1}$ variant$_{238\text{-}245}$_Forward:	**GGCTACTATCTG**AACGGCGTGCTGGTTCTGGAGACC
Gd11olS$_{H\alpha\text{-}1}$ variant$_{238\text{-}245}$_Reverse:	**GCTACCACGACG**CTCACGTTGGTAGCTGAACAGGTCG
SdS_H$_{\alpha 1(233\text{-}240)}$_Forward:	**CGTCGTGGTAGCGGCTACTATCTG**
SdS_H$_{\alpha 1(233\text{-}240)}$_Reverse:	**CAGATAGTAGCCGCTACCACGACG**
Gd11olS$_{H\alpha\text{-}1}$ variant$_{238\text{-}241}$_Forward:	**GAACTGAGC**AACGGCGTGCTGGTTCTGGAGACC
Gd11olS$_{H\alpha\text{-}1}$ variant$_{238\text{-}241}$_Forward:	GCCGCTACCACGACGCTCACGTTGG
SdS$_{H\alpha\text{-}1}$ variant$_{233\text{-}236}$_Forward:	AAGGAA**GTTGAAGACGAG**GGCTACTATCTGAACGCGCTG
SdS$_{H\alpha\text{-}1}$ variant$_{233\text{-}236}$_Reverse:	GTAGCC**CTCGTCTTCAAC**TTCCTTGTGATAGCTAAAG
SdS$_{H\alpha\text{-}1}$ variant$_{238\text{-}240}$_Forward:	**GAACTGAGC**AACGCGCTGCGTGTTCTGG
SdS$_{H\alpha\text{-}1}$ variant$_{238\text{-}240}$_Reverse:	GTT**GCTCAGTTC**GCCCTCGTCTTCAACTTCC

Table S2: Product distributions in the pentane extracts, arising from incubation of (*E*,*E*)-FDP (**1**) with PTS_{WT} and mutants. **2**: patchoulol, **3**: germacrene A, **4**: α-bulnesene, **5**: α-guaiene, **6**: guai-4,11-diene, **7**: pogostol, **8**: β-patchoulene, **9**: α-patchoulene, **10**: seychellene, **11**: germacrene D, **12**: β-caryophyllene, **13**: α-humulene, **14**: β-farnesene, **15**: (*E*)-nerolidol, **16**: (*E*,*E*)-farnesol, **17**: α-bisabolol, **18-22**: unknown sesquiterpenes. Tabulated data are % of total terpene production by each variant. We compared the mass fragmentation pattern with the earlier reports on PcPTS to identify the minor metabolites. However, for the major metabolites, we have compared it with the authentic standards (β-farnesene, caryophyllene). Germacrene A and α-bulnesene has been characterised by NMR analysis.

	**2**	**3**	**4**	**5**	**6**	**7**	**8**	**9**	**10**	**11**	**12**	**13**	**14**	**15**	**16**	**17**	**18**	**19**	**20**	**21**	**22**
**PTS_{WT}**	60.0		13.9	9.5	2.0	1.8	1.2	3.6	5.7	0.5	1.7	0.3									
**W276A**													85.7	7.6	6.8						
**C405A**	31.6		35.9	12.0	4.7	1.7	1.6	6.5	4.3		1.7										
**Y525F**	1.7	8.6	30.6	5.5	1.9						40.3	1.6					2.8		4.9	2.2	
**Y525A**		51.1	31.3		2.6					4.6	8.7						1.6				
**Y531A**			14.2		5.1								30.1	15.1	19.5	7.3		4.1			4.5
**Y531F**	26.1		50.7	12.9	1.5			3.0	2.5		3.3										
**Y525W**																					
**$PTS_{H\alpha\text{-}1}$**		40.0	46.3	5.3	2.2						6.2										

Table S3: Kinetic constants of $SdS_{H\alpha\text{-}1}$ variants from three technical replicates.

	K_M (μM)	k_{cat} (s^{-1}) x 10^{-3}	k_{cat}/K_M (μM^{-1} s^{-1}) x 10^{-3}
SdS_{WT}	0.86 ± 0.11	7.0 ± 0.02	8.14
$SdS_{H\alpha\text{-}1\ \text{variant (233-236)}}$	7.34 ± 1.43	4.0 ± 0.04	0.55
$SdS_{H\alpha\text{-}1\ \text{variant (233-240)}}$	7.02 ± 1.52	0.45 ± 0.004	0.06

10. Figures relating to computational simulation

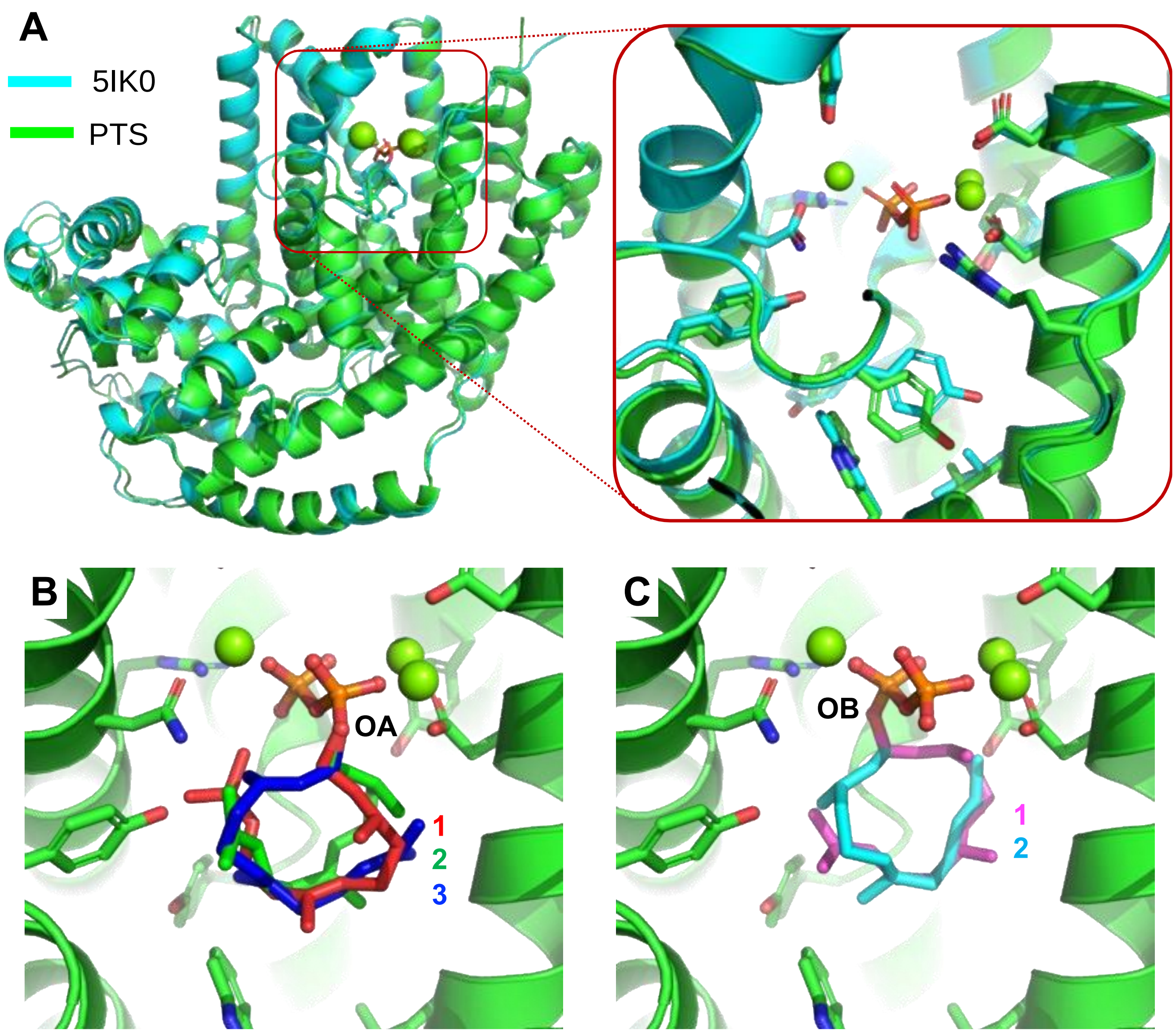

Figure S1. Structural models of PTS complexed with FDP. **A**) Homology model of PTS (green carbons) aligned to its template, PDB 5IK0 (cyan carbons). The insert highlights the diphosphate, Mg^{2+} ions and the side chains around the active site. **B**) Farnesyl starting orientations with C1 attached to diphosphate OA (corresponding to atom O1 in PDB 5IK0). **C**) Farnesyl starting orientations with C1 attached to diphosphate OB (corresponding to atom O2B in PDB 5IK0). Hydrogens omitted for clarity. Farnesyl carbon colouring consistent with line colours in **Figure S2A,B**.

Additional Computational Results

Establishing the most likely farnesyl binding mode in PTS

Using the homology model of PTS, MD simulations were performed with five different initial starting positions of the farnesyl chain (**Figure S1B,C**), three attached to the diphosphate OA (OA-1, -2, and -3) and two to OB (OB-1, OB-2). For each, the final portion of the chain was oriented in line with pre-*R*

(attack from the *Si* face at C10, leading in an *R* conformation at C10) and pre-*S* (attack from the *Re* face at C10, leading to an *S* conformation at C10) conformations for C1-C10 cyclization (resulting to 10 starting positions). The simulations for each of the 5 alternative farnesyl positions (240 ns for each, from 2x (pre-*R*/*S*) 4x independent 30 ns production simulations) were analysed for consistency with C1-C10 cyclization (C1-C10 distance < 5 Å) leading to the expected *R*-germacryl cation (dihedral angle –125° ≤ C10-C11-H10-C1 ≤ –55°). Only farnesyl starting positions OA-3 and (to a lesser extent) OB-2 lead to substantial sampling of both the distance and stereoselectivity (dihedral angle) requirements (**Figure S2A,B**). Visual inspection indicated that in the case of OB-2, conformations with C1-C10 distance < 5 Å were typically observed when the diphosphate-Mg^{2+} coordination was significantly altered. The diphosphate and Mg^{2+} ions had moved 'upwards' compared to their positions observed in X-ray crystal structures of sesquiterpene synthases with FDP analogues (and OA-3 simulations), including a loss of interaction between Mg^{2+}_B and Asn450 from the binding motif. Therefore, OA-3 (after relaxation through MD simulation) is the most probable farnesyl conformation involved in formation of patchoulol.

To obtain additional insights in the probable PTS-FDP complex (OA-3 farnesyl conformation), and confirmation of its likelihood, further simulations were performed using two energy minimized snapshots from the OA-3 simulations (where *d*C1-C10 < 5 Å and dihedral angle –125° ≤ C10-C11-H10-C1 ≤ –55°). With these two new starting conformations, the simulations, *d*C1-C10 (and *d*C1-C11) was below < 5 Å for the majority of the simulation time (**Figure S2D**), and frequently in the expected pre-*R* conformation.

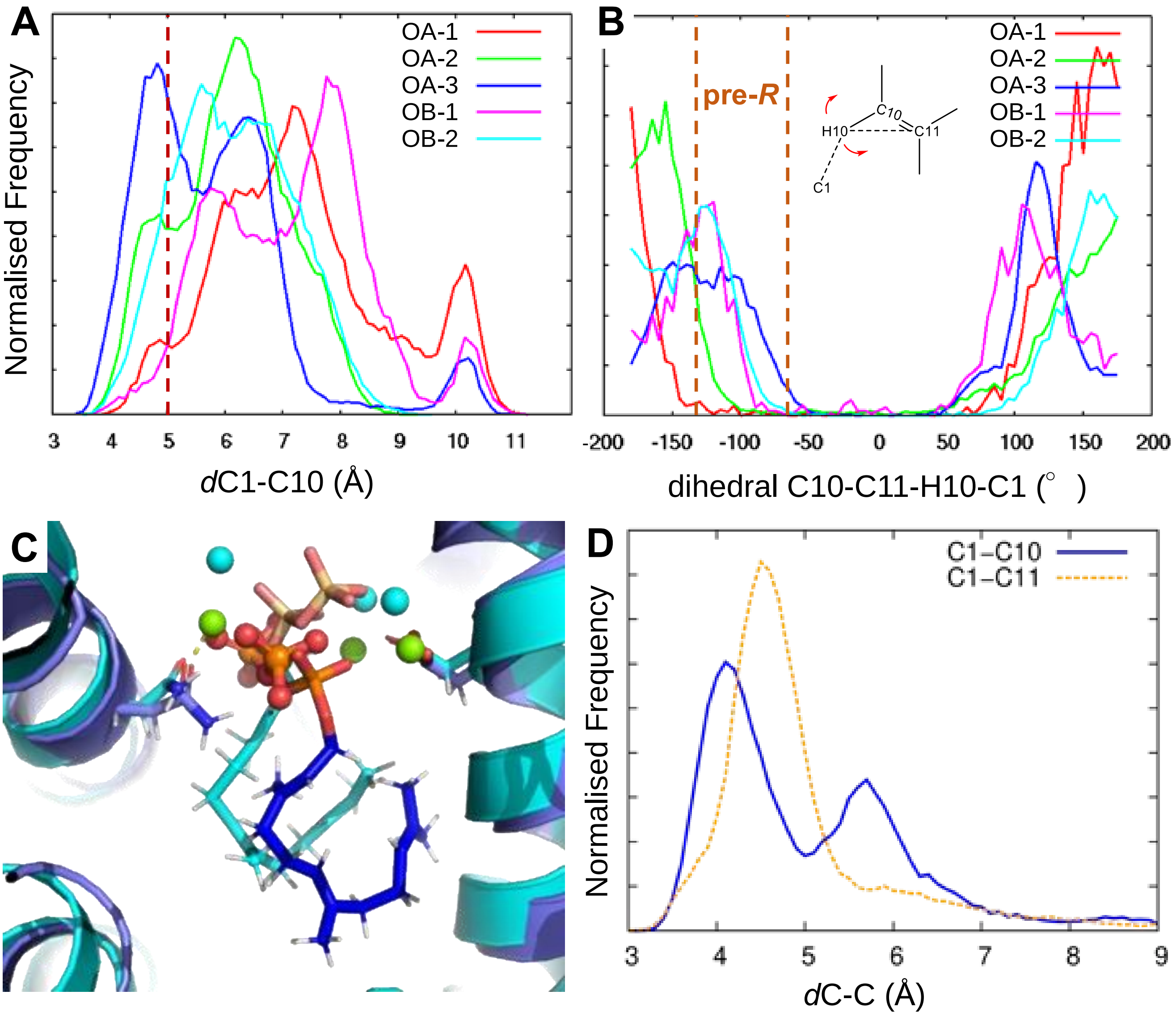

Figure S2. PTS-FDP simulations. **A**) Histograms of the C1-C10 cyclization distance (0.1 Å bin width). Only OA-2, OA-3 and OB-2 starting positions lead to substantial sampling < 5 Å. Data are obtained from 10-30 ns from 4 independent simulations of each starting position. **B**) Histograms of the dihedral angle C10-C11-H10-C1 (5° bin width), only for those snapshots with *d*C1-C10 < 5 Å. Only OA-3, OB-1 and OB-2 sample frequently between –125 and –55°, indicative of poses consistent with *R*-germacryl cation formation. **C**) Example snapshots for OA-3 (blue carbons, green Mg^{2+} ions) and OB-2 (cyan carbons and Mg^{2+} ions, pale diphosphate) with *d*C1-C10 < 5. For reference, the diphosphate from the starting structure is shown in ball and stick. **D**) Histograms of the C1-C10 and C1-C11 cyclization distances (0.1 Å bin width). Data from 10-30 ns from 4 independent simulations of two different minimized snapshots from the initial OA-3 simulations.

Isolepidozene orientation and water interaction in Gd11olS variants

In MD simulations of isolepidozene (and PPi) bound to Gd11olS$_{WT}$, water (that is not coordinated to one of the Mg2+ ions) approaches closely, in line with the expected hydroxylation, when C11 of isolepidozene is directed to the RQH site (**Figure S3C**, **Figure 2C**). This is not the case when isolepidozene is directed

to the G-helix, providing further evidence to our previous suggestion that hydroxylation most likely occurs from the RQH site. For Gd11olS$_{H\alpha\text{-}1}$, water only rarely approaches C11 of isolepidozene closely (both when directed to the RQH or the G-helix site), consistent with a significant reduction in hydroxylation.

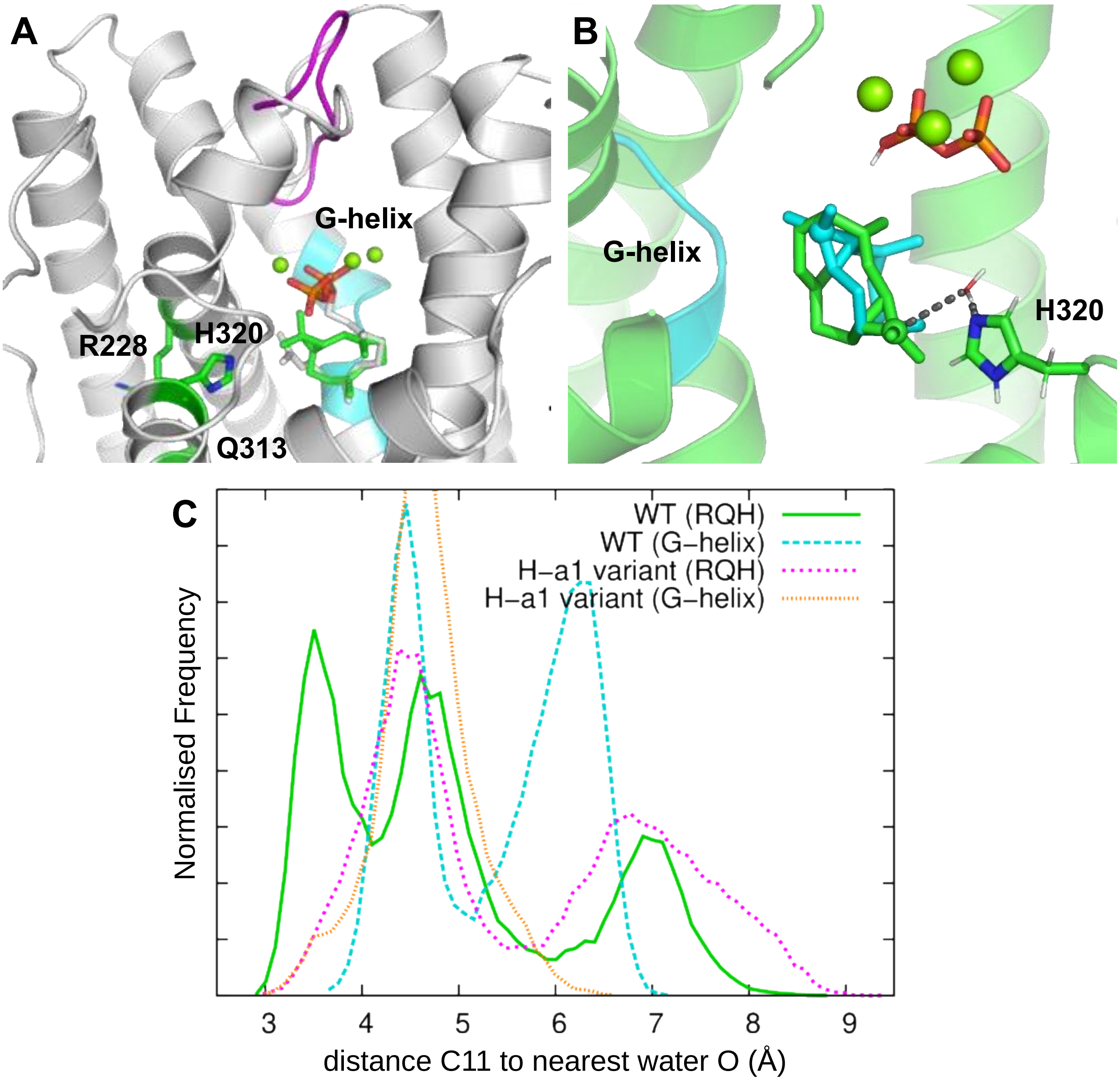

Figure S3. Gd11olS simulations in complex with isolepidozene. **A)** Starting structure of the Gd11olS-isolepidozene complex, with isolepidozene in green and the previously identified FDP conformation in gray. The starting structure for the Hα-1 loop in Gd11olS$_{H\alpha\text{-}1}$ is shown in magenta. Residues of the RQH site shown in green and sticks, and the G-helix kink region is coloured cyan. Hydrogens omitted for clarity. **B)** Representative structures of isolepidozene after short simulation times, either directing C11 (shown as sphere) to the G-helix kink (isolepidozene in cyan) or to His320 (isolepidozene in green). Example water interaction shown (see also main text, **Figure 2C**). Isolepidozene hydrogens omitted for clarity. **C)** Histograms (0.1 Å bin width) of the distance between C11 (carbon to be hydroxylated in Gd11olS$_{WT}$) and the oxygen of the closest water that is not coordinating to an Mg^{2+} ion. Data obtained from 10-30 ns in 5 (for RQH site) or 4 (G-helix site) independent simulations each.

11. Total ion chromatograms

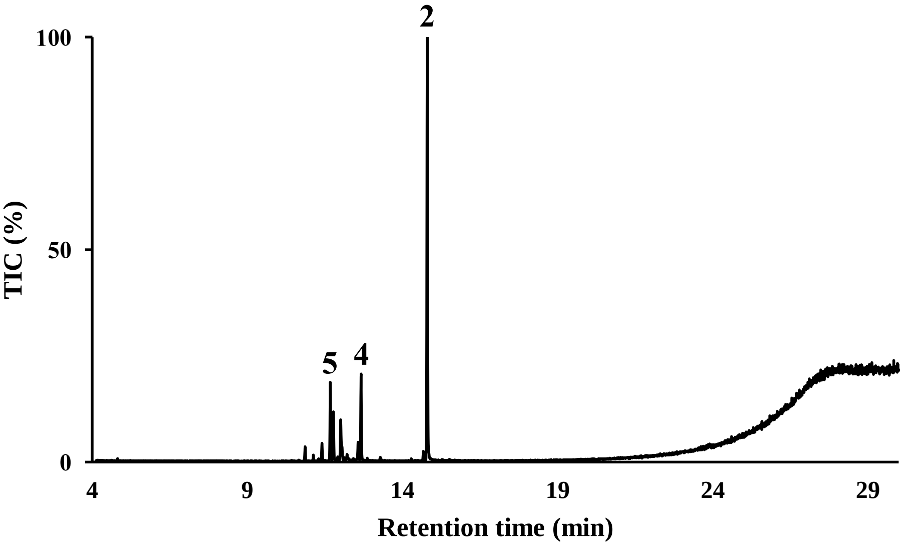

Figure S4. Total ion chromatogram of pentane extractable product arising from the incubation of (*E*,*E*)-FDP (**1**) with PTS_{WT}, producing patchoulol (**2**) as a major product along with number of sesquiterpene hydrocarbons.

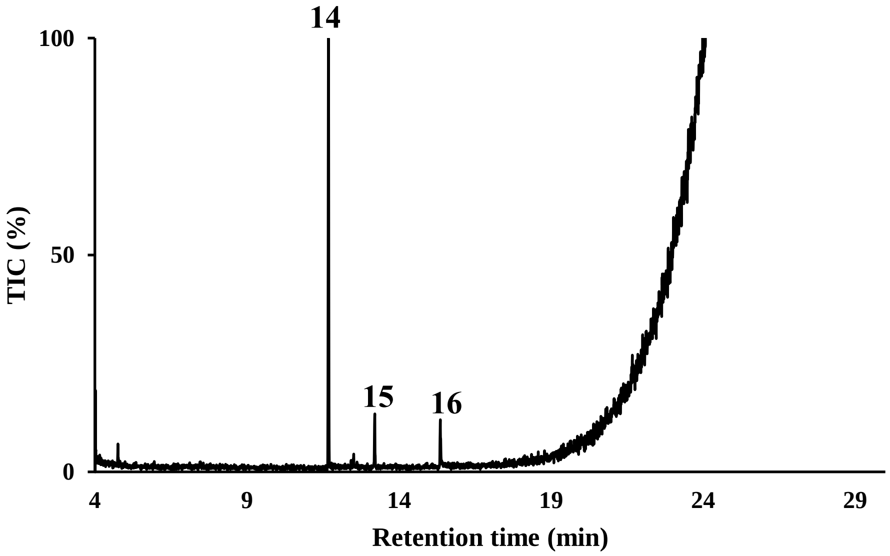

Figure S5A. Total ion chromatogram of pentane extractable products arising from the incubation of (*E*,*E*)-FDP (**1**) with PTS W276A, producing β-farnesene (**14**) as a major product along with small percentage of nerolidol (**15**), and farnesol (**16**).

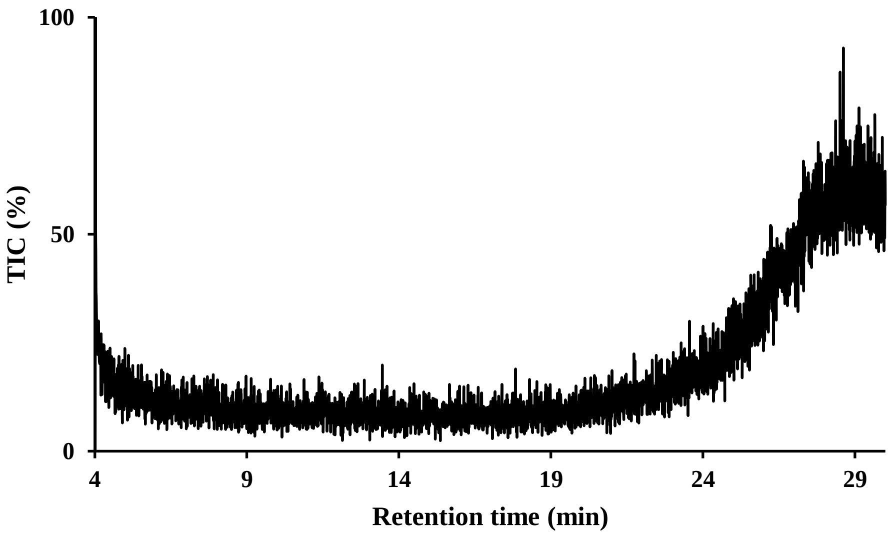

Figure S5B. Total ion chromatogram of pentane extractable products arising from the incubation of (*E*,*E*)-FDP (**1**) with buffer (50 mM Tris-HCl, pH 8.0) containing 5 mM $MgCl_2$ and 100 µM FDP without any enzyme, resulting in no product formation.

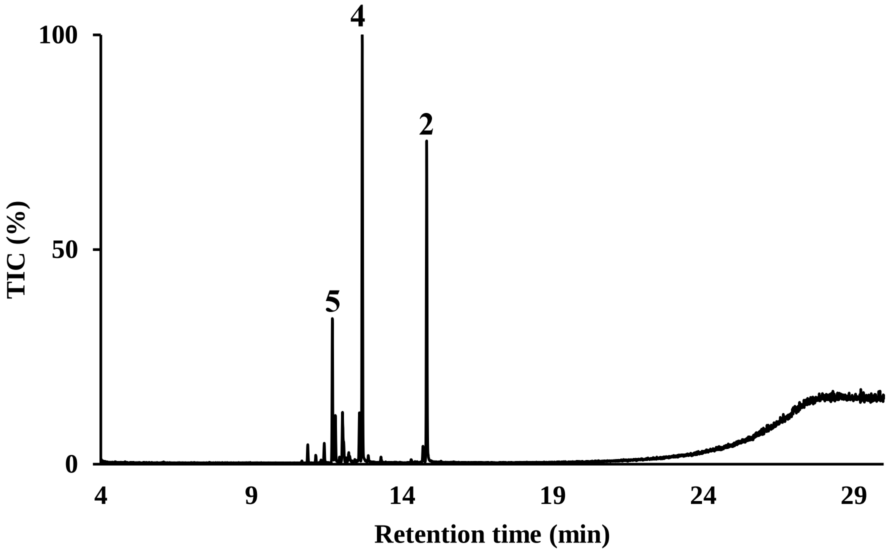

Figure S6. Total ion chromatogram of pentane extractable products arising from the incubation of (*E*,*E*)-FDP (**1**) with PTS C405A, producing α-bulnesene (**4**) as the major product along with a small percentage of patchoulol (**2**) and α-guaiene (**5**).

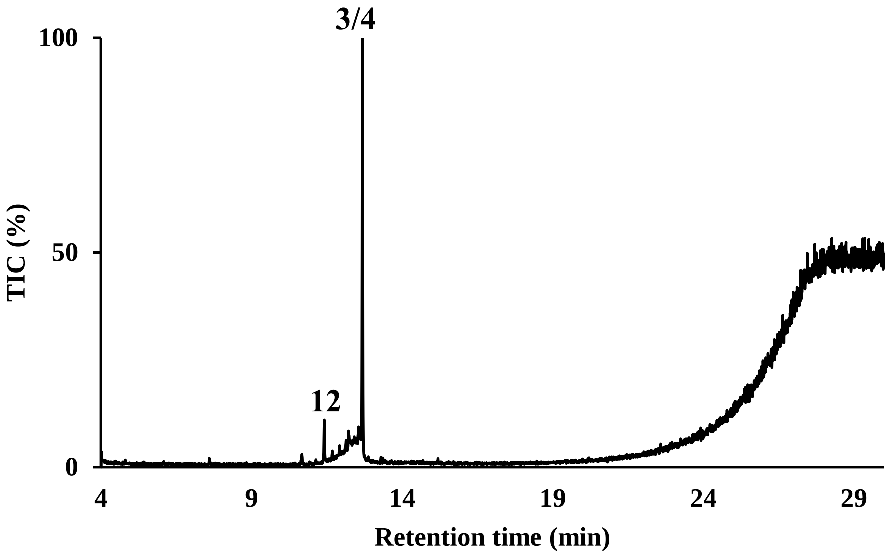

Figure S7. Total ion chromatogram of pentane extractable products arising from the incubation of (*E*,*E*)-FDP (**1**) with PTS Y525A, producing mixture of α-bulnesene and germacrene A (**3/4**) in equal ratio.

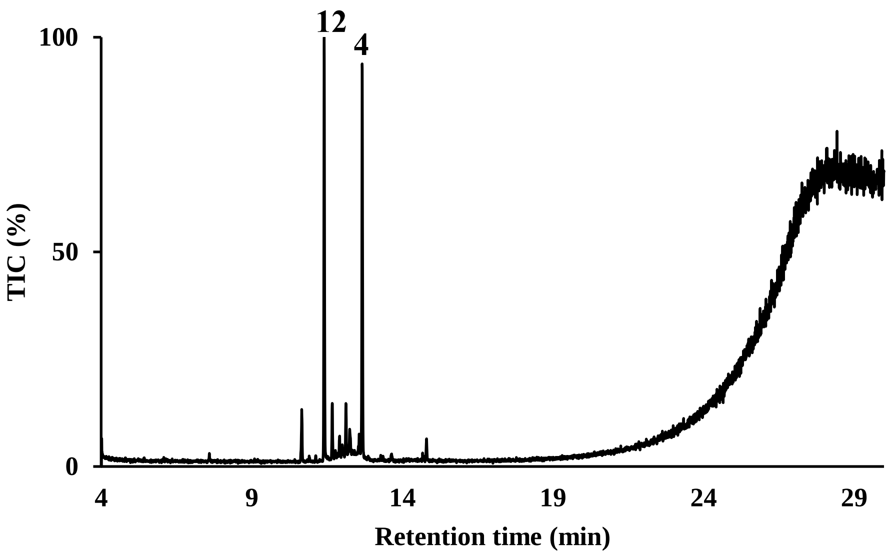

Figure S8. Total ion chromatogram of pentane extractable products arising from the incubation of (*E*,*E*)-FDP (**1**) with PTS Y525F, producing α-bulnesene (**4**) and β-caryophyllene (**12**).

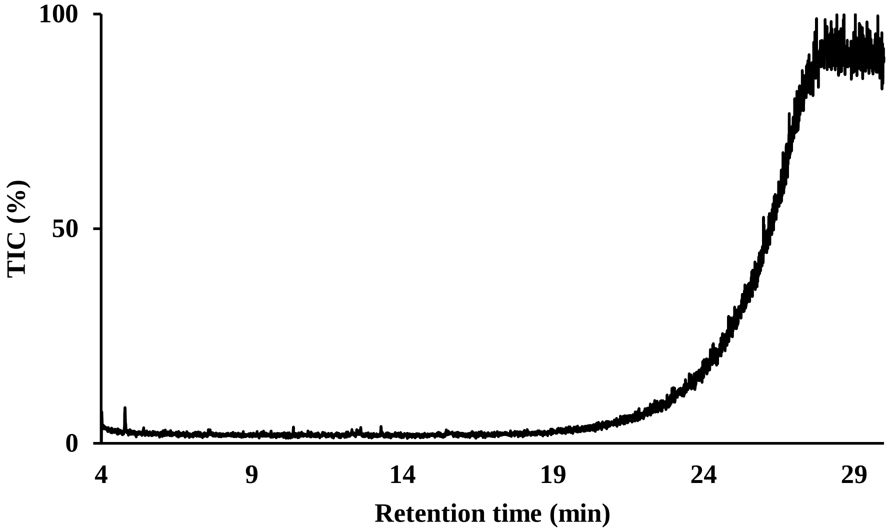

Figure S9. Total ion chromatogram of pentane extractable product(s) (inactive mutant) arising from the incubation of (*E*,*E*)-FDP (**1**) with PTS Y525W.

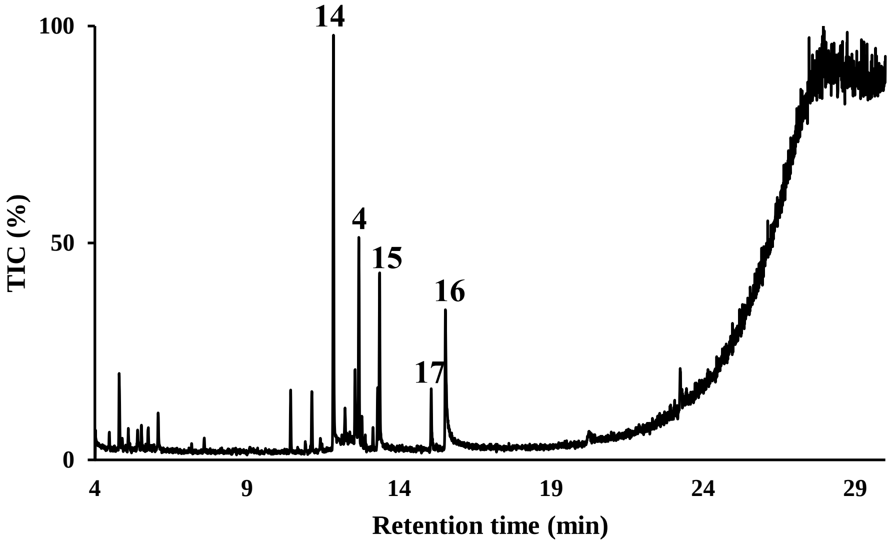

Figure S10. Total ion chromatogram of pentane extractable products arising from the incubation of (*E*,*E*)-FDP (**1**) with PTS Y531A, producing β-farnesene (**14**) as the major product along with nerolidol (**15**), farnesol (**16**) and α-bisabolol (**17**) with traces of α-bulnesene (**4**).

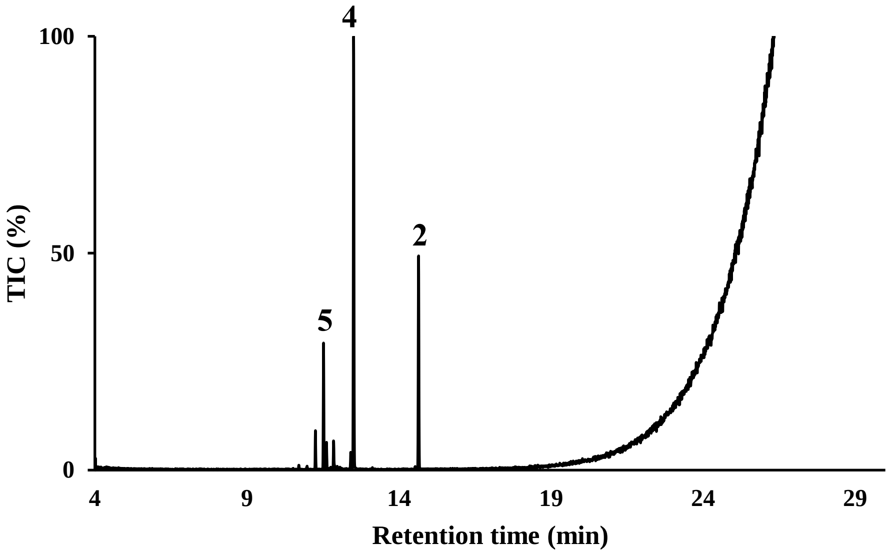

Figure S11. Total ion chromatogram of pentane extractable products arising from the incubation of (*E*,*E*)-FDP (**1**) with PTS Y531F, producing α-bulnesene (**4**) as a major product along with small percentage of patchoulol (**2**) and α-guaiene (**5**).

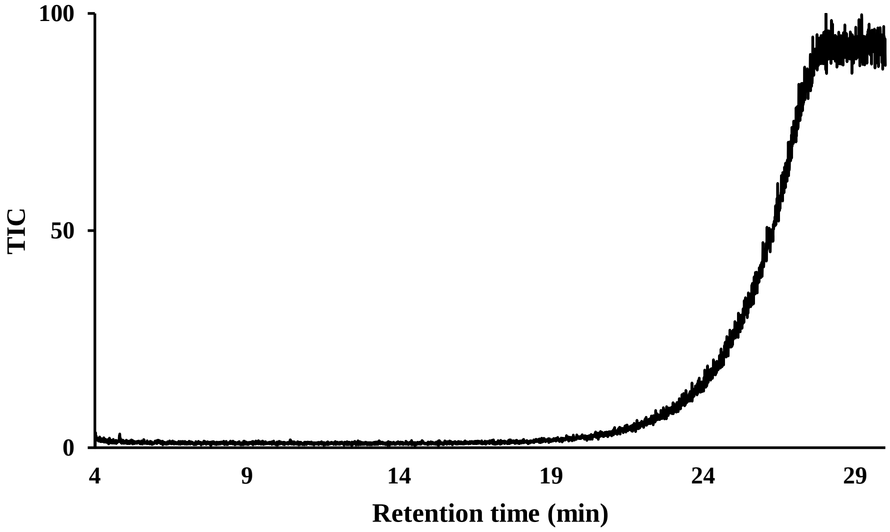

Figure S12. Total ion chromatogram of pentane extractable product(s) (inactive mutant) arising from the incubation of (*E*,*E*)-FDP (**1**) with Gd11olS$_{H\alpha\text{-}1}$ loop variant (8-residues replacement variant, ^{238}VEDEGELS^{245} of Gd11olS was replaced with the equivalent residues in SdS ^{233}RRGSGYYL^{240}.

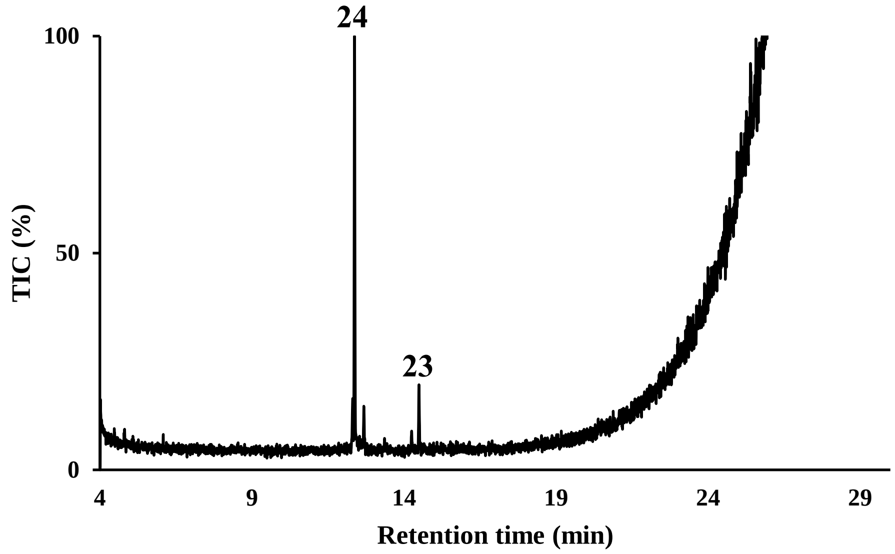

Figure S13. Total ion chromatogram of pentane extractable products arising from the incubation of (*E*,*E*)-FDP (**1**) with Gd11olS$_{H\alpha\text{-}1}$ loop variant (4-residues replacement variant, ^{238}VEDE^{241} of Gd11olS was replaced by equivalent residues in SdS ^{233}RRGS^{236}), producing isolepidozene (**24**) as a major product along with traces of germacradien-11-ol (**23**), germacrene D (**11**) and germacrene A (**3**).

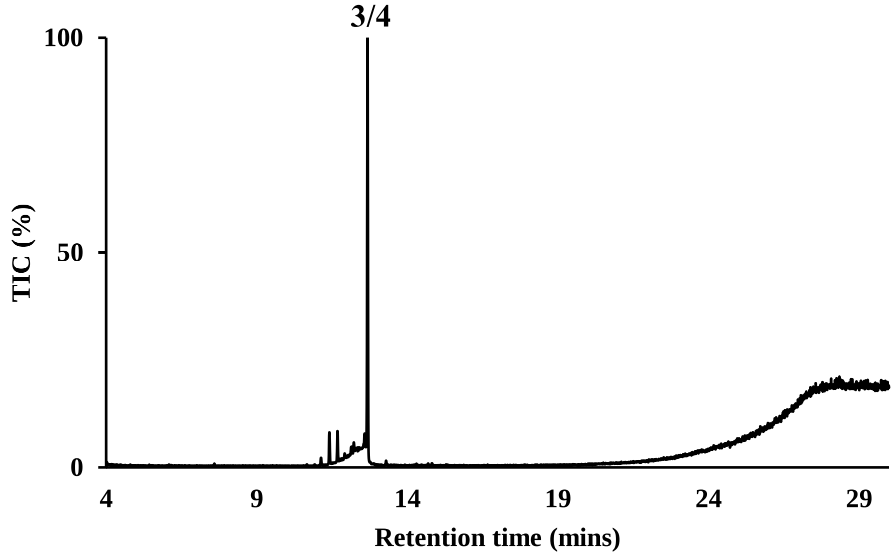

Figure S14. Total ion chromatogram of pentane extractable products arising from the incubation of (*E*,*E*)-FDP (**1**) with PTS$_{H\alpha\text{-}1}$ variant (4-residues replacement, ^{458}KKRE^{461} of PTS was replaced by equivalent residues in SdS ^{233}RRGS^{236}), producing a mixture of α-bulnesene (**4**) and germacrene A (**3**) in equal proportion. Both these compounds co-elute at same retention time in under these conditions.

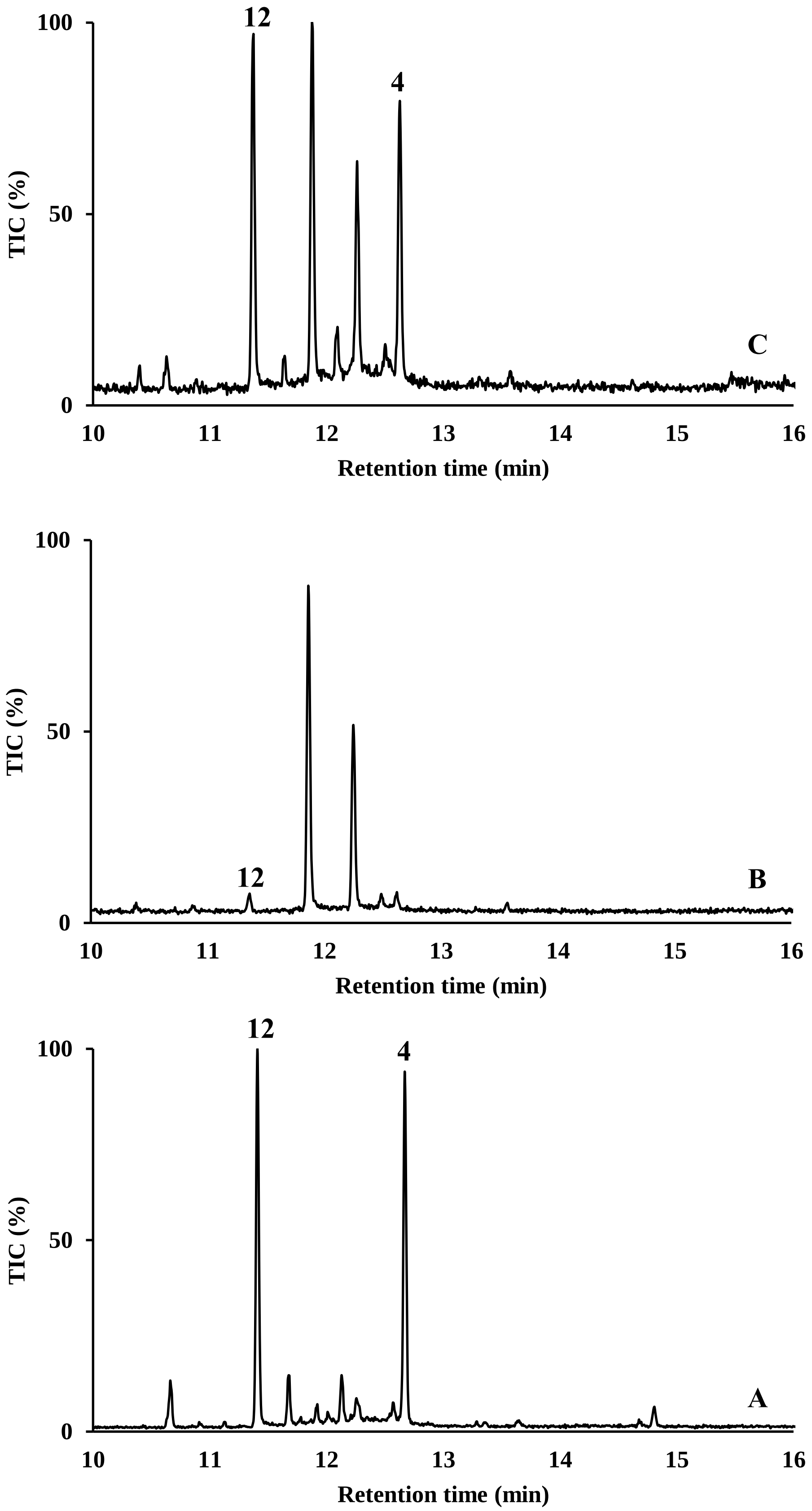

Figure S15A. Coinjection of pentane extractable products arising from the incubation of (*E*,*E*)-FDP (**1**) with PTS Y525F and *Solidago canadensis* germacrene A synthase (GAS) mutant G402C,[11] for the characterization of β-caryophyllene (**12**). Total ion chromatograms of pentane extractable products arising from the incubation of **A)** PTS Y525F with (*E*,*E*)-FDP (**1**), **B)** GAS 402C with (*E*,*E*)-FDP (**1**), **C)** PTS Y525F assay mixture co-injected with GAS G402C assay mixture.

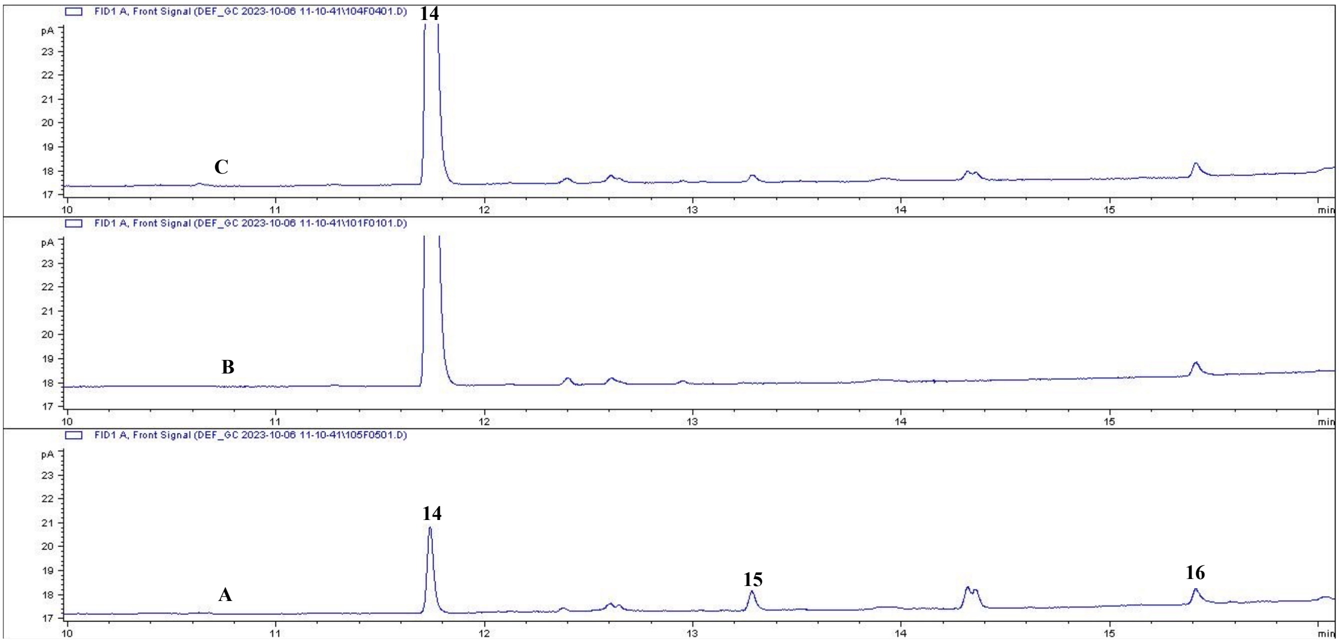

Figure S15B: Coinjection of pentane extractable products arising from the incubation of (*E*,*E*)-FDP (**1**) with PTS W276A and β-farnesene authentic standard for the characterization of β-farnesene (**14**) on GC using chiral stationary phase. **A)** PTS W276A upon incubation with (*E*,*E*)-FDP (**1**), **B)** β-farnesene authentic standard (**14**), **C)** PTS W276A assay mixture co-injected with β-farnesene authentic standard.

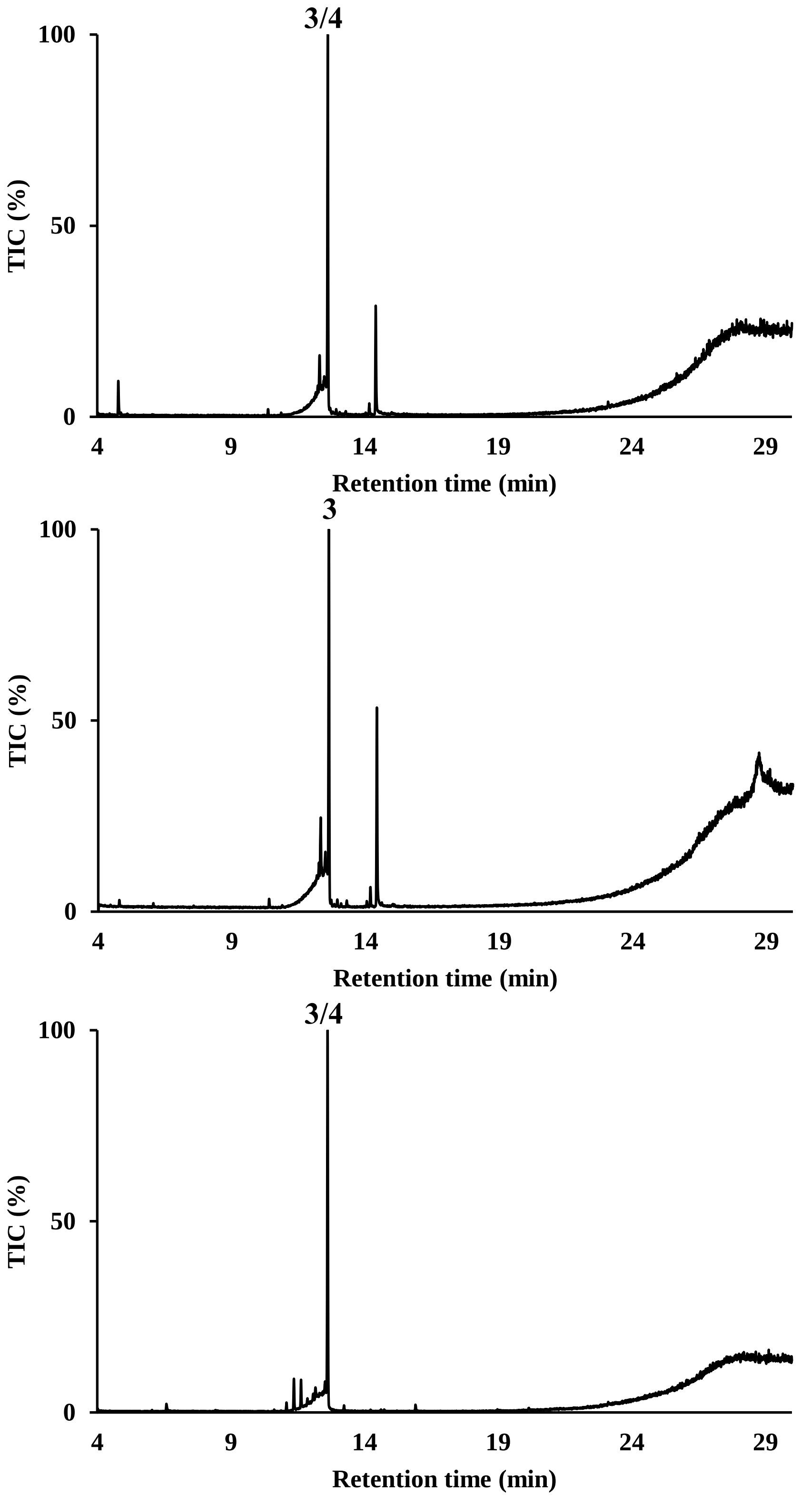

Figure S16. Coinjection of pentane extractable products arising from the incubation of (*E*,*E*)-FDP (**1**) with $PTS_{H\alpha\text{-}1}$ loop variant and *Streptomyces coelicolor* germacradien-11-ol synthase (Gd11olS) mutant W312F,[12] for the characterization of germacrene A (**3**) co-eluting with α-bulenesene in GCMS. Total ion chromatograms of pentane extractable products arising from the incubation of **A)** $PTS_{H\alpha\text{-}1}$ loop variant with (*E*,*E*)-FDP (**1**), **B)** Gd11olS W312F with (*E*,*E*)-FDP (**1**), **C)** $PTS_{H\alpha\text{-}1}$ loop variant assay mixture co-injected with Gd11olS W312F assay mixture.

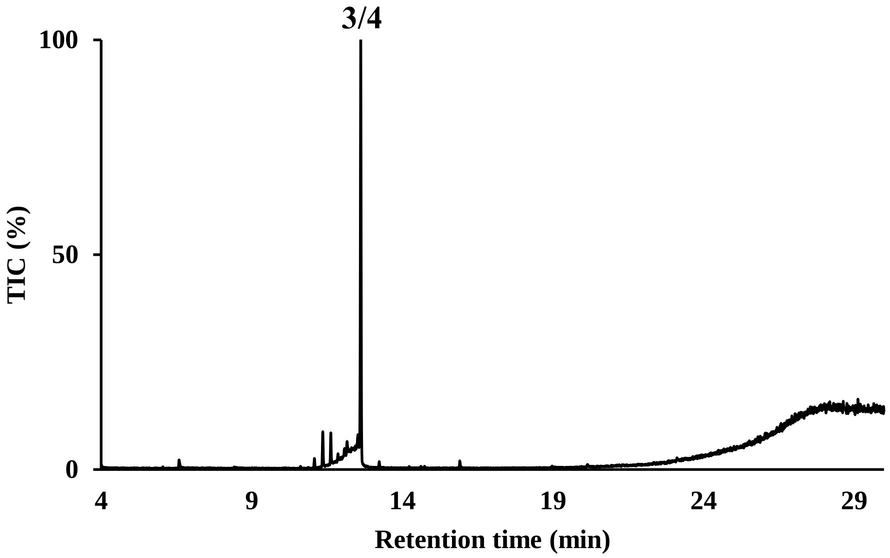

Figure S17. Total ion chromatograms of pentane extractable products arising from the incubation of PTS$_{H\alpha\text{-}1}$ loop variant large-scale assay with (*E*,*E*)-FDP (**1**) producing α-bulnesene **(4)** and germacrene A **(3)** as a mixture with traces of β-caryophyllene (**12**) and α-guaiene (**5**). All these products were confirmed by NMR spectroscopy.

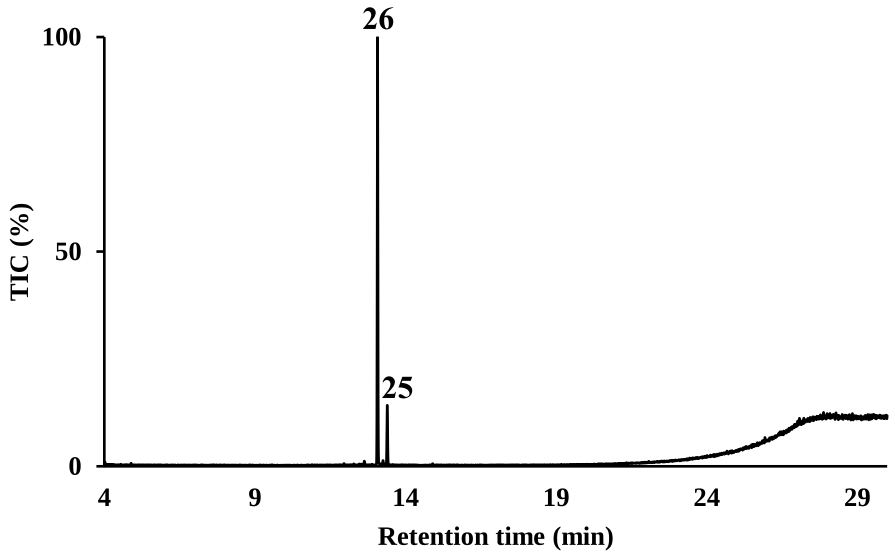

Figure S18. Total ion chromatogram of pentane extractable product arising from the incubation of (*E*,*E*)-FDP (**1**) with selina-4,(15),7(11)-diene synthase wild-type (SdS$_{WT}$), producing selina-4(15),7(11)-diene (**26**, 86.7%) as a major product along with small percentage of germacrene B (**25**, 13.3%).

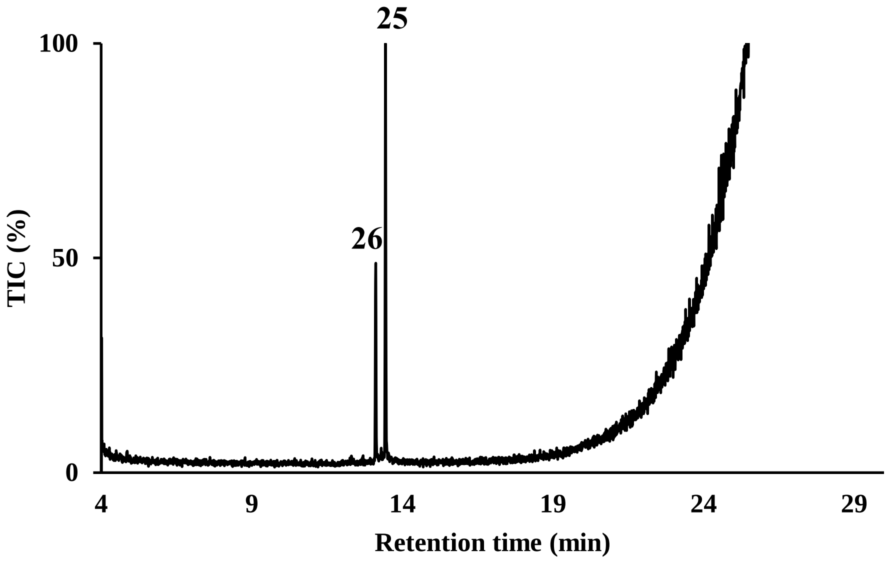

Figure S19. Total ion chromatogram of pentane extractable product arising from the incubation of (*E*,*E*)-FDP (**1**) with SdS$_{H\alpha\text{-}1}$ loop variant (8-residues replacement, ^{233}RRGSGYYL^{240} of SdS was replaced by equivalent residues in Gd11olS ^{238}VEDEGELS^{245}), producing germacrene B (**25**, 66.1%) as a major product along with reduced level of selina-4(15),7(11)-diene (**26**, 33.9%).

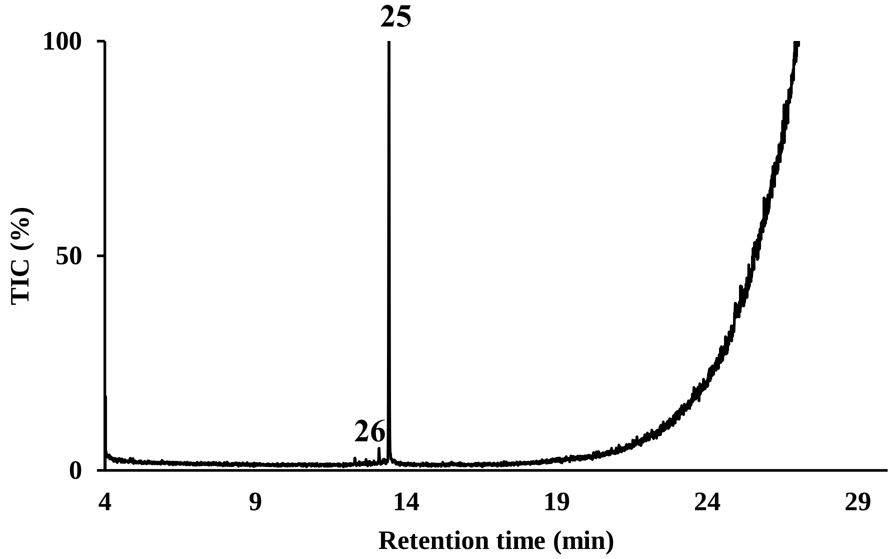

Figure S20. Total ion chromatogram of pentane extractable product arising from the incubation of (*E*,*E*)-FDP (**1**) with SdS$_{H\alpha\text{-}1}$ loop variant (4-residues replacement, ^{233}RRGS^{236} of SdS was replaced by equivalent residues in Gd11olS ^{238}VEDE^{241}), producing germacrene B (**25**, 97.7%) as a main product along with traces of selina-4(15),7(11)-diene (**26**, 2.3%).

12. GC analysis

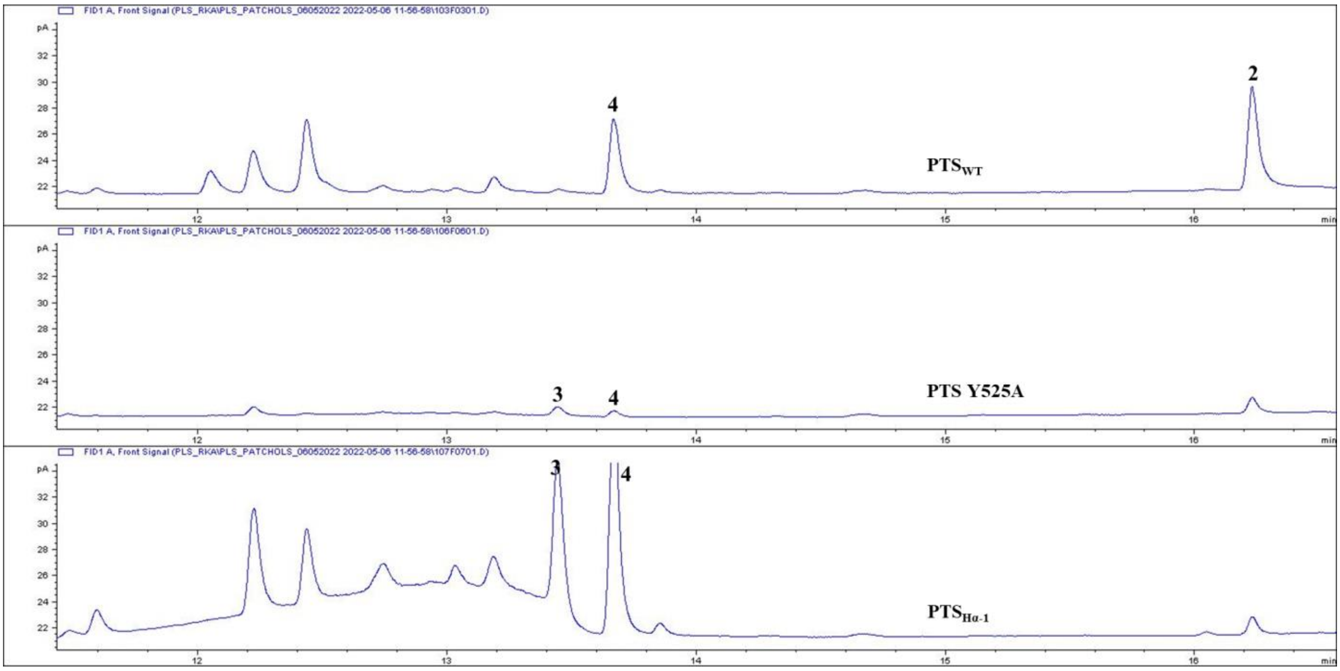

Figure S21: GC analysis using chiral stationary phase for the separation of α-bulnesene and germacrene A in the assay mixture of $PTS_{H\alpha\text{-}1}$ loop variant and mutants Y525A along with PTS. Patchoulol (**2**), germacrene A **(3),** α-bulnesene (**4**).

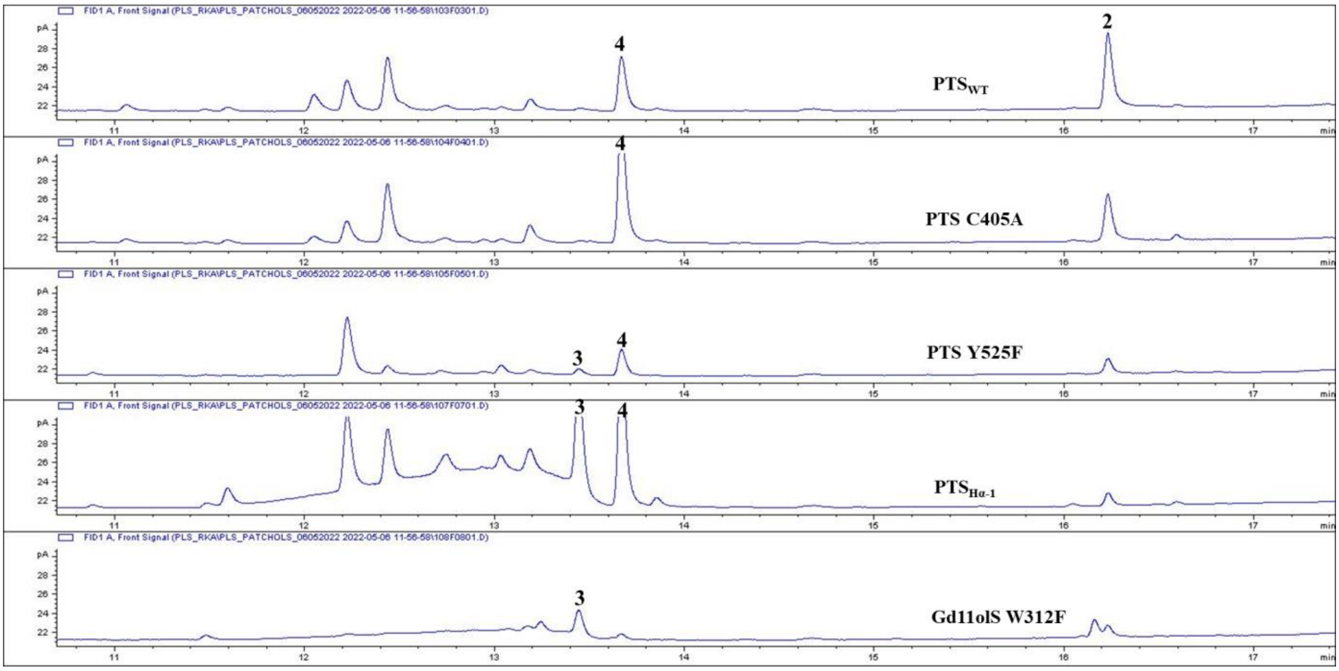

Figure S22: GC analysis using a chiral stationary phase for the separation of α-bulnesene and germacrene A in the assay mixture of $PTS_{H\alpha\text{-}1}$variant and mutants (Y525F, C405A and PTS) in comparison with Gd11olS W312F. Patchoulol (**2**), germacrene A **(3),** α-bulnesene (**4**).

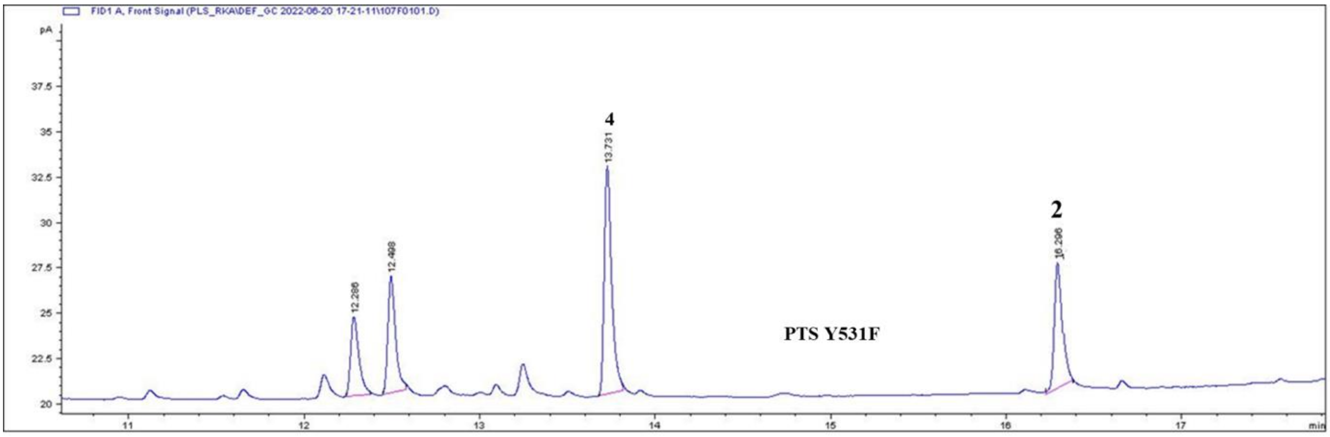

Figure S23: GC analysis using chiral stationary phase for the separation of α-bulnesene and germacrene A in the assay mixture of PTS Y531F, producing α-bulnesene (**4**) as a major product with reduced level of patchoulol (**2**).

13. GCMS mass spectra

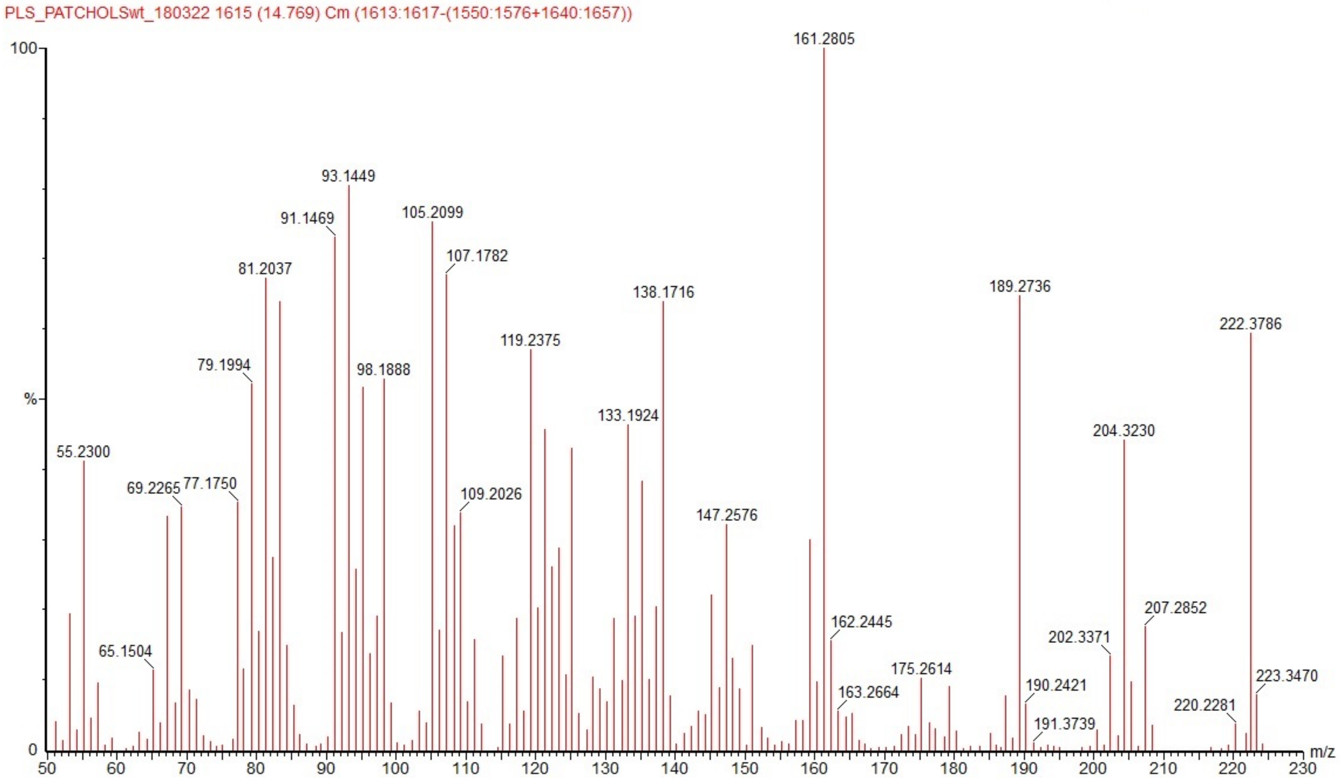

Figure S24. EI Mass spectrum of the compound eluting at 14.80 min in the gas-chromatogram from the incubation of (*E*,*E*)-FDP (**1**) with PTS_{WT} (patchoulol, **2**).

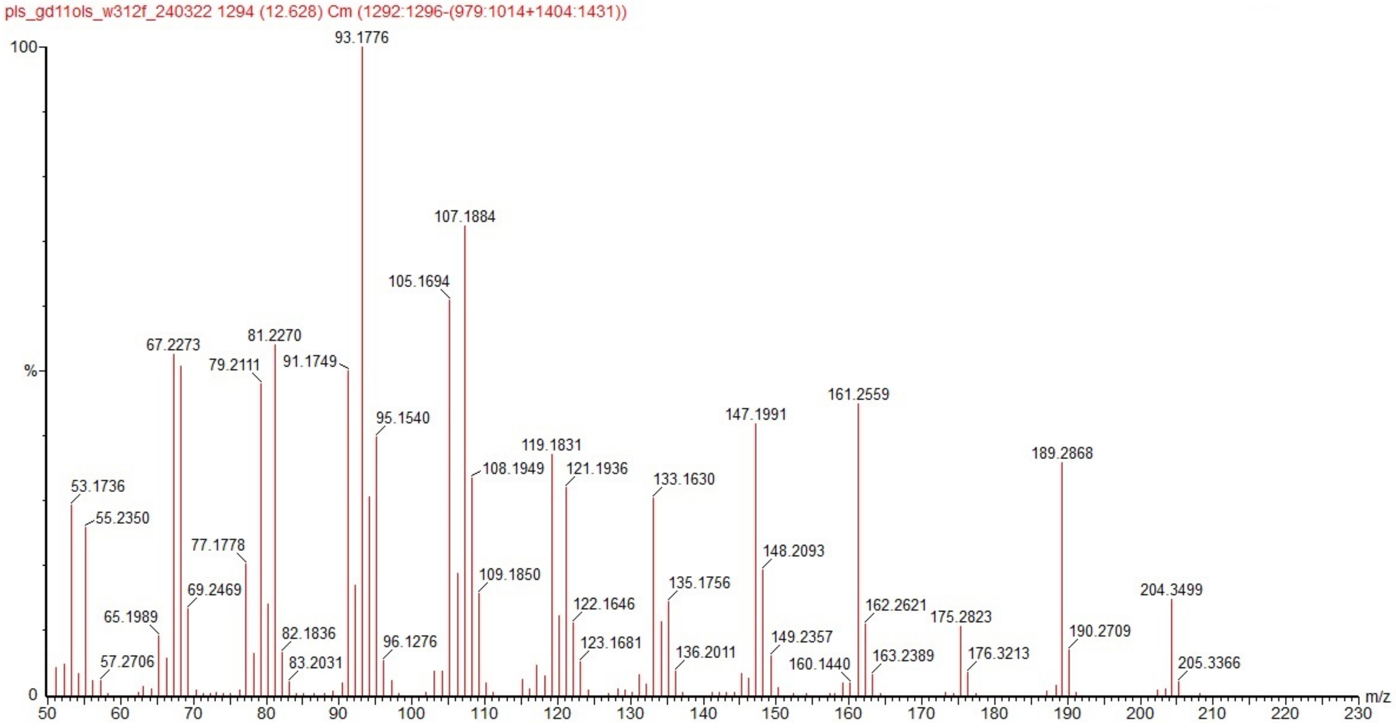

Figure S25. EI Mass spectrum of the compound eluting at 12.67 min in the gas-chromatogram from the incubation of (*E*,*E*)-FDP (**1**) with PTS Y525A, Y525F, $PTS_{H\alpha\text{-}1}$ and $Gd11olS_{H\alpha\text{-}1}$ variants (germacrene A, **3**).

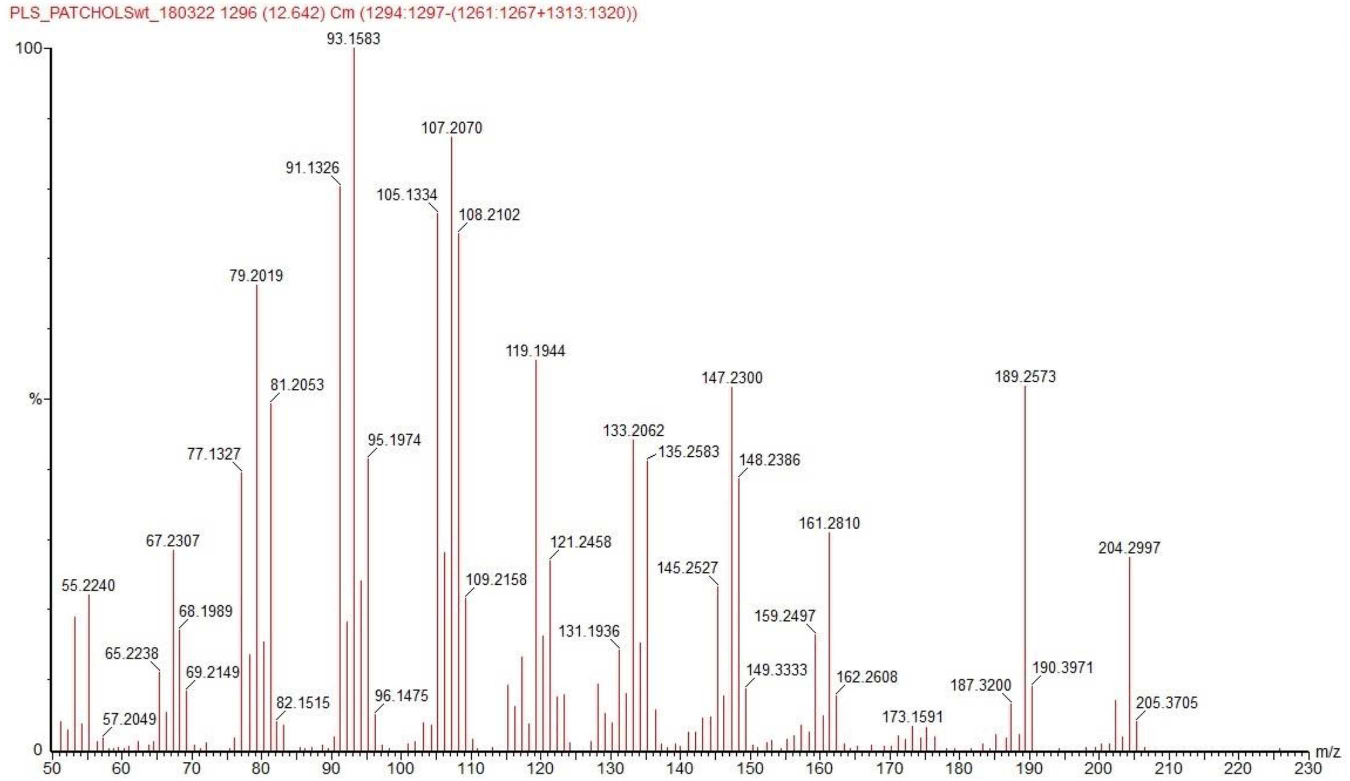

Figure S26. EI Mass spectrum of the compound eluting at 12.67 min in the gas-chromatogram from the incubation of (*E*,*E*)-FDP (**1**) with PTS_{WT} and all the variants except Y525W and C405F (α-bulnesene, **4**).

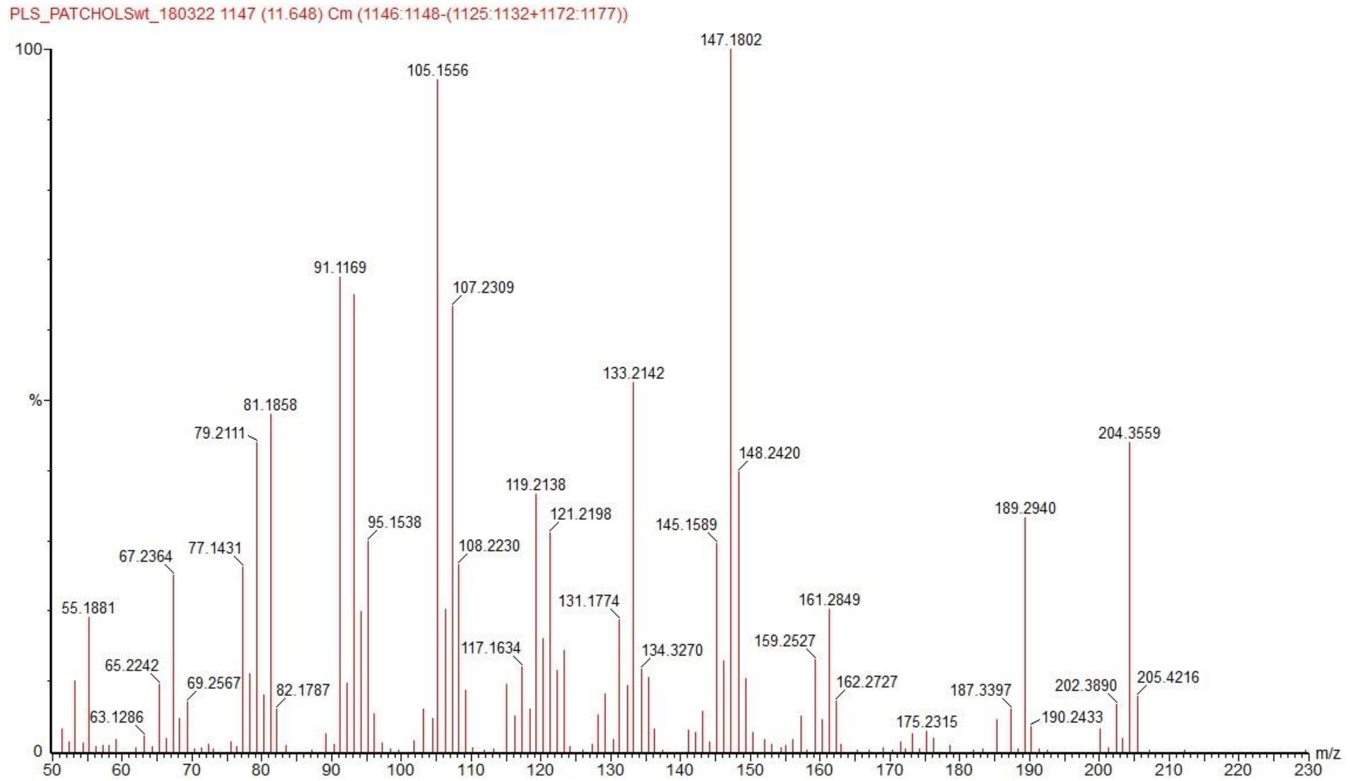

Figure S27. EI Mass spectrum of the compound eluting at 11.67 min in the gas-chromatogram from the incubation of (*E*,*E*)-FDP (**1**) with PTS_{WT} and all the variants except Y525W and C405F (α-guaiene, **5**).

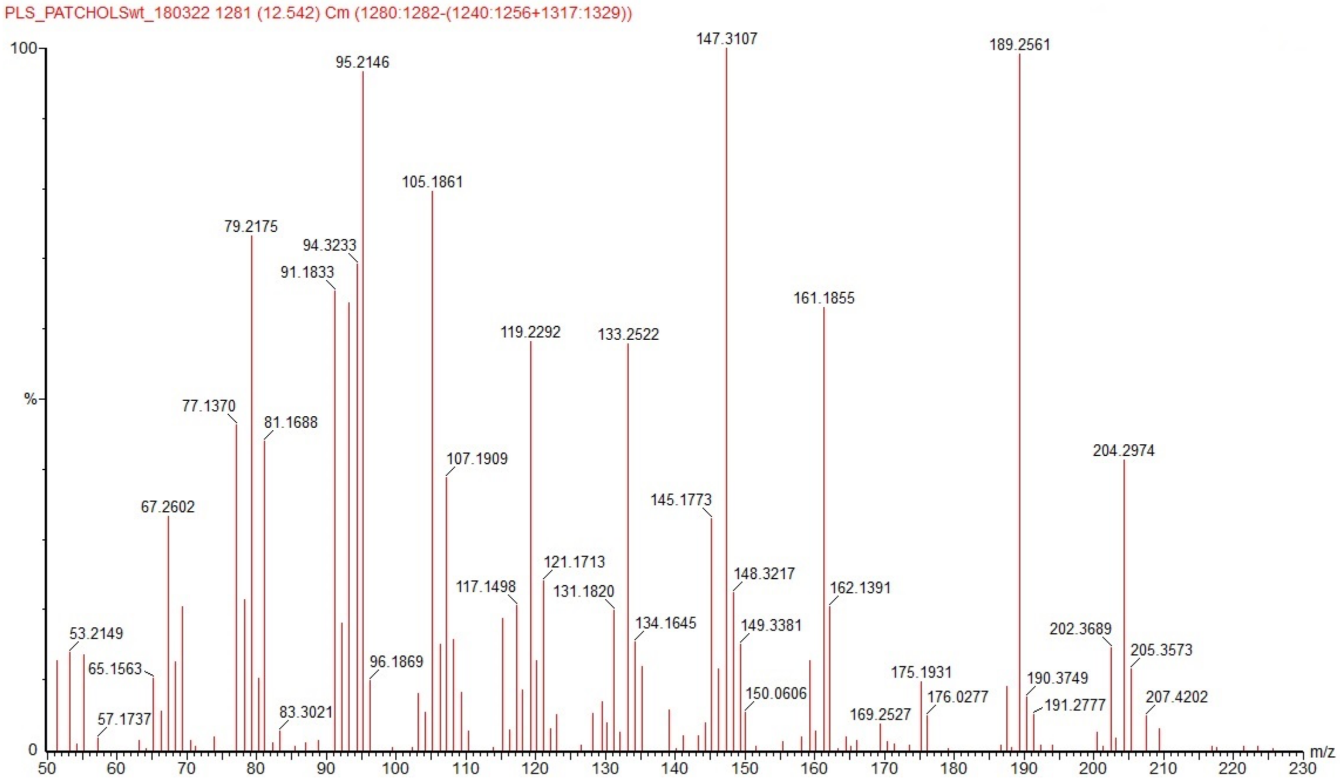

Figure S28. EI Mass spectrum of the compound eluting at 12.58 min in the gas-chromatogram from the incubation of (*E*,*E*)-FDP (**1**) with PTS_{WT} and all the variants except Y525W and C405F (guai-4,11-diene, **6**).

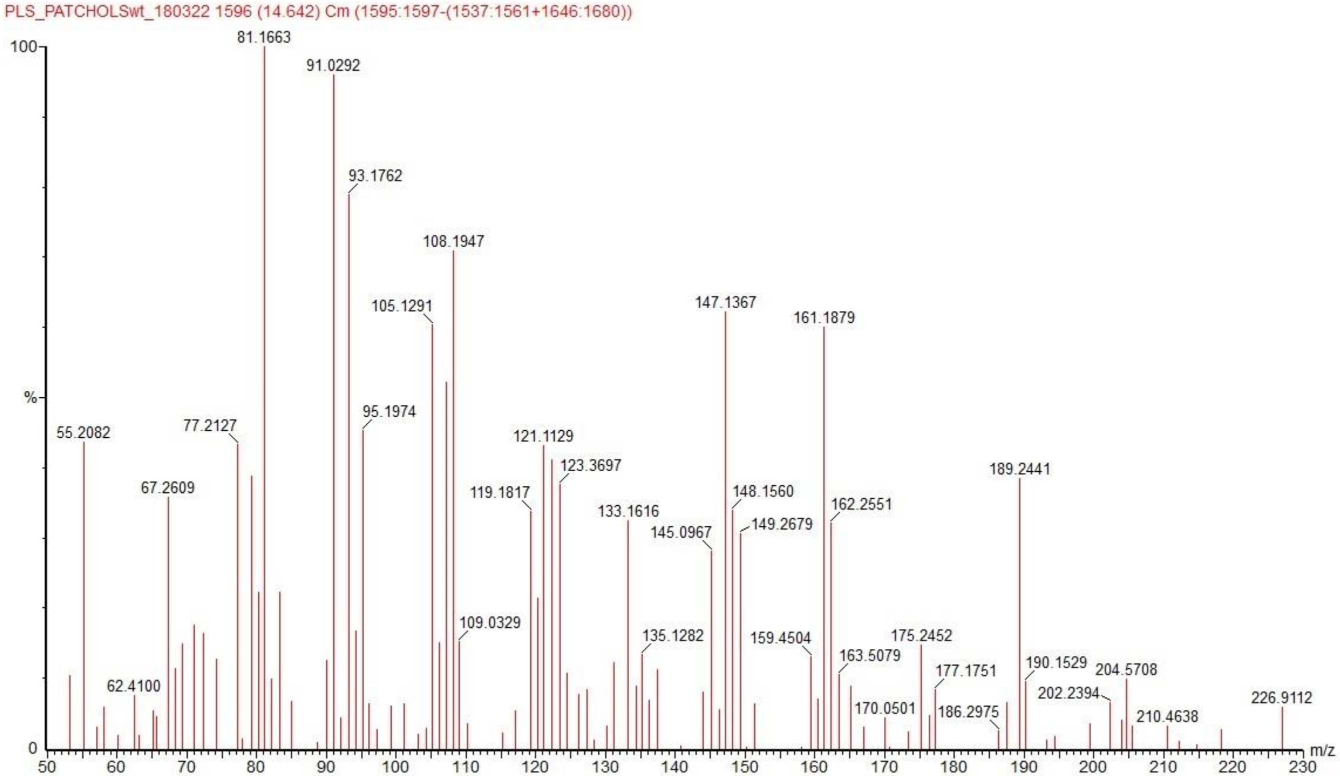

Figure S29. EI Mass spectrum of the compound eluting at 14.68 min in the gas-chromatogram from the incubation of (*E*,*E*)-FDP (**1**) with PTS_{WT} and C405A (pogostol, **7**).

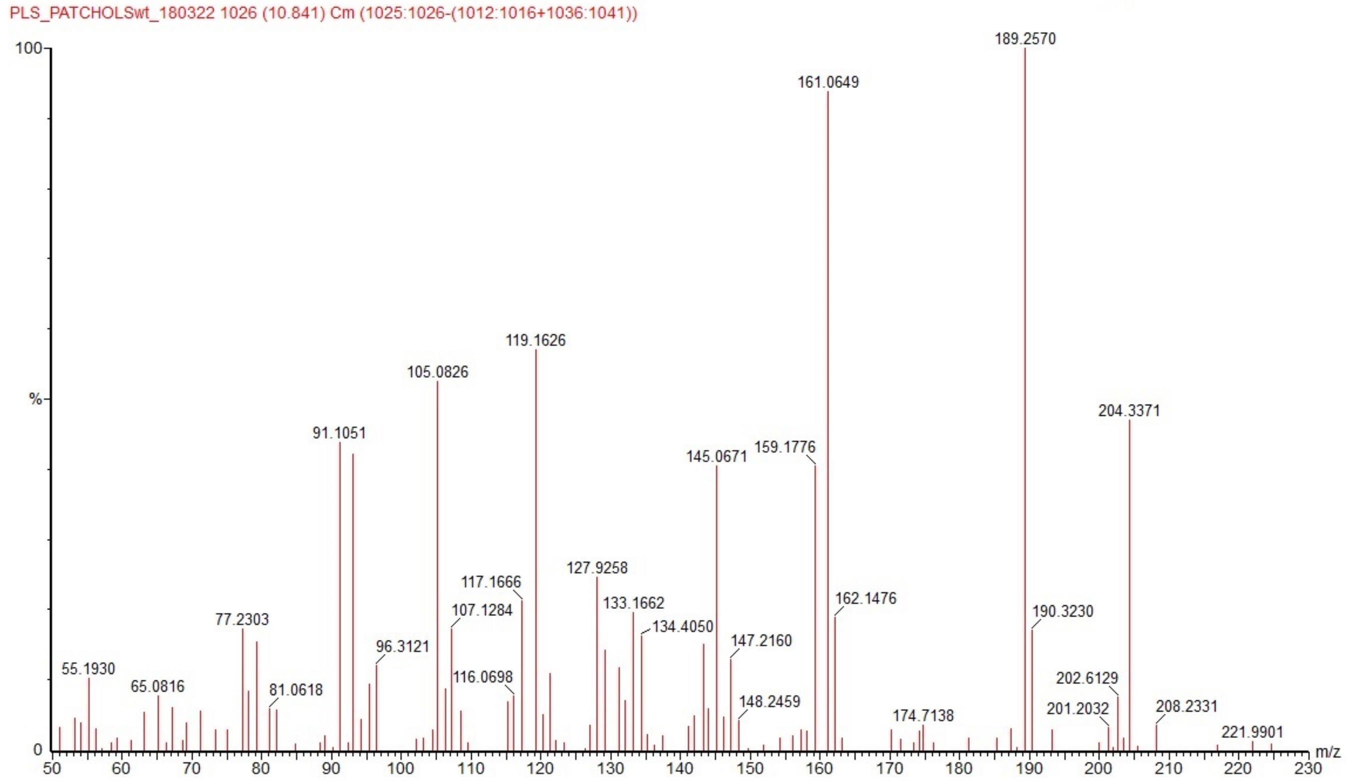

Figure S30. EI Mass spectrum of the compound eluting at 10.86 min in the gas-chromatogram from the incubation of (*E*,*E*)-FDP (**1**) with PTS$_{WT}$ and C405A (β-patchoulene, **8**).

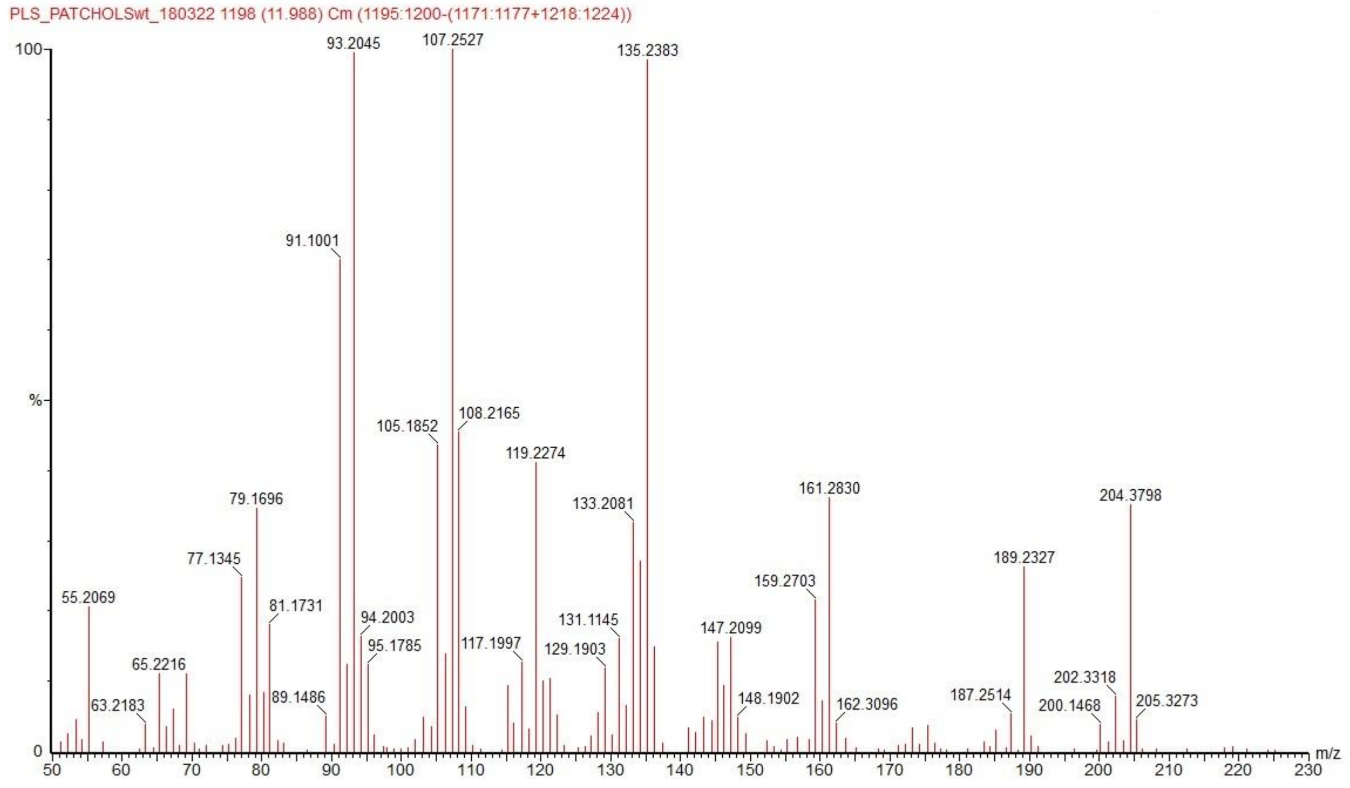

Figure S31. EI Mass spectrum of the compound eluting at 12.01 min in the gas-chromatogram from the incubation of (*E*,*E*)-FDP (**1**) with PTS$_{WT}$, C405A and Y531F (α-patchoulene, **9**).

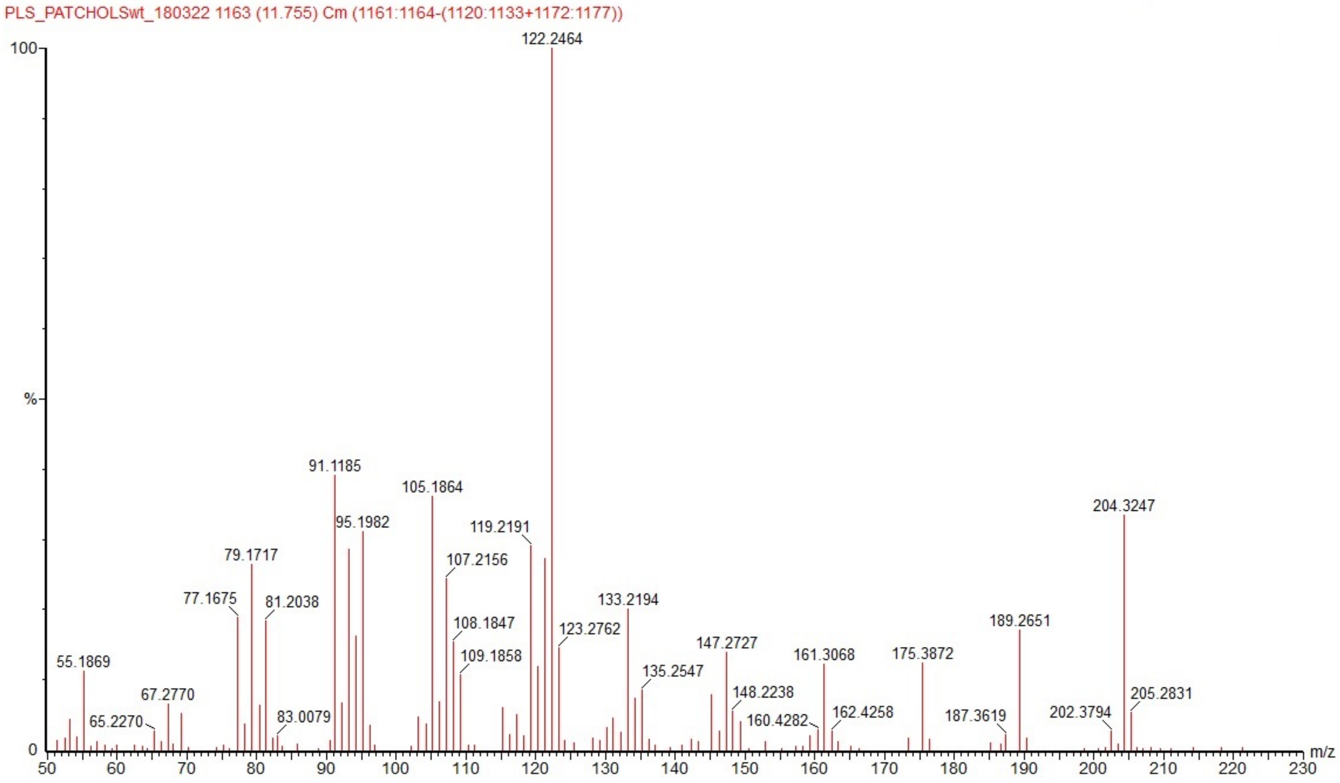

Figure S32. EI Mass spectrum of the compound eluting at 11.77 min in the gas-chromatogram from the incubation of (*E*,*E*)-FDP (**1**) with PTS_{WT}, C405A and Y531F (seychellene, **10**).

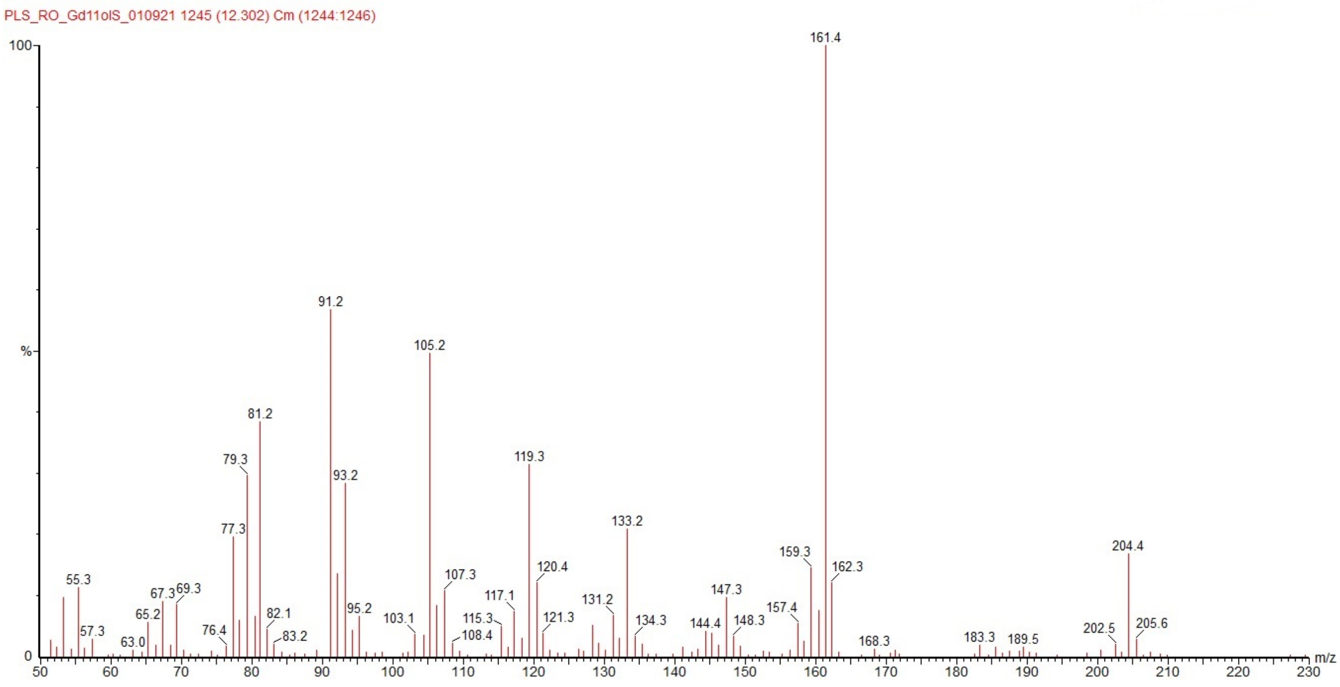

Figure S33. EI Mass spectrum of the compound eluting at 12.23 min in the gas-chromatogram from the incubation of (*E*,*E*)-FDP (**1**) with PTS_{WT} and Y525A (germacrene D, **11**).

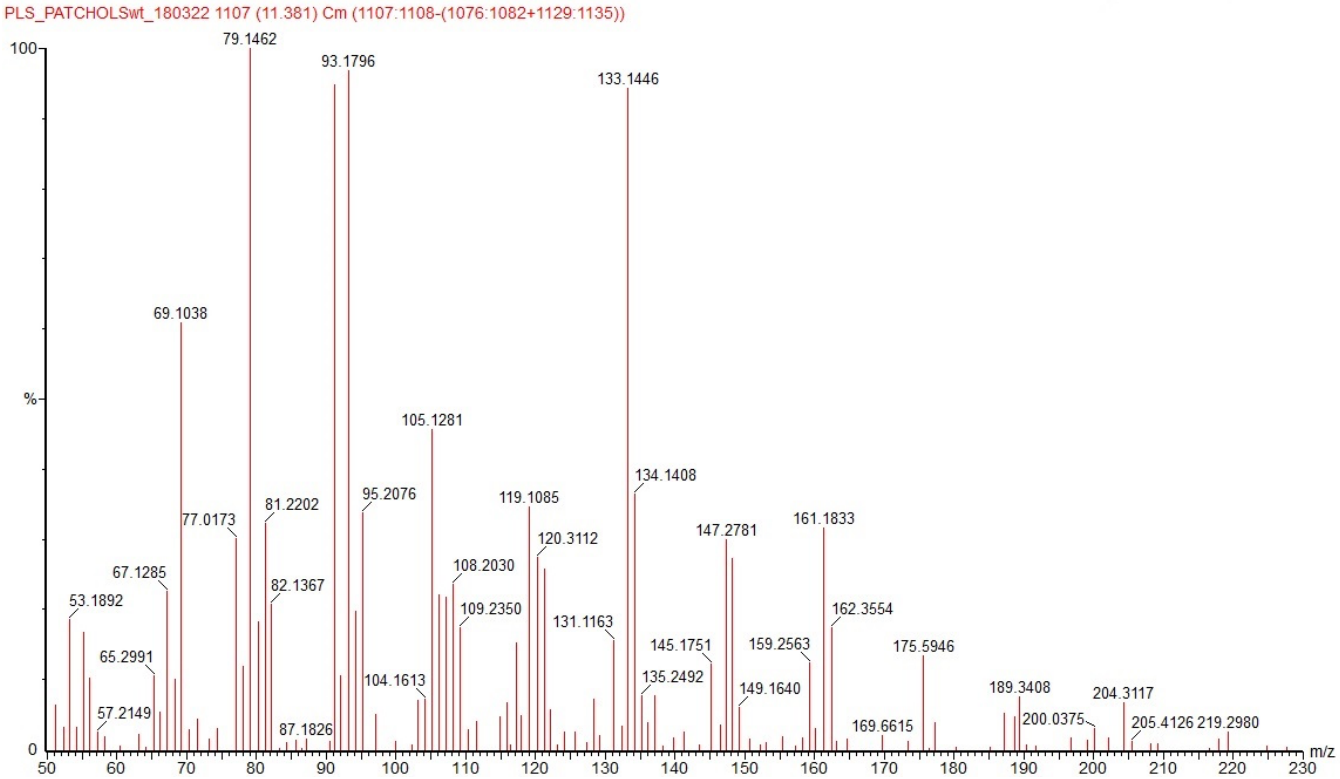

Figure S34. EI Mass spectrum of the compound eluting at 11.41 min in the gas-chromatogram from the incubation of (*E*,*E*)-FDP (**1**) with PTS$_{WT}$ and all the variants except Y525W and C405F (β-caryophyllene, **12**).

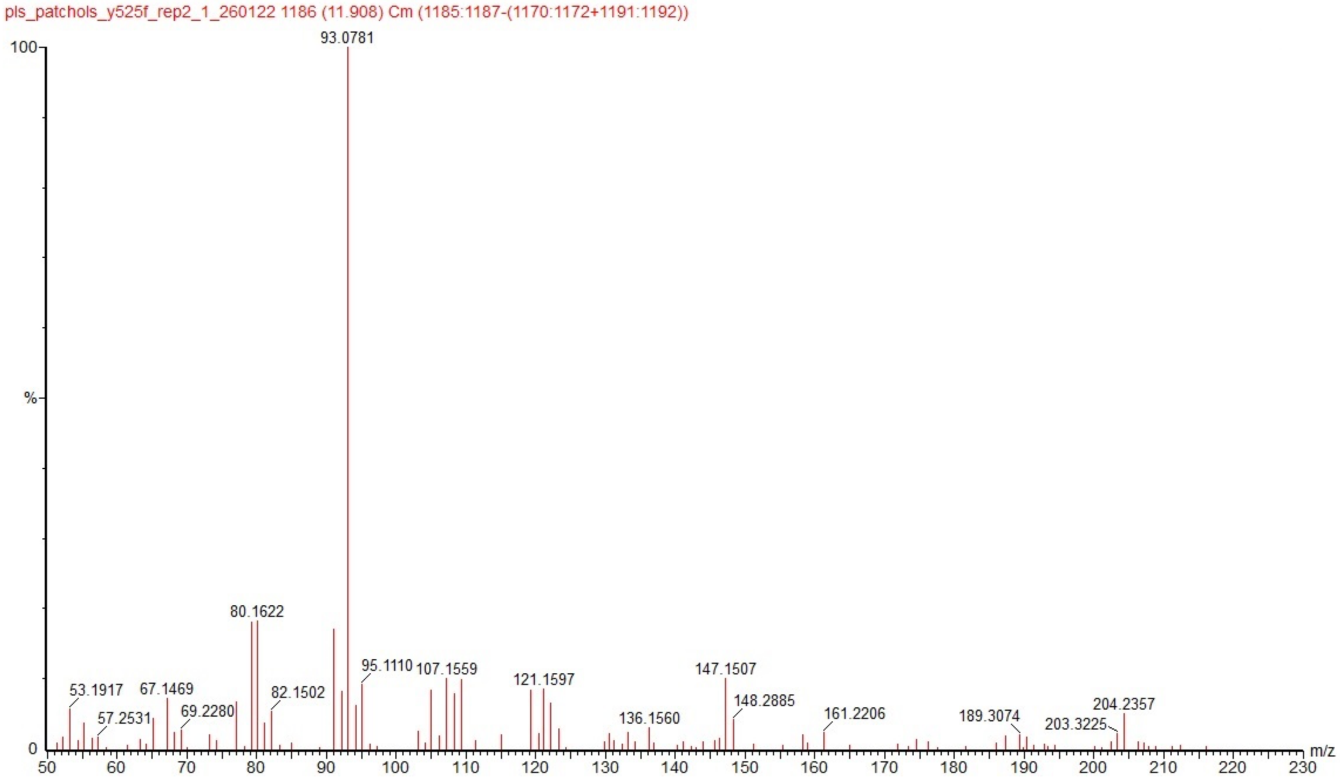

Figure S35. EI Mass spectrum of the compound eluting at 11.91 min in the gas-chromatogram from the incubation of (*E*,*E*)-FDP (**1**) with PTS$_{WT}$ and Y525F (α-humulene, **13**).

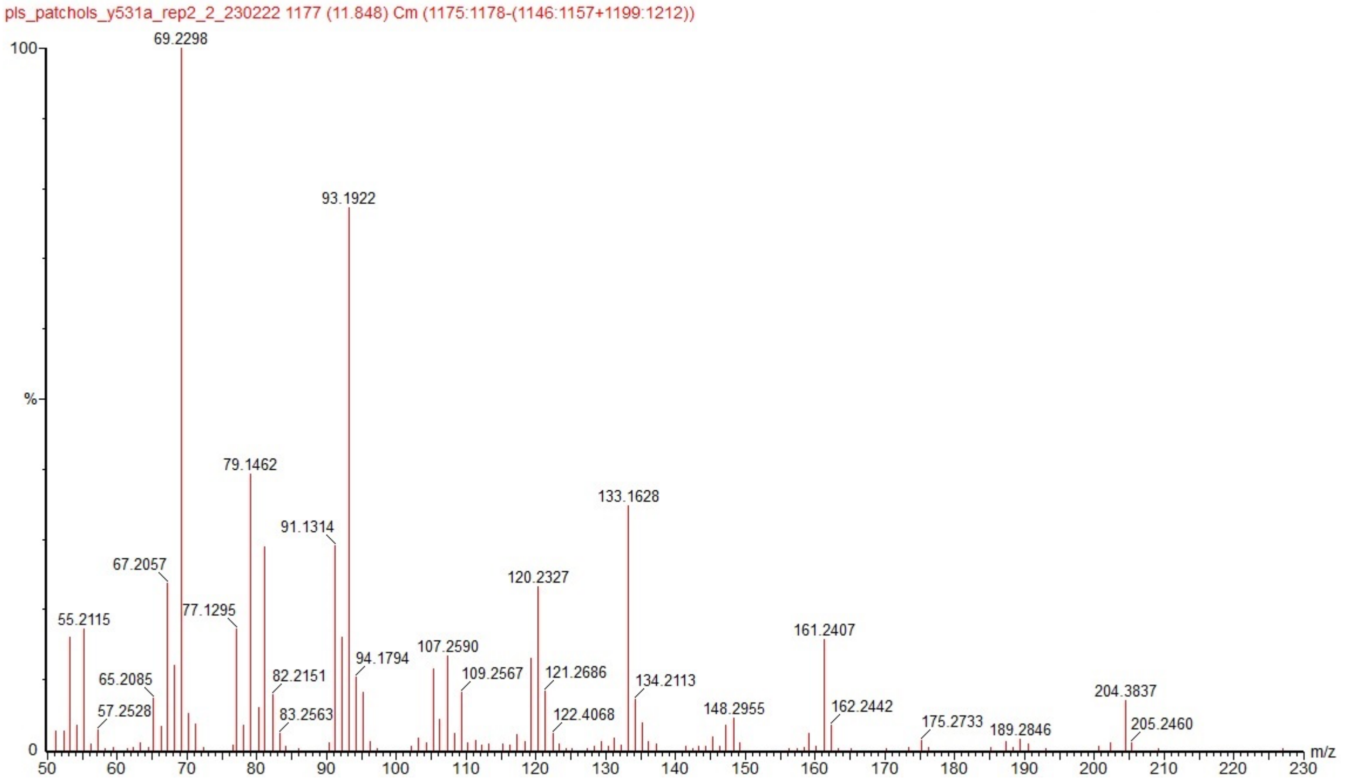

Figure S36. EI Mass spectrum of the compound eluting at 11.84 min in the gas-chromatogram from the incubation of (*E*,*E*)-FDP (**1**) with PTS_{WT} Y531A and W276A (β-farnesene, **14**).

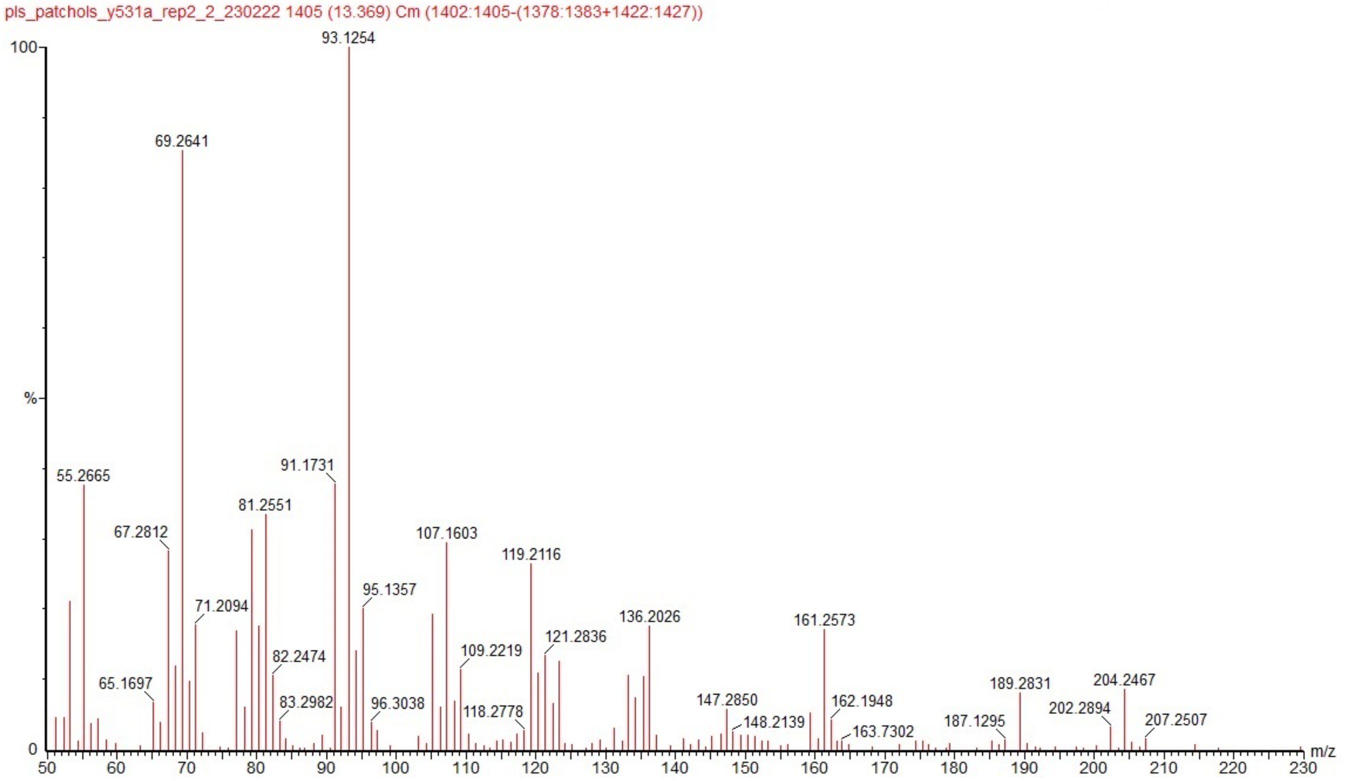

Figure S37. EI Mass spectrum of the compound eluting at 13.36 min in the gas-chromatogram from the incubation of (*E*,*E*)-FDP (**1**) with PTS_{WT} Y531A and W276A ((*E*)-nerolidol, **15**).

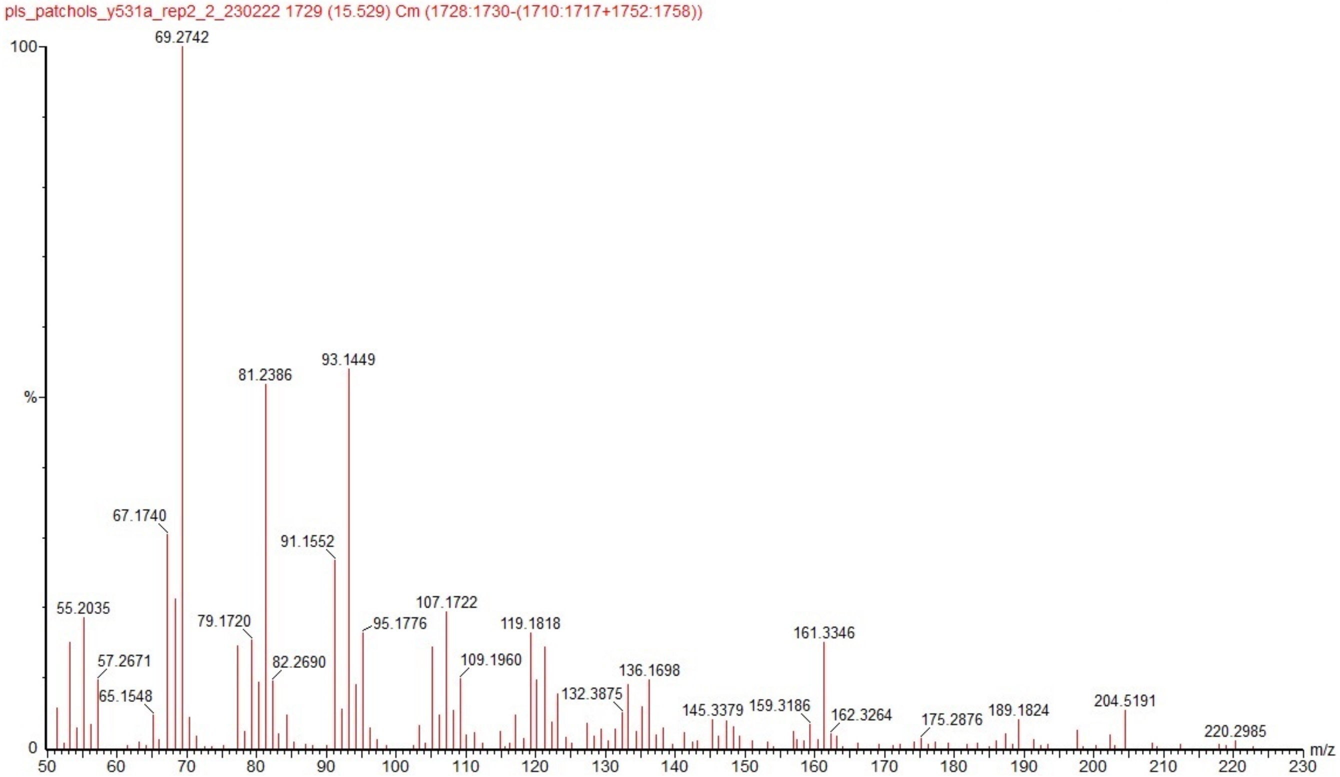

Figure S38. EI Mass spectrum of the compound eluting at 15.52 min in the gas-chromatogram from the incubation of (*E*,*E*)-FDP (**1**) with PTS$_{WT}$ Y531A and W276A ((*E*,*E*)farnesol, **16**).

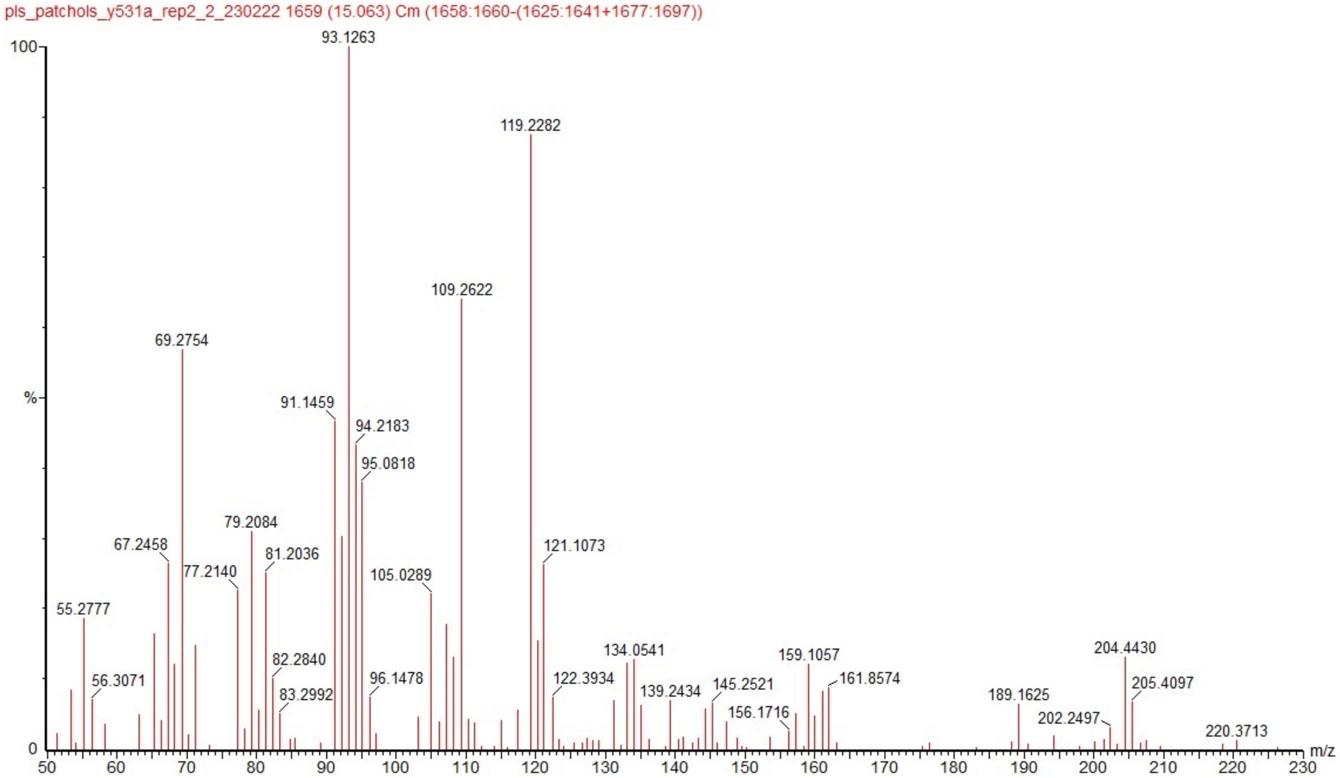

Figure S39. EI Mass spectrum of the compound eluting at 15.06 min in the gas-chromatogram from the incubation of (*E*,*E*)-FDP (**1**) with PTS$_{WT}$ Y531A (α-bisabolol, **17**).

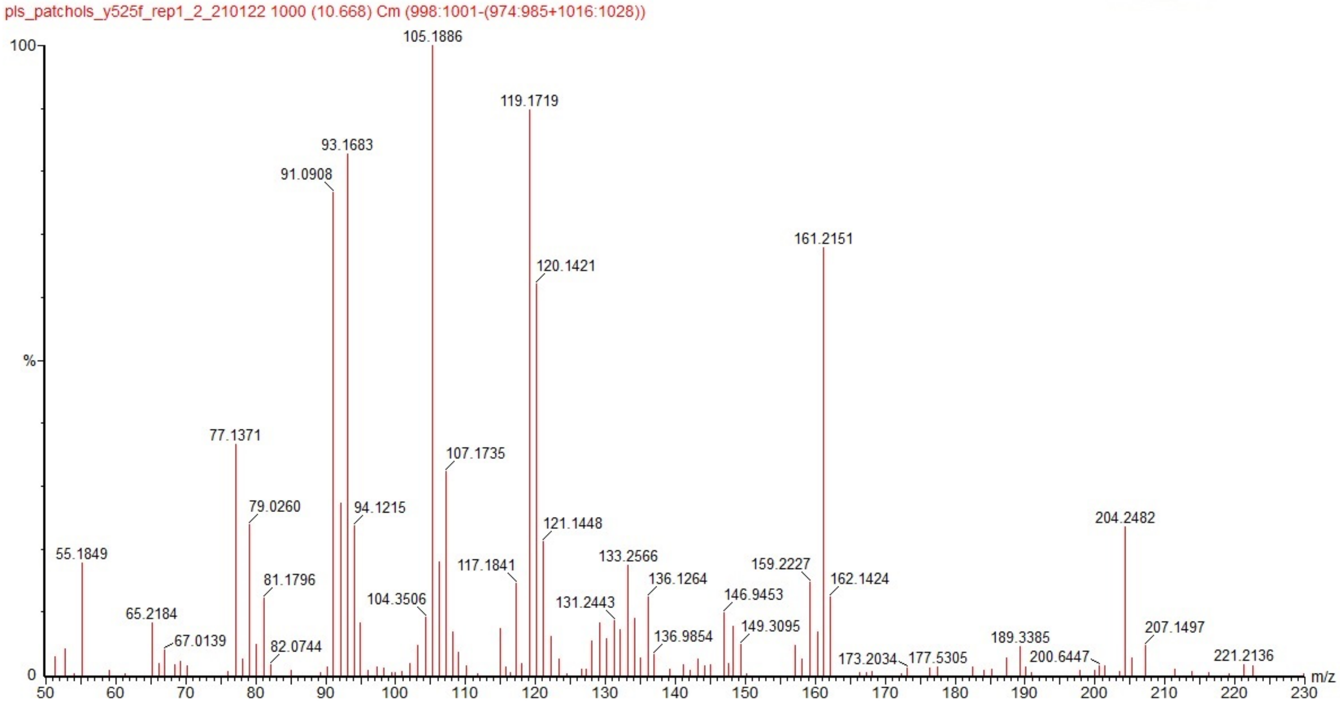

Figure S40. EI Mass spectrum of the compound eluting at 10.66 min in the gas-chromatogram from the incubation of (*E*,*E*)-FDP (**1**) with PTS$_{WT}$ Y525F and Y525A (unknown, **18**).

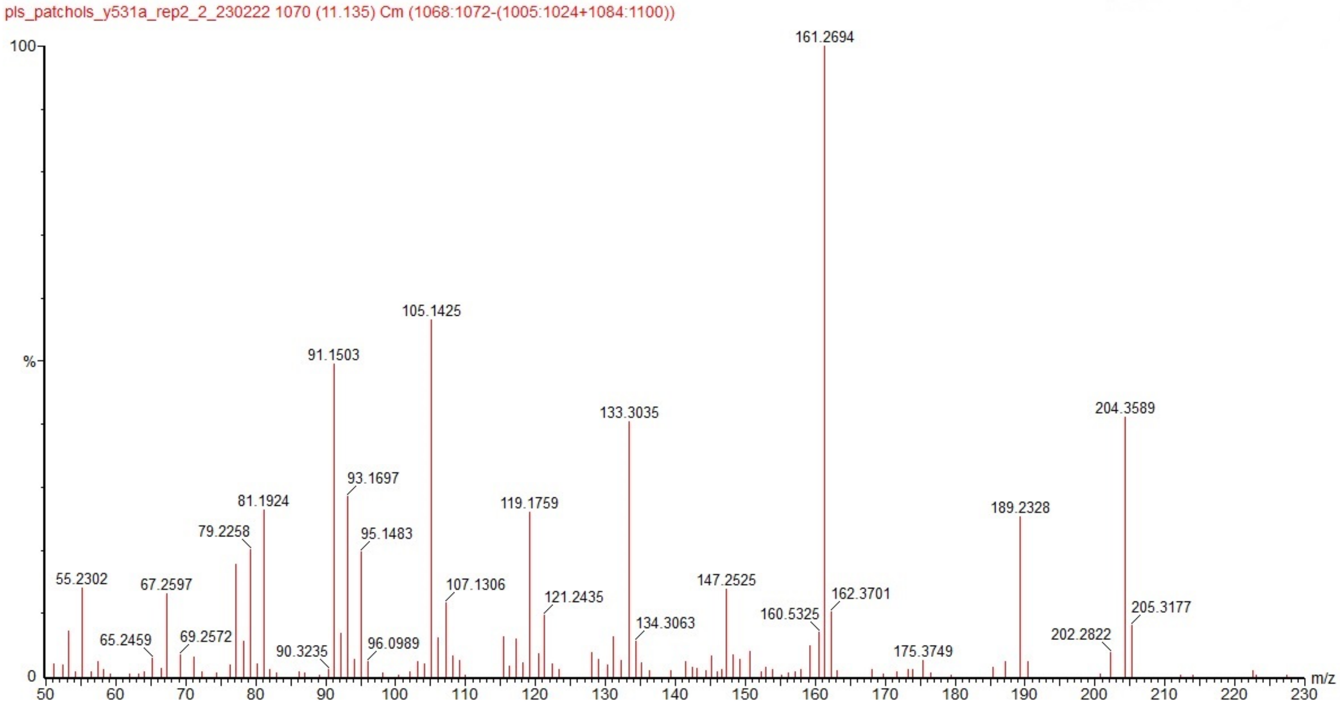

Figure S41. EI Mass spectrum of the compound eluting at 11.13 min in the gas-chromatogram from the incubation of (*E*,*E*)-FDP (**1**) with PTS$_{WT}$ Y531A (unknown, **19**).

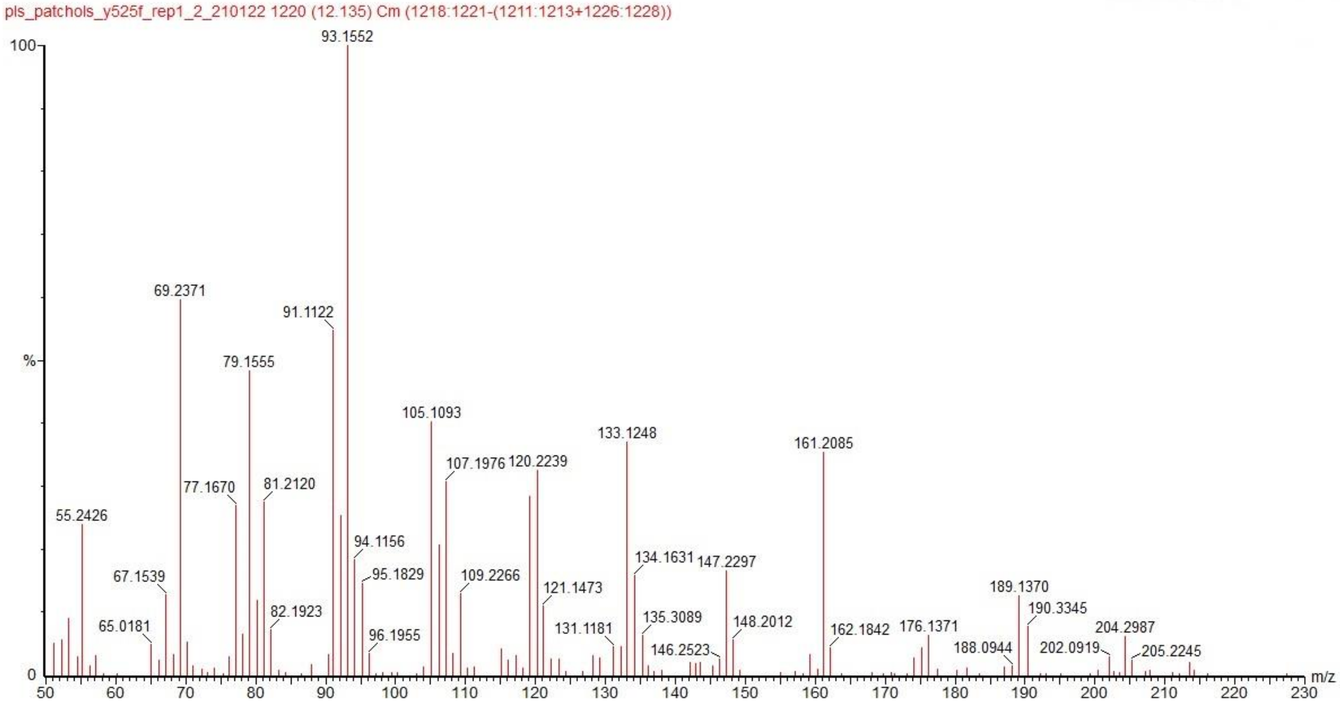

Figure S42. EI Mass spectrum of the compound eluting at 12.13 min in the gas-chromatogram from the incubation of (*E*,*E*)-FDP (**1**) with PTS$_{WT}$ Y525F (Unknown, **20**).

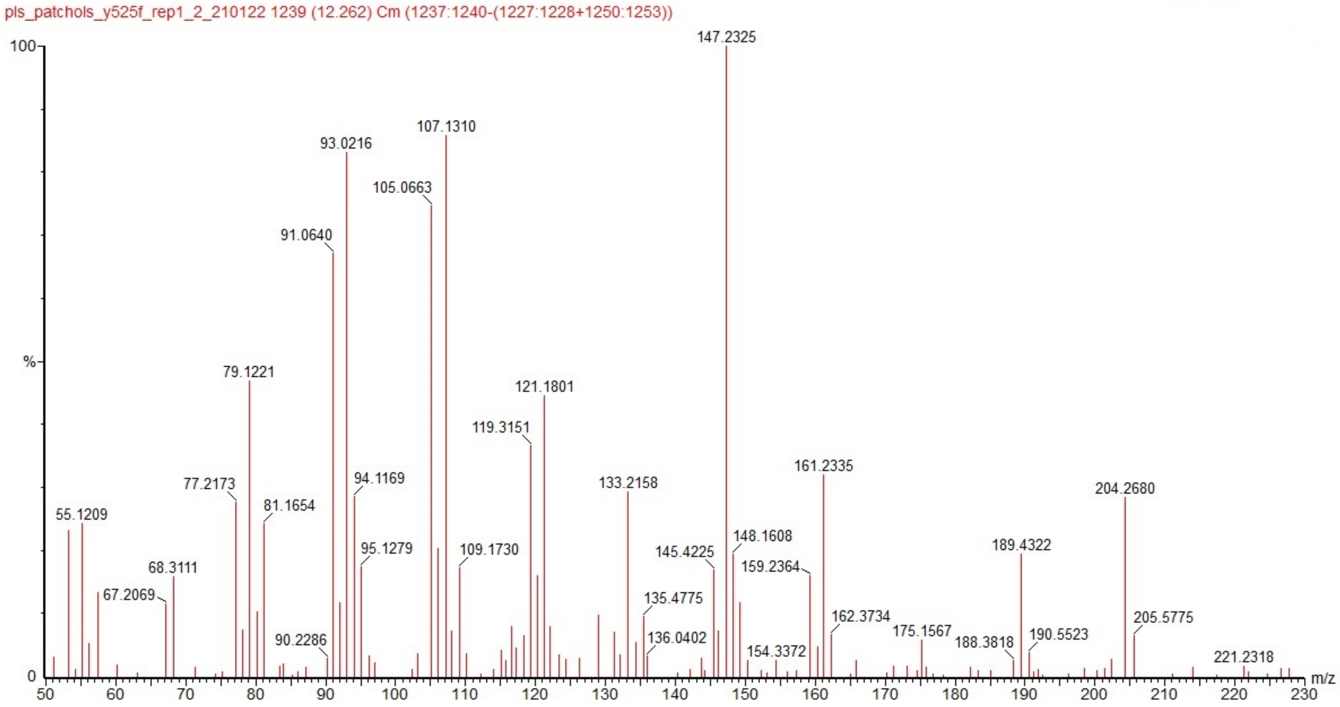

Figure S43. EI Mass spectrum of the compound eluting at 12.26 min in the gas-chromatogram from the incubation of (*E*,*E*)-FDP (**1**) with PTS$_{WT}$ Y525F (unknown, **21**).

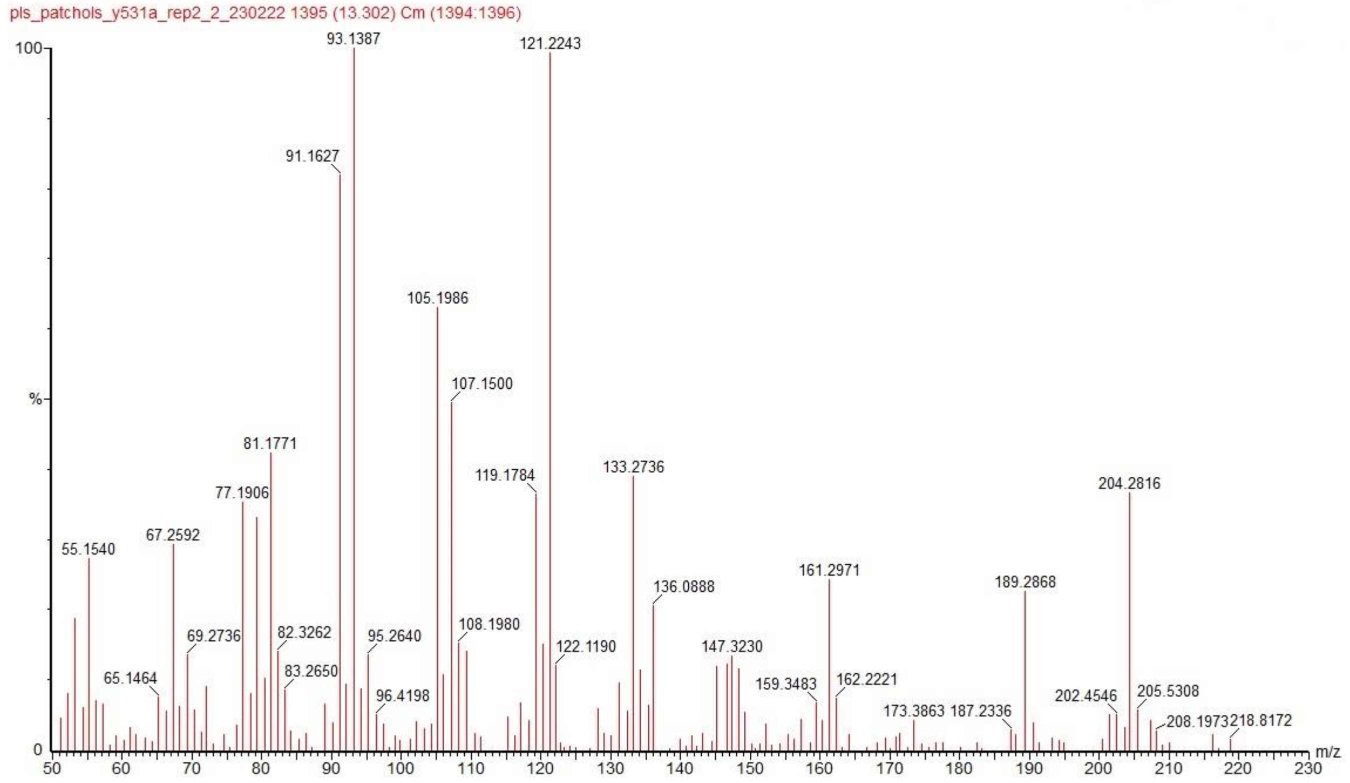

Figure S44. EI Mass spectrum of the compound eluting at 13.30 min in the gas-chromatogram from the incubation of (*E*,*E*)-FDP (**1**) with PTS$_{WT}$ Y531A(unknown, **22**).

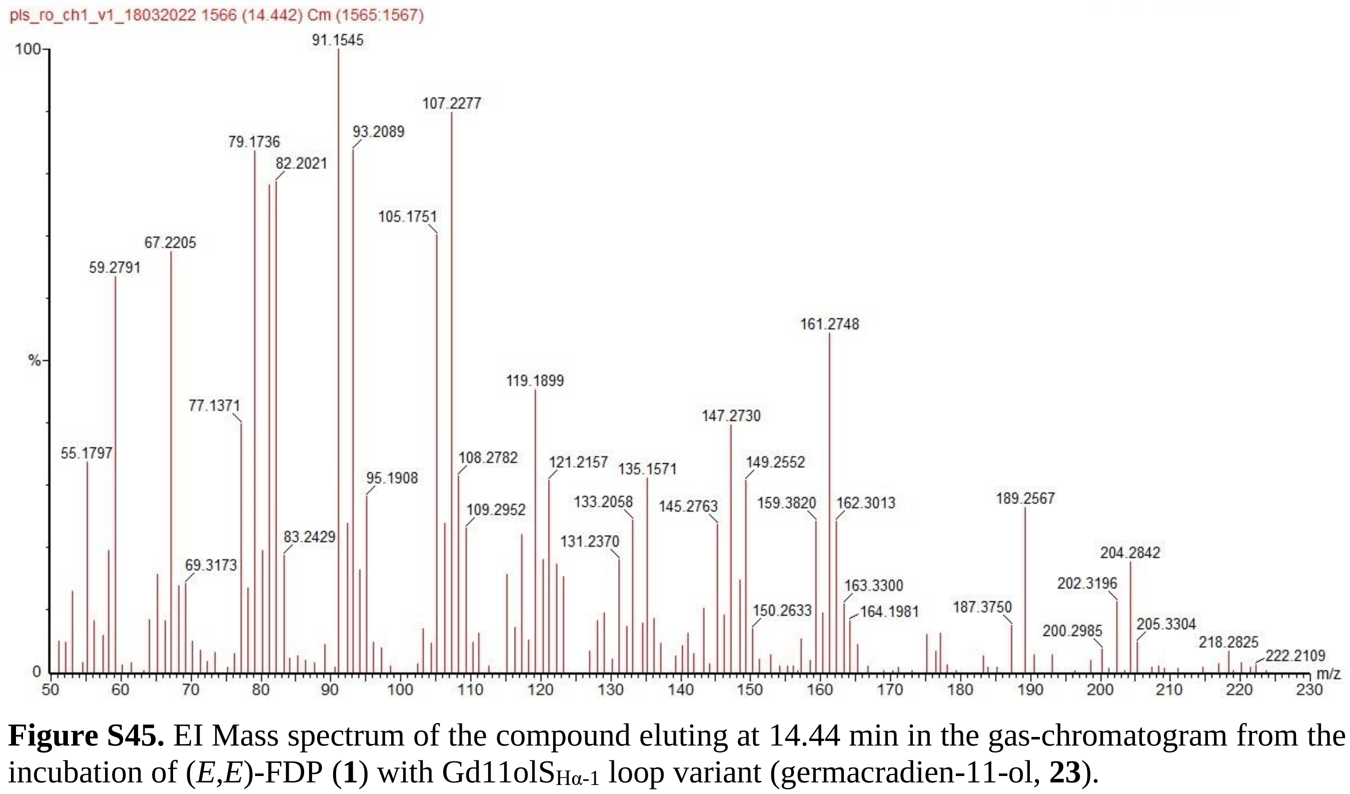

Figure S45. EI Mass spectrum of the compound eluting at 14.44 min in the gas-chromatogram from the incubation of (*E*,*E*)-FDP (**1**) with Gd11olS$_{H\alpha\text{-}1}$ loop variant (germacradien-11-ol, **23**).

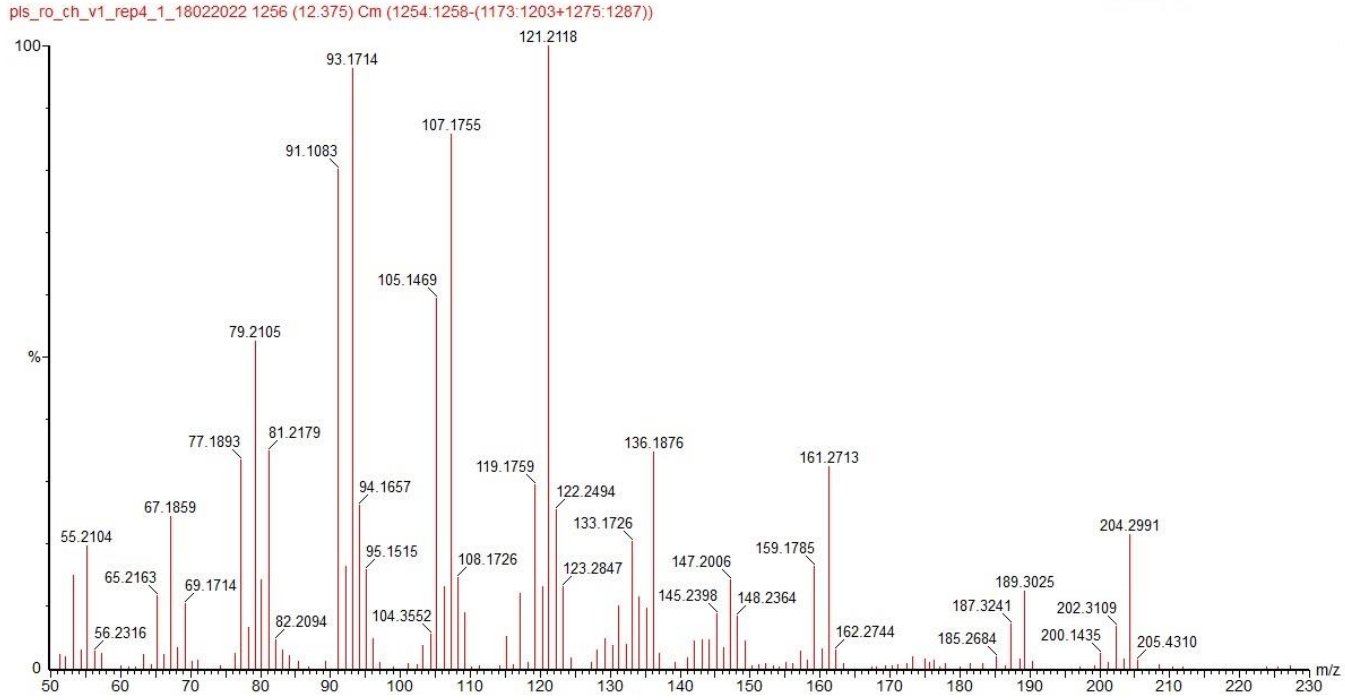

Figure S46. EI Mass spectrum of the compound eluting at 12.37 min in the gas-chromatogram from the incubation of (*E*,*E*)-FDP (**1**) with Gd11olS$_{H\alpha\text{-}1}$ loop variant (isolepidozene, **24**).

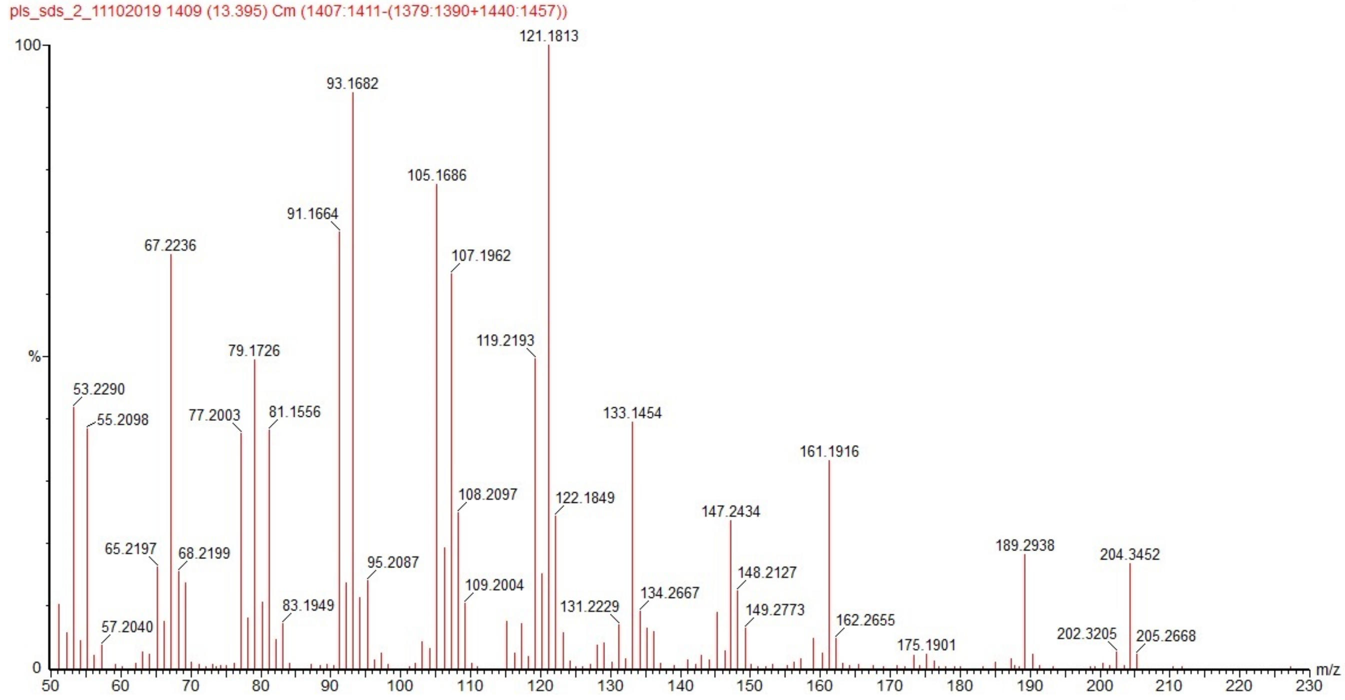

Figure 47: EI Mass spectrum of the compound eluting at 13.38 min in the gas-chromatogram from the incubation of (*E*,*E*)-FDP (**1**) with SdS$_{WT}$ and SdS$_{H\alpha\text{-}1}$ loop variant (8-residues replacement, ^{233}RRGSGYYL^{240} of SdS was replaced by equivalent residues in Gd11olS ^{238}VEDEGELS^{245}), (germacrene B, **25**).

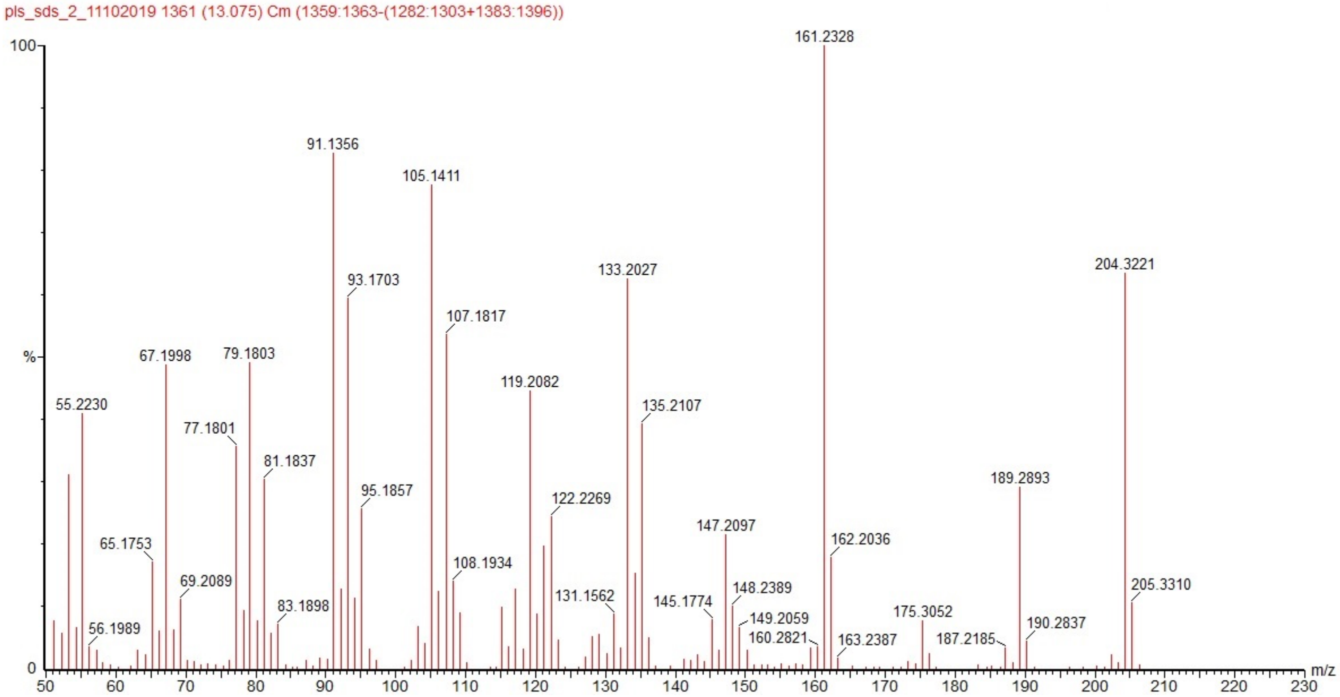

Figure S48: EI Mass spectrum of the compound eluting at 13.06 min in the gas-chromatogram from the incubation of (*E*,*E*)-FDP (**1**) with SDS$_{WT}$ SdS$_{H\alpha\text{-}1}$ loop variant (4-residues replacement, ^{233}RRGS^{236} of SdS was replaced by equivalent residues in Gd11olS ^{238}VEDE^{241}), (selina-4(15),7(11)-diene, **26**).

14. Kinetic Data

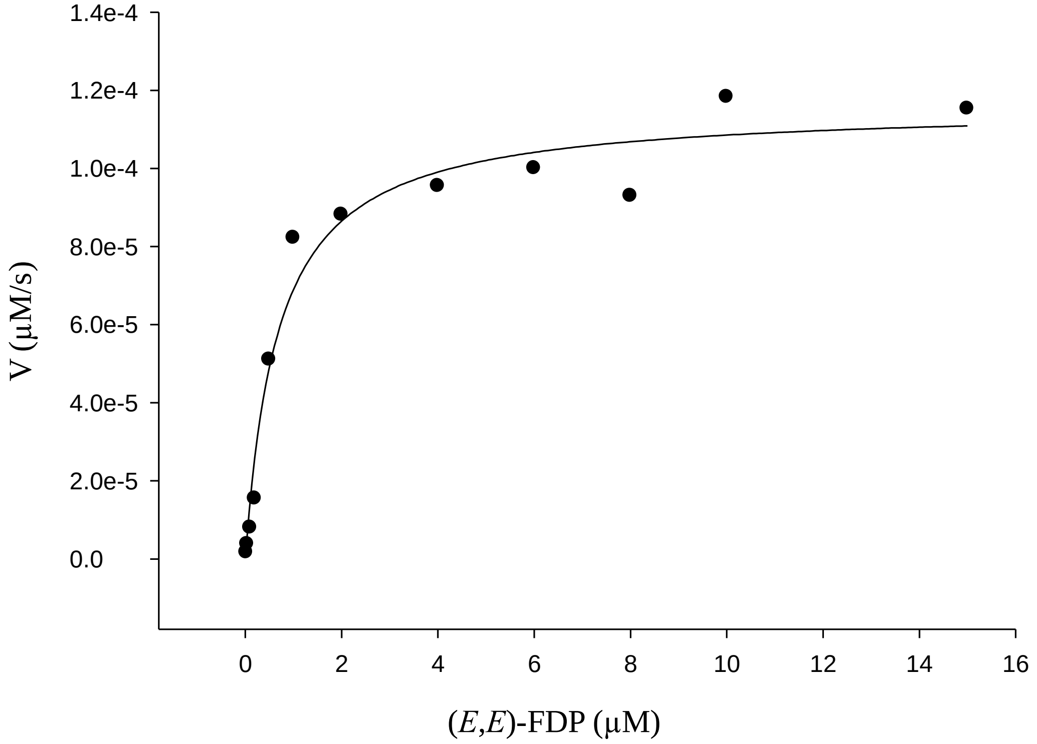

Figure S49. Representative Michaelis-Menten plot for the conversion of [1-^{3}H]-FDP by PTS_{WT}.

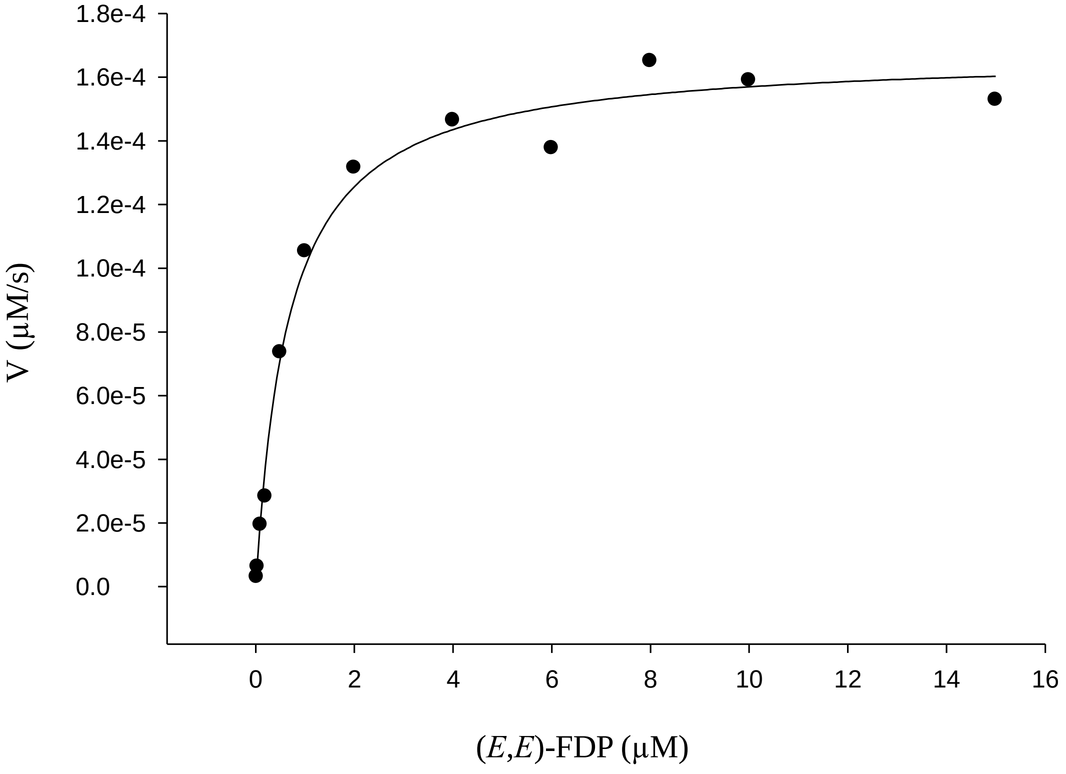

Figure S50. Representative Michaelis-Menten plot for the conversion of [1-^{3}H]-FDP by PTS C405A.

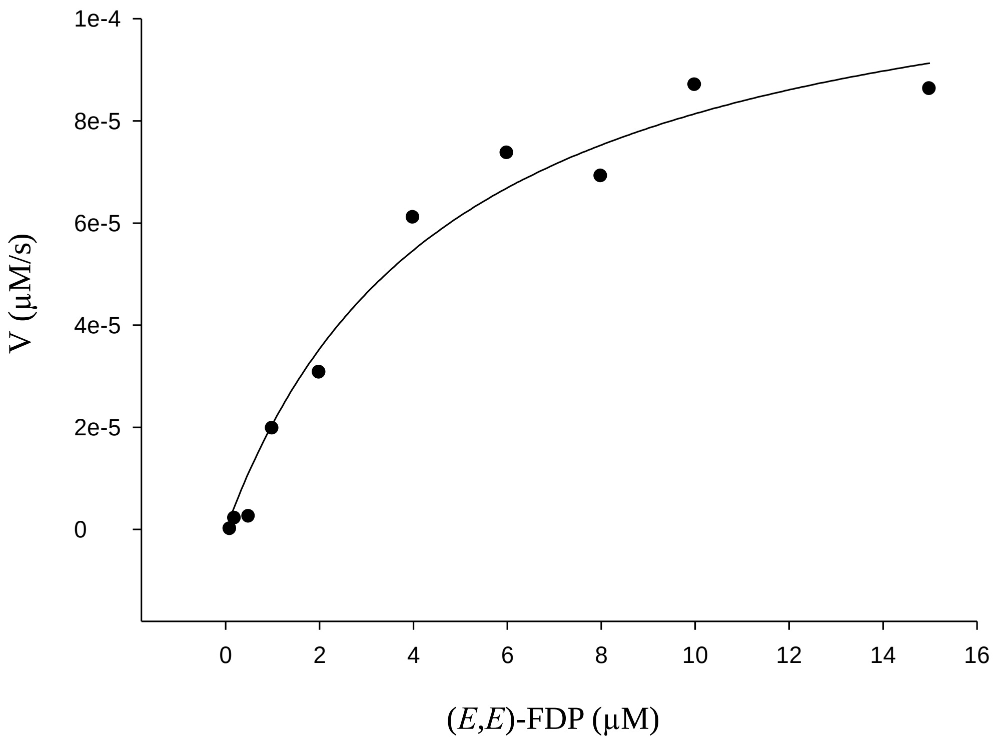

Figure S51. Representative Michaelis-Menten plot for the conversion of [1-^{3}H]-FDP by PTS Y525F.

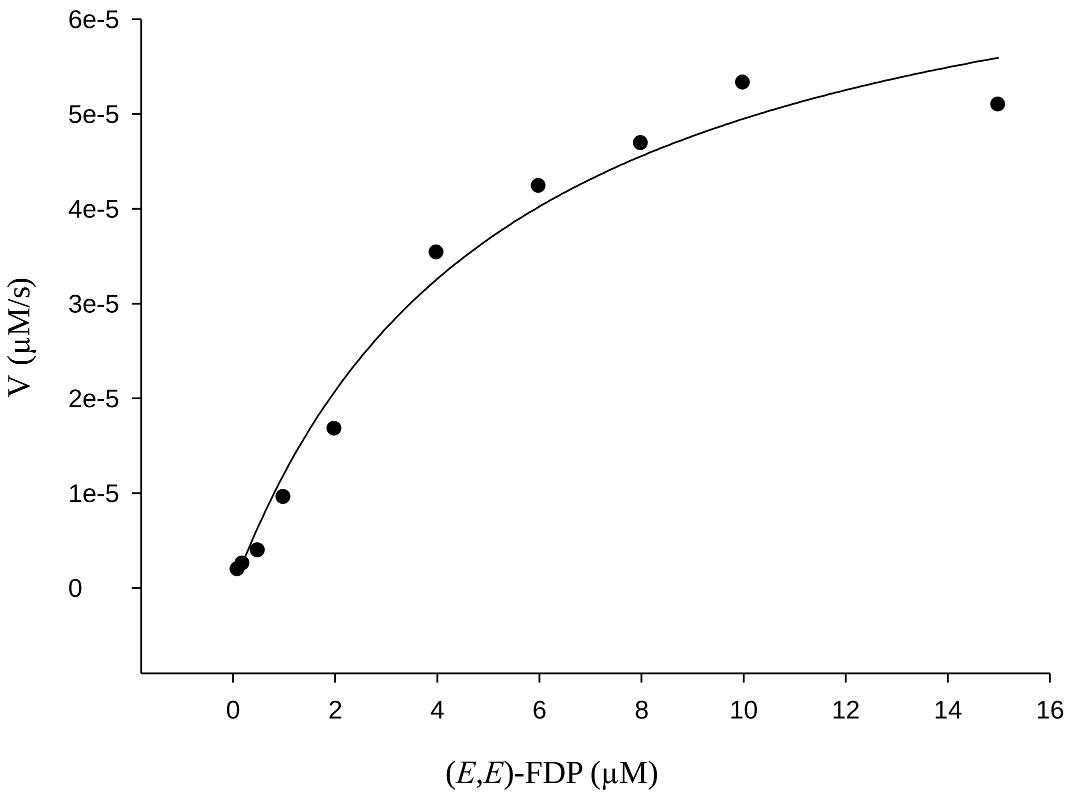

Figure S52. Representative Michaelis-Menten plot for the conversion of [1-^{3}H]-FDP by PTS Y525A.

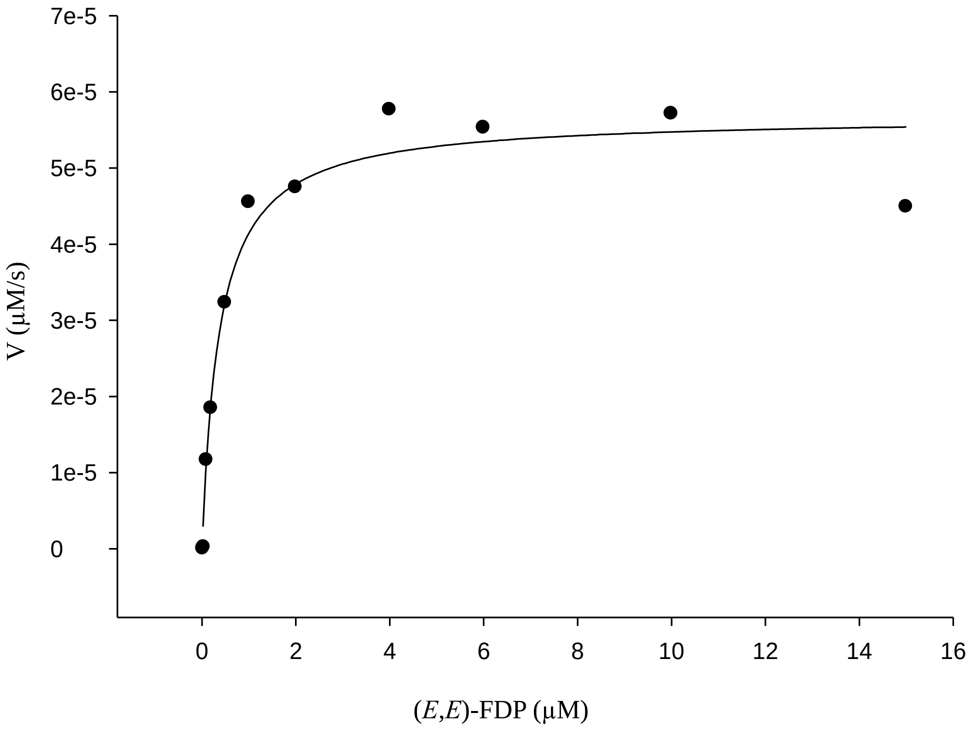

Figure S53. Representative Michaelis-Menten plot for the conversion of [1-^{3}H]-FDP by PTS Y531F.

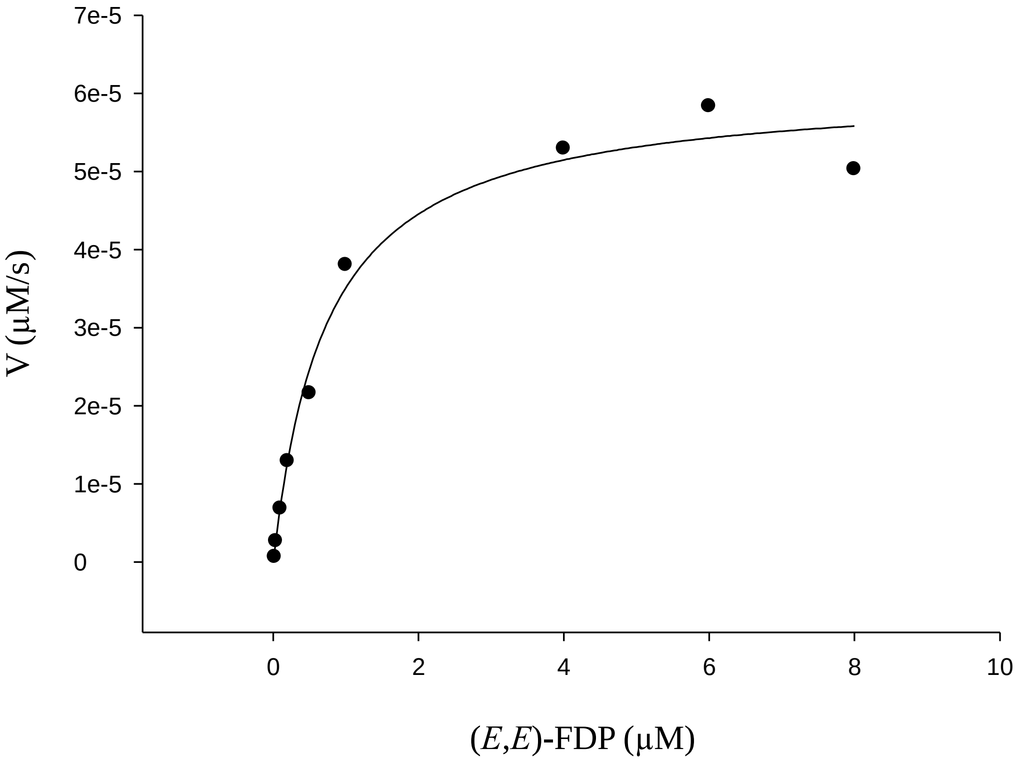

Figure S54. Representative Michaelis-Menten plot for the conversion of [1-^{3}H]-FDP by PTS$_{H\alpha\text{-}1}$ loop variant.

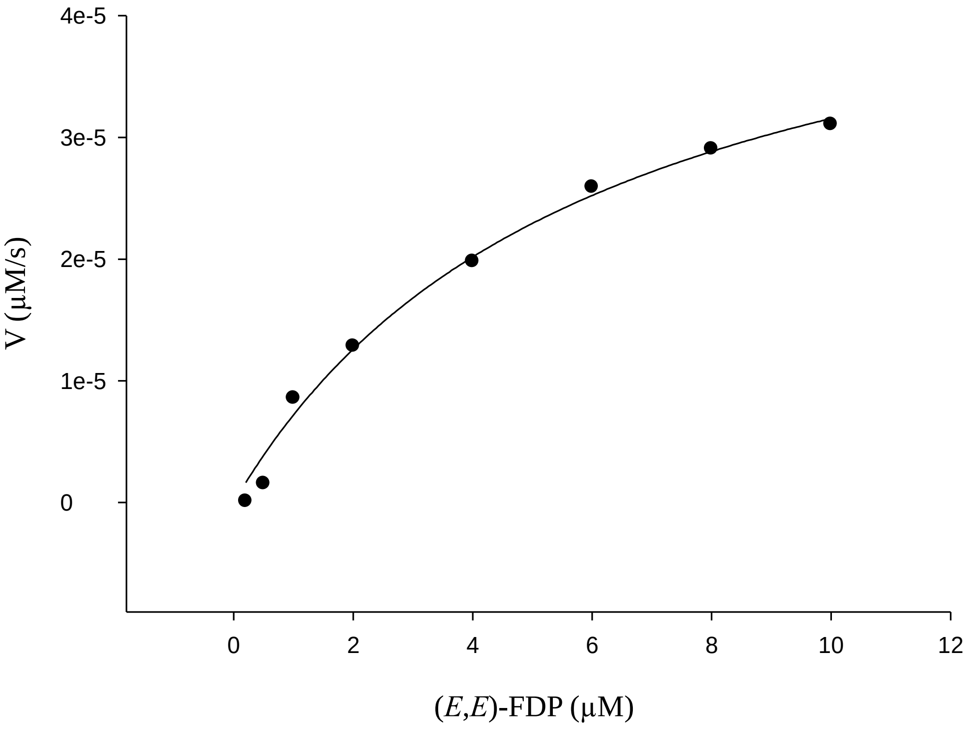

Figure S55. Representative Michaelis-Menten plot for the conversion of [1-^{3}H]-FDP by Gd11olS$_{H\alpha\text{-}1}$ (4-residues replacement, ^{238}VEDE^{241} of Gd11olS was replaced by equivalent residues in SdS ^{233}RRGS^{236}) loop variant.

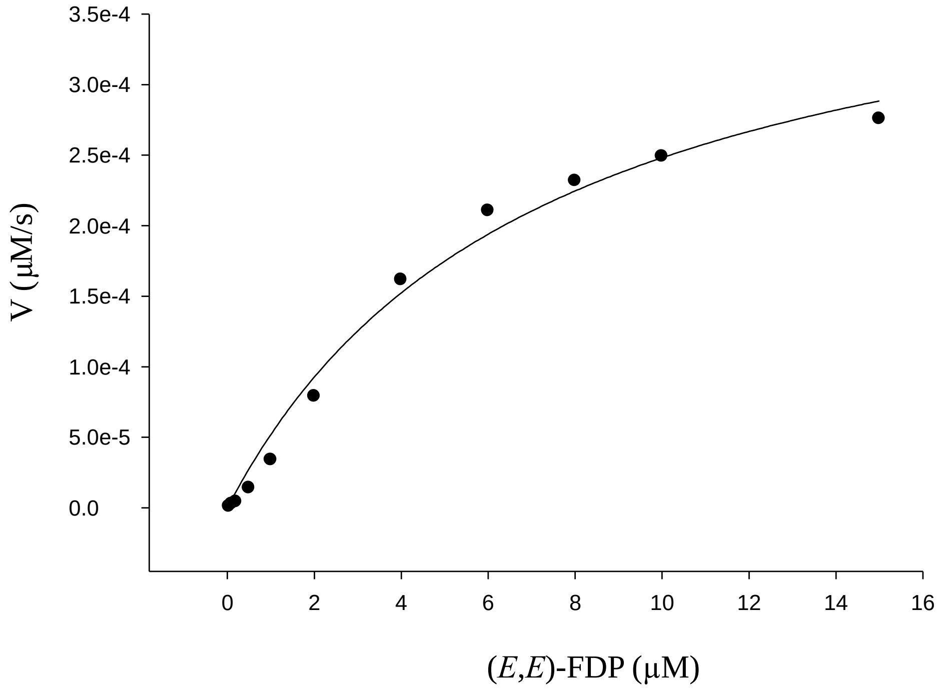

Figure S56. Representative Michaelis-Menten plot for the conversion of [1-^{3}H]-FDP by SdS$_{H\alpha\text{-}1}$ (4-residues replacement, ^{233}RRGS^{236} of SdS was replaced by equivalent residues in Gd11olS ^{238}VEDE^{241}) loop variant.

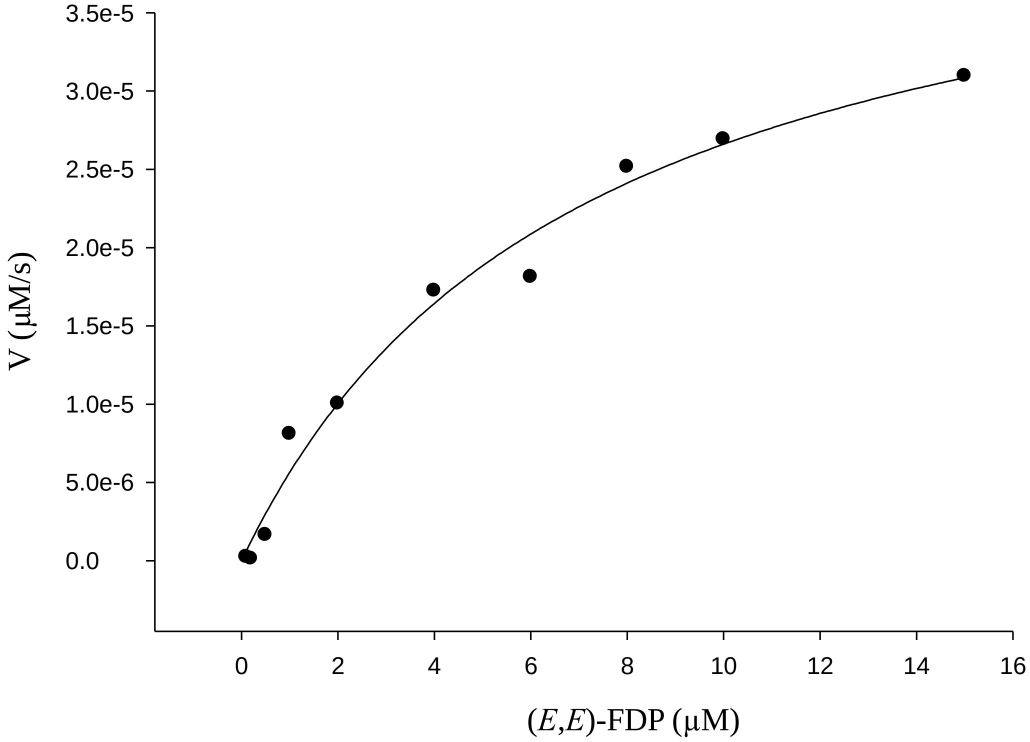

Figure S57. Representative Michaelis-Menten plot for the conversion of [1-^{3}H]-FDP by $SdS_{H\alpha\text{-}1}$ (8-residues replacement, ^{233}RRGSGYYL^{240} of SdS was replaced by equivalent residues in Gd11olS ^{238}VEDEGELS^{245}) loop variant.

15. NMR Spectra

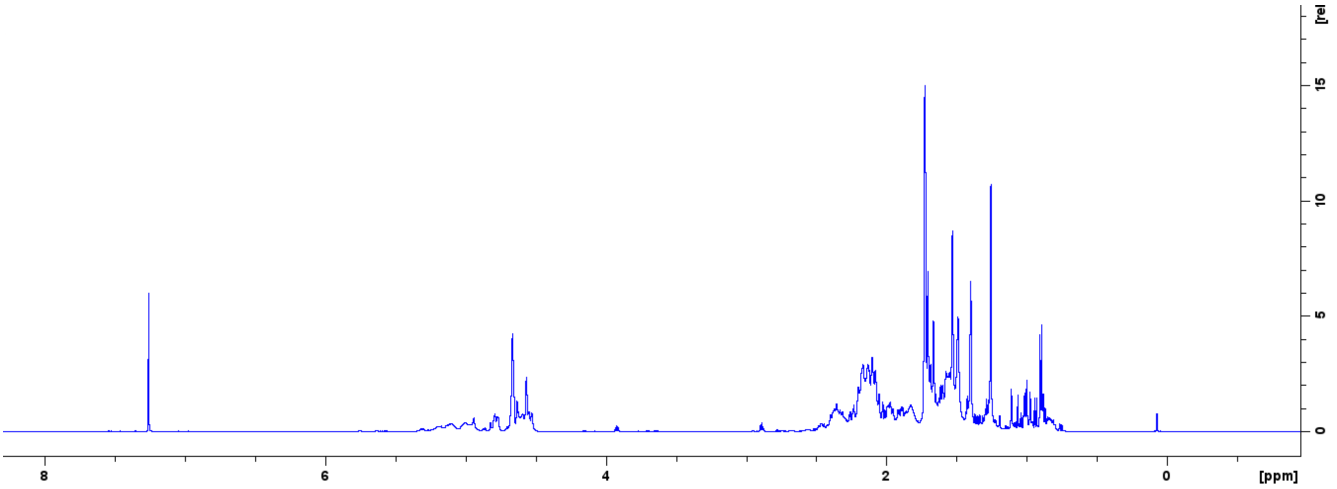

Figure S58. ^{1}H NMR spectrum (500 MHz, $CDCl_3$, 298 K) of assay mixture from $PTS_{H\alpha\text{-}1}$ loop variant producing mixture of **3** (germacrene A) and **4** (α-bulnesene) as a major products along with traces of **5** (α-guaiene) and **12** (β-caryophyllene).

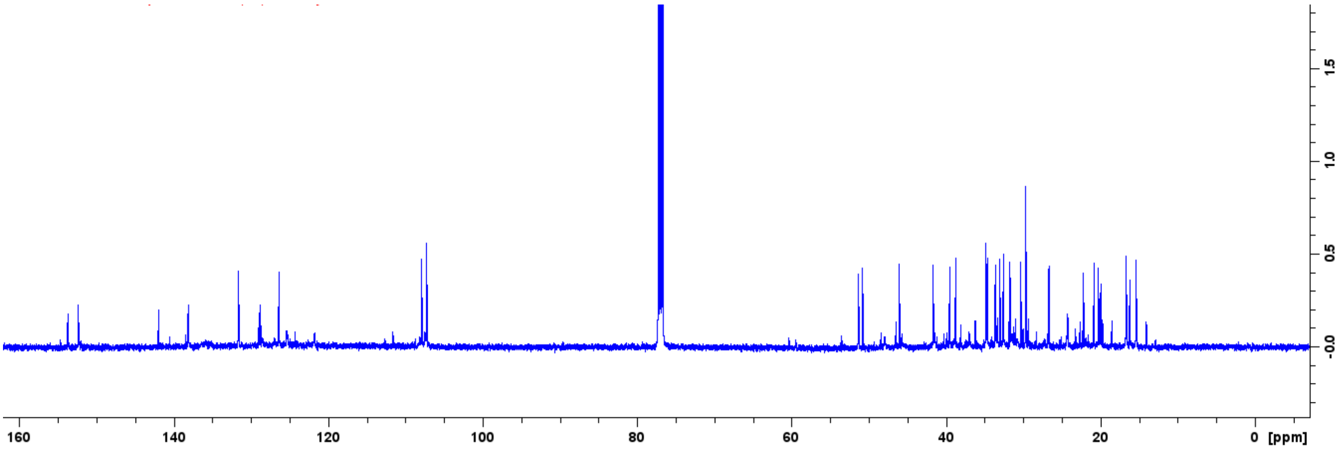

Figure S59. ^{13}C NMR spectrum (500 MHz, CDCl3, 298 K) of assay mixture from $PTS_{H\alpha\text{-}1}$ loop variant producing mixture of **3** (germacrene A) and **4** (α-bulnesene) as a major products along with traces of **5** (α-guaiene) and **12** (β-caryophyllene).

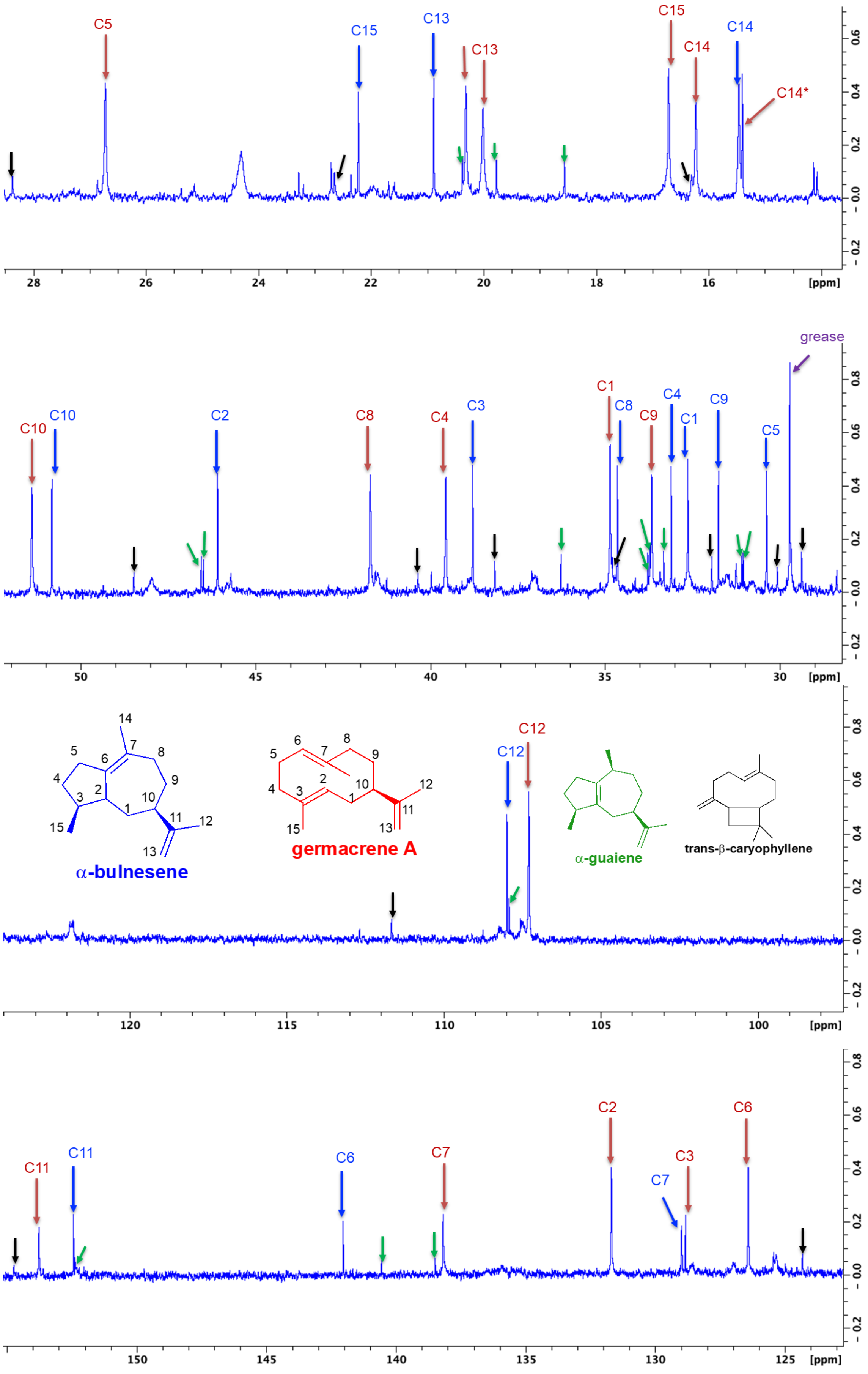

Figure S60. ^{13}C NMR spectrum (500 MHz, CDCl3, 298 K) of assay mixture from $PTS_{H\alpha\text{-}1}$ loop variant producing mixture of **3** (germacrene A) and **4** (α-bulnesene) as a major products along with traces of **5**

(α-guaiene)[10] and **12** (β-caryophyllene)[23]. Carbon peaks denoted by red arrows represent germacrene A (**3**), carbon peaks denoted by blue arrows represent α-bulnesene (**4**), peaks denoted by green arrows represent α-guaiene (**5**) and carbon peaks denoted by black arrows represent β-caryophyllene (**12**). Germacrene A is known to exist in three conformation that exchange on the NMR timescale at room temperature resulting in broad peaks in the ^{13}C NMR spectrum. Some of the unassigned broad peaks in ^{13}C spectra correspond to these conformers of germacrene A.[8,9]

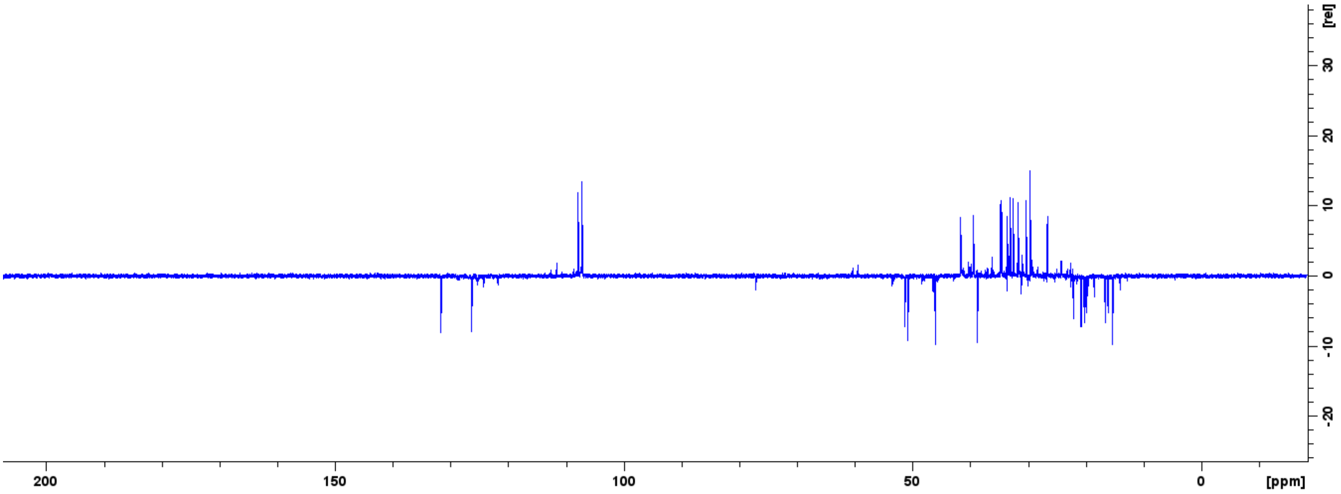

Figure S61. DEPT 135 NMR spectrum (500 MHz, CDCl3, 298 K) of assay mixture from $PTS_{H\alpha\text{-}1}$ loop variant producing mixture of **3** (germacrene A) and **4** (α-bulnesene) as a major products along with traces of **5** (α-guaiene) and **12** (β-caryophyllene).

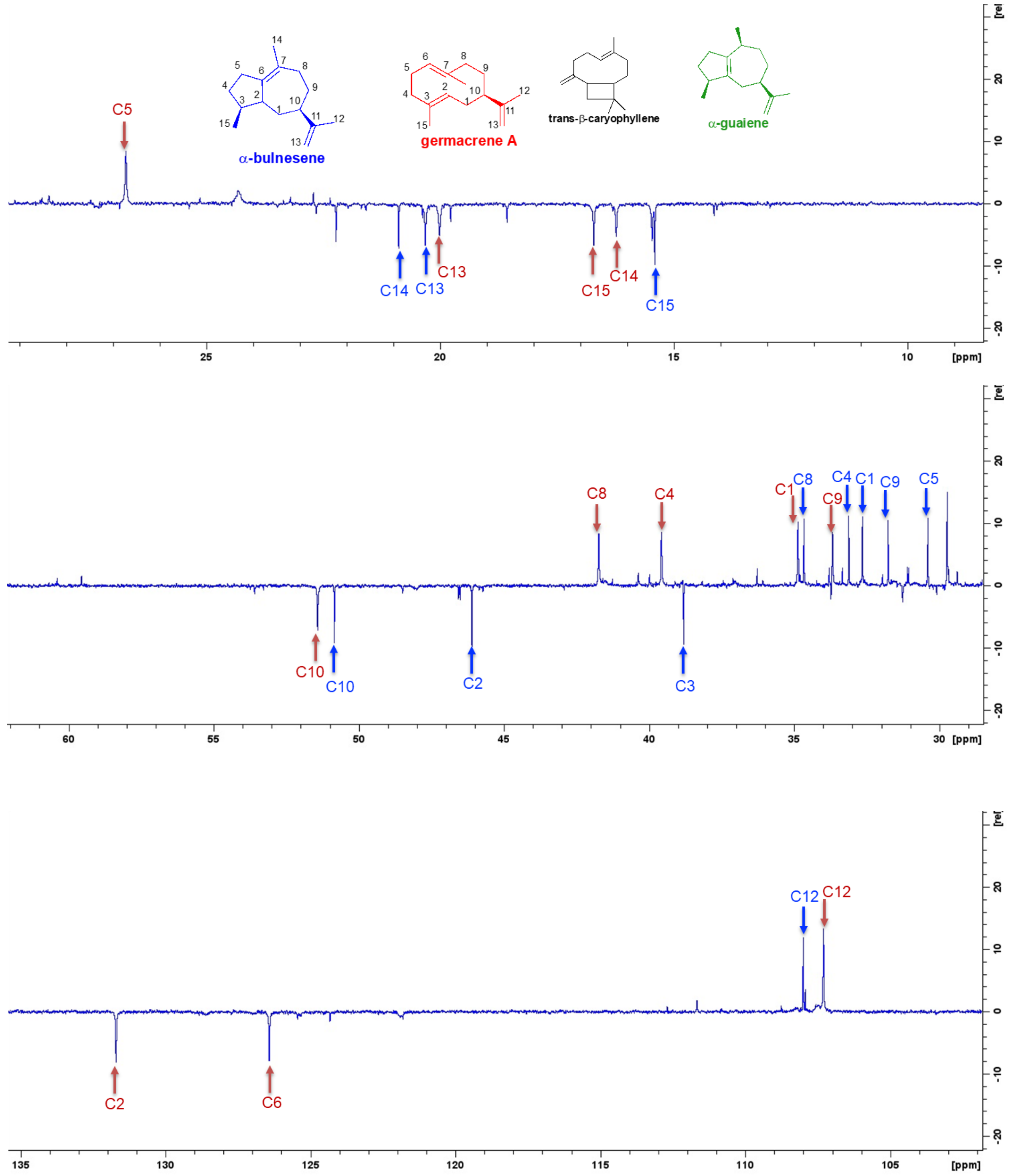

Figure S62. DEPT 135 NMR spectrum (500 MHz, CDCl3, 298 K) of assay mixture from $PTS_{H\alpha\text{-}1}$ loop variant producing mixture of **3** (germacrene A) and **4** (α-bulnesene) as a major products along with traces of **5** (α-guaiene) and **12** (β-caryophyllene). Carbon peaks denoted by red arrows represent germacrene A (**3**), carbon peaks denoted by blue arrows represent α-bulnesene (**4**).

16. References

(1) Deguerry, F.; Pastore, L.; Wu, S.; Clark, A.; Chappell, J.; Schalk, M. The Diverse Sesquiterpene Profile of Patchouli, *Pogostemon cablin*, Is Correlated with a Limited Number of Sesquiterpene Synthases. *Arch. Biochem. Biophys.* **2006**, *454*, 123–136.

(2) Cane, D. E.; Watt, R. M. Expression and Mechanistic Analysis of a Germacradienol Synthase from *Streptomyces coelicolor* Implicated in Geosmin Biosynthesis. *Proc. Natl. Acad. Sci. U. S. A.* **2003**, *100*, 1547–1551.

(3) Jiang, J.; He, X.; Cane, D. E. Biosynthesis of the Earthy Odorant Geosmin by a Bifunctional *Streptomycescoelicolor* Enzyme. *Nat. Chem. Biol.* **2007**, *3*, 711–715.

(4) Baer, P.; Rabe, P.; Fischer, K.; Citron, C. A.; Klapschinski, T. A.; Groll, M.; Dickschat, J. S. Induced-Fit Mechanism in Class I Terpene Cyclases. *Angew. Chemie Int. Ed.* **2014**, *53*, 7652–7656.

(5) Davisson, V. J.; Woodside, A. B.; Neal, T. R.; Stremler, K. E.; Muehlbacher, M.; Poulter, C. D. Phosphorylation of Isoprenoid Alcohols. *J. Org. Chem.* **1986**, *51*, 4768–4779.

(6) Bradford, M. M. A Rapid and Sensitive Method for the Quantitation of Microgram Quantities of Protein Utilizing the Principle of Protein-Dye Binding. *Anal. Biochem.* **1976**, *72*, 248–254.

(7) Grundy, D. J.; Chen, M.; González, V.; Leoni, S.; Miller, D. J.; Christianson, D. W.; Allemann, R. K. Mechanism of Germacradien-4-Ol Synthase-Controlled Water Capture. *Biochemistry* **2016**, *55*, 2112–2121.

(8) Rinkel, J.; Dickschat, J. S. Addressing the Chemistry of Germacrene A by Isotope Labeling Experiments. *Org. Lett.* **2019**, *21*, 2426–2429.

(9) Faraldos, J. A.; Wu, S.; Chappell, J.; Coates, R. M. Conformational Analysis of (+)-Germacrene A by Variable-Temperature NMR and NOE Spectroscopy. *Tetrahedron* **2007**, *63*, 7733–7742.

(10) Rakotonirainy, O.; Gaydou, E. M.; Faure, R.; Bombarda, I. Sesquiterpenes from Patchouli (*Pogostemon cablin*) Essential Oil. Assignment of the Proton and Carbon-13 NMR Spectra. *J. Essent. Oil Res.* **1997**, *9*, 321–327.

(11) Gonzalez, V.; Touchet, S.; Grundy, D. J.; Faraldos, J. A.; Allemann, R. K. Evolutionary and Mechanistic Insights from the Reconstruction of α-Humulene Synthases from a Modern (+)-Germacrene A Synthase. *J. Am. Chem. Soc.* **2014**, *136*, 14505–14512.

(12) Srivastava, P. L.; Escorcia, A. M.; Huynh, F.; Miller, D. J.; Allemann, R. K.; Van Der Kamp, M. W. Redesigning the Molecular Choreography to Prevent Hydroxylation in Germacradien-11-Ol Synthase Catalysis. *ACS Catal.* **2021**, *11*, 1033–1041.

(13) Starks, C. M.; Back, K.; Chappell, J.; Noel, J. P. Structural Basis for Cyclic Terpene Biosynthesis by Tobacco 5-*Epi*- Aristolochene Synthase. *Science.* **1997**, *277*, 1815–1820.

(14) Webb, B.; Sali, A. Comparative Protein Structure Modeling Using MODELLER. *Curr. Protoc. Bioinforma.* **2016**, *2016*, 5.6.1-5.6.37.

(15) Søndergaard, C. R.; Olsson, M. H. M.; Rostkowski, M.; Jensen, J. H. Improved Treatment of Ligands and Coupling Effects in Empirical Calculation and Rationalization of p K a Values. *J. Chem. Theory Comput.* **2011**, *7*, 2284–2295.

(16) Olsson, M. H. M.; SØndergaard, C. R.; Rostkowski, M.; Jensen, J. H. PROPKA3: Consistent Treatment of Internal and Surface Residues in Empirical p K a Predictions. *J. Chem. Theory Comput.* **2011**, *7*, 525–537.

(17) Kovalenko, A.; Hirata, F.; Kinoshita, M. Hydration Structure and Stability of Met-Enkephalin Studied by a Three-Dimensional Reference Interaction Site Model with a Repulsive Bridge Correction and a Thermodynamic Perturbation Method. *J. Chem. Phys.* **2000**, *113*, 9830–9836.

(18) Kovalenko, A.; Hirata, F. Self-Consistent Description of a Metal-Water Interface by the Kohn-Sham Density Functional Theory and the Three-Dimensional Reference Interaction Site Model. *J. Chem. Phys.* **1999**, *110*, 10095–10112.

(19) Sindhikara, D. J.; Yoshida, N.; Hirata, F. Placevent: An Algorithm for Prediction of Explicit Solvent Atom Distribution-Application to HIV-1 Protease and F-ATP Synthase. *J. Comput. Chem.* **2012**, *33*, 1536–1543.

(20) Best, R. B.; Zhu, X.; Shim, J.; Lopes, P. E. M.; Mittal, J.; Feig, M.; MacKerell, A. D. Optimization of the Additive CHARMM All-Atom Protein Force Field Targeting Improved Sampling of the Backbone φ, ψ and Side-Chain X1 and X2 Dihedral Angles. *J. Chem. Theory Comput.* **2012**, *8*, 3257–3273.

(21) Beglov, D.; Roux, B. Finite Representation of an Infinite Bulk System: Solvent Boundary Potential for Computer Simulations. *J. Chem. Phys.* **1994**, *100*, 9050–9063.

(22) Vanommeslaeghe, K.; Hatcher, E.; Acharya, C.; Kundu, S.; Zhong, S.; Shim, J.; Darian, E.; Guvench, O.; Lopes, P.; Vorobyov, I.; Mackerell, A. D. CHARMM General Force Field: A Force Field for Drug-like Molecules Compatible with the CHARMM All-Atom Additive Biological Force Fields. *J. Comput. Chem.* **2010**, *31*, 671–690.

(23) Silva, C. de M. da; Bolzan, A. A.; Mallmann, C. A.; Pozzatti, P.; Alves, S. H.; Heinzmann, B. M. Sesquiterpenoids of Senecio Bonariensis Hook. & Arn., Asteraceae. *Brazilian J. Pharmacogn.* **2010**, *20*, 87–92.
